# GIA imaging of 3-D mantle viscosity based on palaeo sea level observations – Part I: Sensitivity kernels for an Earth with laterally varying viscosity

**DOI:** 10.1093/gji/ggad455

**Published:** 2023-11-21

**Authors:** Andrew J Lloyd, Ophelia Crawford, David Al-Attar, Jacqueline Austermann, Mark J Hoggard, Fred D Richards, Frank Syvret

**Affiliations:** Lamont Doherty Earth Observatory, Columbia University, Palisades, NY 10964, USA; Bullard Laboratories, Department of Earth Sciences, University of Cambridge, Madingley Road, Cambridge CB30EZ, UK; Bullard Laboratories, Department of Earth Sciences, University of Cambridge, Madingley Road, Cambridge CB30EZ, UK; Lamont Doherty Earth Observatory, Columbia University, Palisades, NY 10964, USA; Research School of Earth Sciences, Australia National University, Acton, ACT 0200, Australia; Department of Earth Science and Engineering, Imperial College London, London SW72AZ, UK; Bullard Laboratories, Department of Earth Sciences, University of Cambridge, Madingley Road, Cambridge CB30EZ, UK

**Keywords:** Sea level change, Inverse theory, Rheology: mantle

## Abstract

A key initial step in geophysical imaging is to devise an effective means of mapping the sensitivity of an observation to the model parameters, that is to compute its Fréchet derivatives or sensitivity kernel. In the absence of any simplifying assumptions and when faced with a large number of free parameters, the adjoint method can be an effective and efficient approach to calculating Fréchet derivatives and requires just two numerical simulations. In the Glacial Isostatic Adjustment problem, these consist of a forward simulation driven by changes in ice mass and an adjoint simulation driven by *fictitious* loads that are applied at the observation sites. The theoretical basis for this approach has seen considerable development over the last decade. Here, we present the final elements needed to image 3-D mantle viscosity using a dataset of palaeo sea-level observations. Developments include the calculation of viscosity Fréchet derivatives (i.e. sensitivity kernels) for relative sea-level observations, a modification to the numerical implementation of the forward and adjoint problem that permits application to 3-D viscosity structure, and a recalibration of initial sea level that ensures the forward simulation honours present-day topography. In the process of addressing these items, we build intuition concerning how absolute sea-level and relative sea-level observations sense Earth’s viscosity structure and the physical processes involved. We discuss examples for potential observations located in the near field (Andenes, Norway), far field (Seychelles), and edge of the forebulge of the Laurentide ice sheet (Barbados). Examination of these kernels: (1) reveals why 1-D estimates of mantle viscosity from far-field relative sea-level observations can be biased; (2) hints at why an appropriate differential relative sea-level observation can provide a better constraint on local mantle viscosity and (3) demonstrates that sea-level observations have non-negligible 3-D sensitivity to deep mantle viscosity structure, which is counter to the intuition gained from 1-D radial viscosity Fréchet derivatives. Finally, we explore the influence of lateral variations in viscosity on relative sea-level observations in the Amundsen Sea Embayment and at Barbados. These predictions are based on a new global 3-D viscosity inference derived from the shear-wave speeds of GLAD-M25 and an inverse calibration scheme that ensures compatibility with certain fundamental geophysical observations. Use of the 3-D viscosity inference leads to: (1) generally greater complexity within the kernel; (2) an increase in sensitivity and presence of shorter length-scale features within lower viscosity regions; (3) a zeroing out of the sensitivity kernel within high-viscosity regions where elastic deformation dominates and (4) shifting of sensitivity at a given depth towards distal regions of weaker viscosity. The tools and intuition built here provide the necessary framework to explore inversions for 3-D mantle viscosity based on palaeo sea-level data.

## 1 INTRODUCTION

Geophysicists have gone to great lengths to image Earth’s interior using observations of seismic wave propagation (e.g. Nolet 2008; Tromp 2019), gravitational (e.g. Sjöberg & Bagherbandi 2017) and electromagnetic (e.g. Tikhonov 1950; Chave & Jones 2012) fields, as well as its response to deformation by both internal (e.g. Pollitz 2001; Forte & Mitrovica 1996) and external forces (e.g. moon and sun; Nakada & ichiro Karato 2012; Lau *et al.*^2017^). In doing so, they provide constraints on physical parameters that fundamentally control the behaviour of our planet across a range of spatial and temporal scales. With advancements in imaging techniques, computational resources, and observational data sets, imaging of these parameters has evolved from simple, spherically symmetric 1-D models to increasingly complex 3-D structural models. Nevertheless, after nearly a century of research, imaging of Earth’s viscous structure has remained restricted to 1-D radial models (e.g. Haskell 1935; Mitrovica 1996; Lau *et al.*^2016^; Argus *et al.*^2021^). These models generally exploit observations of Glacial Isostatic Adjustment (GIA), which is the viscoelastic deformation of the solid Earth as well as changes to its gravitational field and rotational axis in response to the evolving surface loads of the ice sheets and oceans. This process is most reliably constrained by observations of palaeo sea level, but the use of these observations to image 3-D mantle viscosity has been hindered by a sparsity of data coverage, a lack of appropriate standardization procedures (Khan *et al.*^2019^), and perhaps most importantly, the absence of an efficient inversion scheme.

The influence of 3-D viscosity structure on GIA has been recognized for the past few decades (e.g. Gasperini *et al.*^1990^; Whitehouse 2018), but has seen an accelerated interest in recent years driven by a desire to better understand the interactions between the solid Earth and the cryosphere (e.g. Kaufmann *et al.*^2005^; Gomez *et al.*^2015^; Whitehouse *et al.*^2019^) or more broadly the hydrosphere (e.g. Wu 2006; Austermann *et al.*^2013^; Li *et al.*^2020^; Bagge *et al.*^2021^). The large range of viscosity heterogeneity (∼10^18^−10^23^ Pa·s) imaged by regional GIA studies (e.g. Nield *et al.*^2014^, ^2016^; Barletta *et al.*^2018^; Austermann *et al.*^2020^) suggests that Earth’s viscous response occurs over timescales of years to thousands of years and at length scales of tens to many thousands of kilometres. Simulations show that such lateral variations in viscosity can give rise to complex patterns of deformation that are not readily reproduced by a spherically symmetric viscosity model unless the ice history is substantially modified (e.g. Kaufmann *et al.*^2005^; Klemann *et al.*^2007^). Likewise, the 3-D viscosity structure of subduction zones can influence local relative sea level and have a profound impact on its interpretation and hence any associated implications for ice history (Austermann *et al.*^2013^). Thus, there is a clear need for an accurate representation of Earth’s 3-D viscous structure in order to both improve GIA models (their past and/or future predictions) and also to better understand how GIA observations probe Earth’s viscous structure. This need has created two main avenues for constraining Earth’s 3-D viscosity structure. Those studies that *infer* viscosity from other physical parameters, such as seismic wave speeds, and those that *image* viscosity directly from observations of viscous processes like GIA. Here, we utilize the former and will establish the latter in Lloyd *et al.* (in preparation), but note the two need not be mutually exclusive.

Inference-based approaches primarily convert seismic tomography models of shear-wave speed to viscosity by way of temperature using constitutive relationships and material parameters derived from laboratory experiments (e.g. Priestley & McKenzie 2013; Yamauchi & Takei 2016; Richards *et al.*^2020^; Austermann *et al.*^2021^; Ivins *et al.*^2021^; Paxman *et al.*^2023^). Although such approaches benefit from the high resolution and broad spatial coverage of seismic tomography, they also inherit the assumptions and uncertainties associated with the tomographic inversion, constitutive relationships and material parameters. Accounting for these effects is non-trivial and, in many instances, impractical, but can be combated with calibration schemes that identify inferences satisfying a number of well-known, independent solid Earth observations (Li *et al.*^2018^; Richards *et al.*^2020^; Ivins *et al.*^2021^). Nevertheless, assumptions concerning the physical state of the mantle and hence the origin of the seismic anomalies (e.g. temperature, composition, fluids, melt, etc.), as well as the deformation mechanisms that are activated by the transfer of seismic energy (e.g. dislocation creep, diffusion creep, grain boundary sliding, etc.), result in a wide range of plausible viscosity inferences (Ivins *et al.*^2021^; Hazzard *et al.*^2023^). This aspect is further compounded due to the fact that GIA models include not only a solid Earth response (i.e. viscoelastic structure and rheology), but also an ice history that drives this response and hence, the two are strongly intertwined. Thus, there is still no guarantee that the resulting 3-D viscosity inference will provide a better fit to GIA observations due to errors in either component of the GIA model (e.g. Bagge *et al.*^2021^).

In this study, we begin exploring how and by which deformational processes palaeo sea-level observations sense Earth’s viscosity structure, and how these sensitivities are coupled to the assumed viscosity structure and ice history. Despite the potential inaccuracy of combining reconstructed ice histories with an independent viscosity structure, we elect to use an inference of 3-D mantle viscosity in combination with a published ice history. For these purposes, we apply the adjoint method and build off the work of Al-Attar & Tromp (2013) and Crawford *et al.* (2018). This effort ultimately lays the foundation for imaging 3-D mantle viscosity directly from GIA observations and, to aid in its development, we will draw parallels to, and borrow from, seismology. We begin by briefly explaining why the adjoint method is an appropriate tool for this problem and provide a summary of the necessary equations for defining and calculating viscosity and initial sea-level Fréchet derivatives (Section 2), a topic that is covered in more detail within Appendix A. In Section 3 we expand the rate formulation of the forward and adjoint GIA problem to consider relative sea-level observations and lateral variations in viscosity. Next, we discuss how the adjoint method can be used in a gradient based optimization scheme to recalibrate the initial sea level and ensure simulation compatibility with *known* present-day sea level (Section 4). Following this theoretical and methodological development, we discuss the forward and adjoint GIA simulation setup and a new inference of 3-D viscosity obtained by applying the approach of Richards *et al.* (2020) and Austermann *et al.* (2021) to the shear wave speeds of GLAD-M25 (Bozdağ *et al.*^2016^; Lei *et al.*^2020^; Section 5). Using these new tools, we demonstrate the initial sea-level recalibration and examine how the evolution of sea level is influenced by different viscosity models and ocean-loading histories (Sections 6.1 and 6.2). This demonstration is followed by a discussion of viscosity Fréchet derivatives for observations of both absolute and relative sea level that focuses on identifying how physical processes (e.g. ocean siphoning and expulsion) are manifested, their big-picture implications, and how these two observation types differ in their sensitivity to viscosity (Section 6.3). Through these simple examples that adopt a 1-D radial viscosity model, we aim to begin building the necessary intuition and skills for both observational and theoretical scientists to *read* viscosity Fréchet derivatives, much like seismologists *read* seismograms. We then examine the effect of a more realistic 3-D viscosity model on the viscosity Fréchet derivatives for observations of relative sea level (Section 6.4). Finally, the methods, results, and intuition built herein are used to inform a companion paper (Lloyd *et al.* in preparation) that explores strategies for imaging 3-D mantle viscosity with synthetic palaeo sea-level data.

## 2 REVIEW OF FRÉCHET DERIVATIVES FOR THE GIA PROBLEM

The first step towards data-driven inversions of GIA observations is to determine how a potential observation changes in response to a change in the relevant model parameters. This quantity is called a *Fréchet derivative* and can be efficiently calculated using the adjoint method. Al-Attar & Tromp (2013) and Crawford *et al.* (2018) developed the necessary mathematical theory linking a rate formulation of the GIA problem to the adjoint method and in Appendix A we provide a detailed review of this work and its key assumptions. In addition, Appendix A includes a table defining the variables of the forward and adjoint GIA problem (Table A1). This review is accompanied by a schematic overview of the adjoint method that sheds light on the derivation of the adjoint equations, including the origin of the *fictitious* loads and the time-reversed nature of the simulation, as well as the derivation of Fréchet derivatives for relevant model parameters (e.g. viscosity). We encourage the interested reader to consult Appendix A, but in the interests of brevity, restrict ourselves here to a conceptual description of Fréchet derivatives and a review of the relationships required to calculate them with respect to viscosity and initial sea level.

### 2.1 A conceptual description of Fréchet derivatives

For simplicity, let us first consider the viscosity, *η*, of Earth’s mantle as the only free model parameter. For a given viscosity structure, we can solve the forward GIA problem in order to obtain all possible surface observables and evaluate a scalar-valued functional, *F*, which could be an observation (e.g. sea level, *SL*) or a suitable designed misfit function. Thus, the value of *F* implicitly depends on the viscosity, *η*, and can be written as *F*(*η*).

If a viscosity perturbation, δ*η*, is applied to the adopted viscosity structure, we can to first-order write


(1) \begin{eqnarray*}
F(\eta + \delta \eta ) = F(\eta ) + \int _{M} K_{\ln \eta }\delta \ln \eta \, \mathrm{d}V + \cdots ,
\end{eqnarray*}

where $\delta \ln \eta = \frac{\delta \eta }{\eta }$, $\, \mathrm{d}V$ indicates a volume integral over the region, *M*, and ⋅⋅⋅ indicates higher-order terms associated with the perturbation δ*η*. Note that the use of ln *η* as a model parameter rather than *η* is a choice that is made for convenience. The function, *K*_ln _*_η_*, is known as the Fréchet derivative of *F* with respect to ln *η*. Furthermore, it is also common and useful to rewrite eq. (1) as


(2) \begin{eqnarray*}
\delta F = \int _{M} K_{\ln \eta }\delta \ln \eta \, \mathrm{d}V,
\end{eqnarray*}

where it is understood that δ*F* is the first-order change in the functional *F* in response to a perturbation, δln *η*. Written in this form, we can intuitively understand the meaning of the Fréchet derivative (i.e. *K*_ln _*_η_*). In this example, positive (negative) values of *K*_ln *η*_ indicates that an increase in viscosity at those locations within the Earth will lead to an increase (decrease) in *F* at the observation site. The corresponding size of the change in *F* depends on the magnitude of *K*_ln _*_η_*. Thus, by plotting the Fréchet derivative, *K*_ln _*_η_*, for a given functional we can visualize, to first-order accuracy, how its value is influenced by a change in viscosity.

In the event that more than one model parameter is considered, the Fréchet derivative of *F* with respect to each of the model parameters can be introduced. For example, in addition to viscosity, we can include initial sea level, *SL*_0_, as a further model parameter (Section 4). In the forward GIA problem, the initial sea level enters as an initial condition describing sea level (i.e. the negative of topography) at the beginning of the simulation. Our functional, *F*, then has an implicit dependence on both *η* and *SL*_0_ and we can generalize eq. (1) to


(3) \begin{eqnarray*}
F(\eta + \delta \eta ,SL_{0} + \delta SL_{0}) &=& F(\eta ,SL_{0}) + \int _{M} K_{\ln \eta }\delta \ln \eta \, \mathrm{d}V \\
&+& \int _{\partial M} K_{SL_{0}}\delta SL_{0} \, \mathrm{d}S + \cdots ,
\end{eqnarray*}

where $K_{SL_{0}}$ is the Fréchet derivative of *F* with respect to the initial sea level and $\, \mathrm{d}S$ indicates a surface integral over the region ∂*M*. Recall that we write and retain only first-order terms, hence cross-terms between δln *η* and δ*SL*_0_ are represented by ⋅⋅⋅. Nevertheless, it is important to remember that both Fréchet derivatives depend on the unperturbed values of the model parameters (i.e. *η* and *SL*_0_). Thus, their physical interpretation remains the same, but it is understood that they express the linearized sensitivities to one model parameter when the other parameters are held fixed.

### 2.2 Fréchet derivatives in GIA

A simple, albeit brute-force, approach to determining these Fréchet derivatives is the finite-difference method and it is this method that has historically been used to compute kernels (Appendix C1). In this approach, the model parameters are first expressed using a finite-dimensional basis that is either deemed to be physically appropriate or has been accepted for pragmatic reasons. Supposing that there are *n*-degrees of freedom in this basis, a cost of *n* + 1 individual GIA simulations are required to compute a single Fréchet derivative: one simulation for the unperturbed problem and *n* additional simulations that individually perturb each of the model parameters in turn (e.g. Mitrovica & Peltier 1991b; Paulson *et al.*^2005^; Wu 2006). Given that *n* is large for GIA simulations that attempt to capture realistic variations in 3-D viscosity structure, that these simulations are computationally expensive (e.g. Latychev *et al.*^2005^), and that within an iterative inversion, these Fréchet derivatives need to be computed many times, it is clear that such an approach is impractical. Instead, we follow the lead of seismic tomography (e.g. Tromp *et al.*^2004^; Fichtner *et al.*^2006^) and use the adjoint method to calculate Fréchet derivatives with just two numerical simulations: a forward simulation driven by the ice history and a time-reversed adjoint simulation driven by *fictitious* loads applied at the observation sites at appropriate times (Al-Attar & Tromp 2013; Crawford *et al.*^2018^). For completeness, we show in Appendix C2 that these two approaches obtain the same result and that the resulting Fréchet derivatives can be used to predict the change in the functional (e.g. relative sea level) for a given model perturbation (e.g. viscosity).

As shown in Appendix A2, the Fréchet derivative for a given model parameter (e.g. viscosity) can be obtained by perturbing the *Lagrangian* (eq. A15) with respect to that parameter. If we assume a Maxwell rheology, then the Fréchet derivative with respect to ln *η* takes the form


(4) \begin{eqnarray*}
K_{\ln {\eta }} = \int _{t_0}^{t_1} \frac{1}{2 \eta } \boldsymbol{\tau } :\boldsymbol{\tau }^{\dagger } \, \mathrm{d}t,
\end{eqnarray*}

where *t*_0_ is the time corresponding to the beginning of the simulation and *t*_1_ is its end. The : denotes the double-dot product between second-order deviatoric stress tensors from the forward, $\boldsymbol{\tau }$, and adjoint, $\boldsymbol{\tau }^{\dagger }$, simulations. Although evaluation of eq. (4) is straightforward, it can become cumbersome in practice because the deviatoric stress at each time step of the forward simulation must be saved and therefore requires significant memory or disk space.

In a similar manner, we can obtain the initial sea-level Fréchet derivative by perturbing the *Lagrangian* (eq. A15) with respect to *SL*_0_, yielding


(5) \begin{eqnarray*}
K_{SL_{0}} = \rho _wgSL_{0}^{\dagger }\left(t^{\dagger }_1\right),
\end{eqnarray*}

where *ρ*_w_ is the density of water, *g* is the magnitude of gravitational acceleration and $SL_{0}^{\dagger }(t^{\dagger }_1)$ is the adjoint sea level at the final time step, $t^{\dagger }_1$, of the adjoint simulation. Note that this time is equivalent to the initial time, *t*_0_ of the forward simulation and that both Fréchet derivative eqs (4) and (5) are equivalent to those determined by Al-Attar & Tromp (2013) and Crawford *et al.* (2018).

Thus far, we have referred to K_*_ as the Fréchet derivative, where * indicates an arbitrary model parameter. More commonly, however, when *F* is an observation, then K_*_ is called the *sensitivity kernel*, and when *F* is a misfit function, then K_*_ is termed the *gradient* or more formally the gradient of the misfit function with respect to the model parameter. We adopt this nomenclature throughout the remainder of this study, but fallback on *Fréchet derivative* when the nature of *F* is ambiguous.

Finally, the units of the Fréchet derivative, K_*_, directly depend on the units of the functional, *F*, and they can be most easily obtained by examining the expression for the first-order change in the functional. To illustrate this aspect, let us allow *F* to be a sea-level observation in units of meters and consider the viscosity *sensitivity kernel, K*_ln _*_η_*. By inspection of eq. (2), we see that the units of this sensitivity kernel must be m^−2^. Similarly, if *F* is a *L*2 misfit function with units of *m*^2^ and we now consider the *gradient* of the misfit function with respect to the initial sea level (eq. 5), then by inspection of the surface integral in eq. (3), we see that the units of the *gradient* are m^−1^. These two examples are exactly the units of the viscosity sensitivity kernels discussed in Sections 6.3 and 6.4 and the *gradient* used in the iterative inversion for initial sea level discussed in Sections 4 and 6.2. However, these units are not easily obtained by inspection of eqs (4) and (5) because adjoint variables need not have the same units as their forward variable counterparts (e.g. **τ** and **τ**^†^). Instead the adjoint variable units depend on those of the adjoint loads (Section 3.1 and Appendix A2) and ultimately on those of the functional, *F*.

## 3 FURTHER DEVELOPMENT OF THE RATE FORMULATION OF THE FORWARD AND ADJOINT GIA PROBLEM

The adjoint method has previously been used to calculate viscosity sensitivity kernels for sea-level observations assuming a 1-D radial viscosity structure (Crawford *et al.*^2018^). In that study, only observations of sea level at a given location and instant in time were considered and, for clarity, we refer to these as *absolute sea-level* observations. Here we make two developments. First, the derivation of the adjoint loads required by a fundamental observation of palaeo sea level (i.e. relative sea level; Section 3.1). These observations are always made and reported relative to present-day sea level (e.g. Khan *et al.*^2019^), and, rather than existing in an absolute reference frame, are a measure of the change in sea level between the time of the sea-level indicator’s emplacement, *t*_obs_, and the present day, *t_p_*. We note that relative sea-level observations serve as the building blocks for related palaeo sea-level observations including the rate of sea-level change, the timing of sea-level highstands or transgressions (e.g. Nakada & Lambeck 1989), as well as relative sea-level curves and spatiotemporal fields (e.g. Creel *et al.*^2022^). Although understanding how these more complex observations sense Earth’s viscosity structure is important, we focus here only on the more fundamental observations of absolute and relative sea level, their relationship, and the influence of 3-D viscosity structure on sensitivity kernels for relative sea-level observations. This leads to our second development, which is the inclusion of lateral viscosity heterogeneity in the forward and adjoint GIA simulations (Section 3.2).

### 3.1 Adjoint loads for sea-level observations

Thus far, we have not directly addressed the form of the *fictitious* adjoint loads that drive the adjoint GIA simulations and allow us to calculate sensitivity kernels for observations related to the solid Earth, gravity, and sea level. The adjoint loads associated with these observations, as demonstrated in Appendix A2, are obtained by taking the first order perturbation of the scalar-valued function, *F*(**u**, *ϕ, SL*), with respect to the forward variables and can be schematically written as


(6) \begin{eqnarray*}
\delta F = \int ^{t_1}_{t_0} \int _{\partial M} \left( \dot{\mathbf {h}}_{\mathbf {u}} \cdot \delta \mathbf {u} + \dot{h}_{\phi } \delta \phi + \dot{h}_{SL} \delta SL\right) \, \mathrm{d}S \, \mathrm{d}t,
\end{eqnarray*}

where $\dot{\mathbf {h}}_{\mathbf {u}}$, $\dot{h}_{\phi }$ and $\dot{h}_{SL}$ are the Fréchet derivative of *F* with respect to solid Earth displacement (**u**), gravitational potential perturbation (*ϕ*) and sea level (*SL*), respectively. This sum is then integrated over the surface, ∂*M*, and over the duration of the simulation from *t*_0_ to *t*_1_. Note that these Fréchet derivatives are defined to be the time-derivative of some underlying functions, **h**_**u**_, *h*_*ϕ*_ and *h*_SL_. This formulation is chosen to maximize the symmetry between the forward and adjoint problems. We now derive the adjoint loads for observations of absolute sea level and relative sea level. Although the former is presented by Crawford *et al.* (2018), we begin by rederiving it here in order to demonstrate how these two types of observations are related, but also how they differ in the information that they convey.

Following Crawford *et al.* (2018), as well as our generalized discussion of the adjoint method (Appendix A2), we can determine the adjoint loads by schematically perturbing the scalar-valued functional *F* with respect to the state variables *U*. For an observation of absolute sea level at a given location and time, *SL*(**x**_obs_, *t*_obs_), this leads to


(7) \begin{eqnarray*}
\delta F = \int _{t_0}^{t_1} \int _{\partial M} \delta SL(\mathbf {x},t) \delta (\mathbf {x}-\mathbf {x}_{\rm obs}) \delta (t-t_{\rm obs}) \, \mathrm{d}S \, \mathrm{d}t
\end{eqnarray*}

where δ(**x** − **x**_obs_) and δ(*t* − *t*_obs_) are Dirac delta functions centred at the observation site, **x**_obs_, and time, *t*_obs_. From this equation, we see that the necessary functions defining the Fréchet derivatives are


(8) \begin{eqnarray*}
\dot{\mathbf {h}}_{\mathbf {u}} &=& \mathbf {0}, \\ \dot{h}_{\phi } &=& 0, \\ \dot{h}_{SL} &=& \delta (\mathbf {x}-\mathbf {x}_{\rm obs}) \delta (t-t_{\rm obs}),
\end{eqnarray*}

which are the values required by eqs (A16) and (A17) for an absolute sea-level observation at a given point in space and time.

We now undertake a similar procedure, but begin with the definition of relative sea level


(9) \begin{eqnarray*}
RSL(\mathbf {x}_{\rm obs},t_{\rm obs}) = SL(\mathbf {x}_{\rm obs},t_{\rm obs}) - SL(\mathbf {x}_{\rm obs},t_p),
\end{eqnarray*}

where *t_p_* is the present-day time, which is synonymous with *t*_1_ in our study. Again, we perturb *F* with respect to the state variables *U*, which for a relative sea-level observation results in


(10) \begin{eqnarray*}
\delta F = \int _{t_0}^{t_1} \int _{\partial M} \delta SL(\mathbf {x},t) \bigl[ \delta (\mathbf {x}&-&\mathbf {x}_{\rm obs}) \delta (t-t_{\rm obs}) \\ &&- \delta (\mathbf {x}-\mathbf {x}_{\rm obs}) \delta (t-t_{p}) \bigr] \, \mathrm{d}S \, \mathrm{d}t
\end{eqnarray*}

From this equation, it readily follows that the necessary functions are now


(11) \begin{eqnarray*}
\dot{\mathbf {h}}_{\mathbf {u}} &=& \mathbf {0}, \\ \dot{h}_{\phi } &=& 0, \\ \dot{h}_{SL} &=& \delta (\mathbf {x}-\mathbf {x}_{\rm obs})\delta (t-t_{\rm obs}) - \delta (\mathbf {x}-\mathbf {x}_{\rm obs})\delta (t-t_{p}).
\end{eqnarray*}

Examining eq. (11), we see that it is composed of two *fictitious* loads of equal magnitude and opposite sign that are applied at times *t*_obs_ and *t_p_*. By comparing it with eq. (8), we see that it fundamentally consists of two absolute sea-level adjoint loads. Therefore, the sensitivity kernels for relative sea-level observations can be obtained in one of two ways: (1) by using both adjoint loads in a single adjoint simulation or (2) by using each adjoint load in an independent adjoint simulation and then taking the difference of the resulting absolute sea-level sensitivity kernels [i.e. *K*_SL_(**x**_obs_, *t*_obs_) − *K*_SL_(**x**_obs_, *t_p_*)]. This property of superposition is routinely exploited in seismic tomography and will be utilized in our companion paper to image 3-D viscosity using palaeo sea-level observations.

### 3.2 Numerical implementation of 3-D viscosity

The introduction of lateral viscosity heterogeneity adds some complexity to solving the forward and adjoint GIA equations using a pseudo-spectral method, which was previously identified and solved by Martinec (2000). This complexity occurs in the first integral term on the right-hand side of eqs (A2) and (A17), which describes the viscous response of the system. A brief review of the numerical implementation of these equations as described by Crawford *et al.* (2018) is provided in Appendix B and we will invoke aspects of this review in what follows.

Our implementation of lateral viscosity heterogeneity within the forward and adjoint GIA simulations is discussed in Crawford (2019) and in essence follows Martinec (2000). Here, we focus on the viscous response as it appears in the reduced weak form of the forward GIA problem, eq. (A2), but note that a similar integral term also appears in the adjoint GIA problem, eq. (A17). These integral terms are identical up to the exchange of the forward and adjoint variables ($\left\lbrace \mathbf {m}, \mathbf {d}\right\rbrace \, \leftrightarrow \, \left\lbrace \mathbf {m}^{\dagger }, \mathbf {d}^{\dagger } \right\rbrace$; defined in Table A1), and thus, are evaluated in the same manner. When adopting a 1-D radial viscosity structure, we are required to evaluate


(12) \begin{eqnarray*}
&&\int _{0}^{R_\oplus } \int _{\partial M_r} 2\mu (r) \left[ \frac{1}{\tau (r)} (\mathbf {d}-\mathbf {m}) :(\mathbf {d}^{\prime }-\mathbf {m}^{\prime }) \right] \, \mathrm{d}S \, \mathrm{d}r = \\ && \int _{0}^{R_\oplus } \frac{2\mu (r)}{\tau (r)} \int _{\partial M_r} (\mathbf {d}-\mathbf {m}) :\mathbf {d}^{\prime } \, \mathrm{d}S \, \mathrm{d}r ,
\end{eqnarray*}

where the shear modulus, *μ*(*r*), and the *Maxwell relaxation time, τ*^−1^(*r*), being functions of only radius, *r*, are brought outside of the inner integral. Thus, the remaining terms within the inner angular integral can be expanded using generalized spherical harmonics and evaluated using the appropriate orthogonality relations.

In contrast, if viscosity varies laterally, we have to consider the more complicated expression


(13) \begin{eqnarray*}
&&\int _{0}^{R_\oplus } \int _{\partial M_r} 2\mu (r) \left[ \frac{1}{\tau (r,\theta ,\varphi )} (\mathbf {d}-\mathbf {m}) :(\mathbf {d}^{\prime }-\mathbf {m}^{\prime }) \right] \, \mathrm{d}S \, \mathrm{d}r
= \\ && \int _{0}^{R_\oplus } 2\mu (r) \int _{\partial M_r} \frac{1}{\tau (r,\theta ,\varphi )} (\mathbf {d}-\mathbf {m}) :\mathbf {d}^{\prime } \, \mathrm{d}S \, \mathrm{d}r.
\end{eqnarray*}

Since our numerical implementation only permits a 1-D radial elastic and density structure, the shear modulus, *μ*(*r*), must remain only a function of *r*. This assumption is reasonable since 3-D elastic effects are generally small and with the largest deviations occurring in regions of large load changes (Mitrovica *et al.*^2011^; Durkin *et al.*^2019^). Thus, in order to accommodate lateral variations in viscosity the *Maxwell relaxation time, τ*(*r, θ, φ*), now has an angular dependence indicated by {*θ, φ*}. To evaluate this integral term, we use a pseudo-spectral approach (e.g. Fornberg 1998; Kendall *et al.*^2005^) that performs certain operations in the spatial domain (e.g. multiplication) and other operations in the spectral domain (e.g. integration), while fast transformations are used to pass fields between these two domains.

A consequence of eq. (13) and the lateral heterogeneity of the *Maxwell relaxation time*, and hence viscosity, is that the spheroidal components of the displacement no longer decouple from the toroidal ones. This is because lateral variations in the Maxwell time generate toroidal components within the viscoelastic relaxation force applied at each time step. This situation is somewhat analogous to the toroidal–poloidal coupling (note poloidal and spheroidal are synonyms) that occurs within mantle in response to aspherical Earth structure (e.g. Forte & Peltier 1987). We note, however, that even in a laterally homogeneous Earth, the adjoint GIA problem can also excite toroidal displacement through the presence of tangential surface tractions in the adjoint load, which for example occurs for observations of horizontal solid Earth deformation. Thus, the reduced weak form of both the forward and adjoint GIA problems (eqs A2 and A17) may be schematically written for each spherical harmonic degree-*l* as two coupled sets of linear equations


(14) \begin{eqnarray*}
\mathbf {A}_l^s\dot{\mathbf {x}}_{lm}^s + \mathbf {g}_{lm}(\dot{\mathbf {x}}) = \mathbf {b}_{lm}^s,
\end{eqnarray*}

and


(15) \begin{eqnarray*}
\mathbf {A}_l^t\dot{\mathbf {x}}_{lm}^t = \mathbf {b}_{lm}^t,
\end{eqnarray*}

where the superscripts *s* and *t* denote the spheroidal and toroidal subsystems, respectively. Again focusing on the reduced weak form of the forward GIA problem, eq. (A2), the matrices $\mathbf {A}_l^{*}$ are constructed from its first term, $\mathcal {A}$, which is the Bilinear form associated with the elasto-gravitational forces (Al-Attar & Tromp 2013; Crawford *et al.*^2018^). The vector $\dot{\mathbf {x}}_{lm}^s$ contains the unknown spheroidal components, $\left\lbrace \dot{U}_{lm}, \dot{V}_{lm}, \dot{\phi }_{lm} \right\rbrace$, while the unknown toroidal component, $\dot{W}_{lm}$, is contained within the vector $\dot{\mathbf {x}}_{lm}^t$. Next, the vectors $\mathbf {b}_{lm}^{*}$ contain the integral terms on the right-hand side of eq. (A2) and contain the memory of the system and the forcing due to the ice-load change, all of which are known or readily calculated. Finally, the vector, $\mathbf {g}_{lm}(\dot{\mathbf {x}})$, originates from the second integral term of eq. (A2) that describes the radial forcing of the ocean and hence only arises in the spheroidal subsystem. As discussed in Appendix B, we solve eq. (14) iteratively and eq. (15) directly. In turn, solutions to these systems of equations can be mapped back into the more familiar spherical coordinate system [see appendix B of Crawford *et al.* (2018) or appendix C of Dahlen & Tromp (1999)]. Thus, by adopting these changes, we can now solve the forward and adjoint GIA problem subject to either a 3-D viscosity structure, surface tractions or both simultaneously at any given instant in time.

## 4 RECALIBRATION OF INITIAL SEA LEVEL USING GRADIENT-BASED OPTIMIZATION

Predictions of past or future sea level and topography, regardless of the adopted Earth structure and ice history, should result in realistic topography that matches the observed present-day topography. This initial value problem is well-known within the GIA community and is commonly addressed by iteratively updating the prescribed initial sea level by subtracting the difference between the predicted and observed present-day sea level until the desired level of accuracy is achieved (e.g. Kendall *et al.*^2005^). Here, we take a different approach that uses the adjoint method in combination with gradient-based optimization (as suggested by Crawford *et al.*^2018^). We will find this approach particularly useful in future work that simultaneously updates multiple model parameters (e.g. mantle viscosity and initial sea level; Lloyd *et al.* in preparation). For now, we focus on the basics of recalibrating the initial sea level for any set of Earth and ice history models.

In our approach, each iteration, *i*, begins with a forward GIA simulation that is initiated, in part, by the current estimate of initial sea level and is followed by calculating the misfit at the present day, *t_p_*, according to the function


(16) \begin{eqnarray*}
\mathcal {J}^i = \frac{1}{2} \int _{\partial M} \left[ SL_{\rm prd}^i(\mathbf {x},t_p) - SL_{\rm obs}(\mathbf {x},t_p) \right]^{2} \, \mathrm{d}S.
\end{eqnarray*}

Here, *SL*_prd_(**x**, *t_p_*) and *SL*_obs_(**x**, *t_p_*) are the present-day predicted and observed sea level, respectively, at position **x** ∈ ∂*M*. We next calculate the adjoint loads in the same manner as in Section 3.1, but now by perturbing eq. (16) with respect to *SL*_prd_, yielding


(17) \begin{eqnarray*}
\dot{h}_{SL} = \left[SL^i_{\rm prd}(\mathbf {x},t_p) - SL_{\rm obs}(\mathbf {x},t_p) \right] \delta (t-t_p),
\end{eqnarray*}

where again, $\dot{h}_{\mathbf {u}}$ and $\dot{h}_{\phi }$ are zero. We see that the sea-level adjoint load described by eq. (17) is nearly identical to that of eq. (8), with the exception that it may have non-zero values globally and is weighted by the difference between the predicted and observed present-day sea level. It is this weighted adjoint load that drives the adjoint GIA simulation in the initial sea-level recalibration and, due to these weights, we now obtain the gradient of the misfit function with respect to the initial sea-level, $D_{SL_0}\mathcal {J}^i$, through eq. (5). Note that eq. (5) depends on the adjoint sea level at the final adjoint time, $t^{\dagger }_{1}$, or equivalently at the the initial time, *t*_0_, of the forward GIA simulation. Thus, for each iteration we must complete the full viscoelastic GIA simulation. This formulation is consistent with the equations of Crawford *et al.* (2018), but not their manuscript text where, due to a typographic error, it is stated that only the elastic adjoint problem needs to be solved. In the calculations of this study, we use the correct expression as revised above.

With the gradient in hand, we can determine the search direction and step length necessary to find a new initial sea level that minimizes the misfit function of eq. (16). Empirically, we have determined that greater misfit reduction and a better overall match to the present-day sea level can be obtained through a strategy that starts with a low-pass filter of the gradient before retaining higher-degree information in later iterations. Here, filtering is performed in the spherical harmonic domain by applying a one-sided Hanning taper as a function of degree-*l*, which has weights of


(18) \begin{eqnarray*}
w(l) = \left\lbrace \begin{array}{@{}l@{\quad }l@{}}1, & 0 \le l\lt l_c\\
\frac{1}{2} \left[1 - \cos \left(\pi \frac{l_{max}-l}{l_{max}-l_c} \right) \right], & l_c \le l \le l_{max}\\
0, & \text{otherwise} \end{array}\right.,
\end{eqnarray*}

where *l*_max_ is the maximum spherical harmonic degree and *l*_c_ is the cut-off degree (i.e. corner frequency). In the example of Section 6.2, *l*_max_ is 64 and we set *l*_c_ to 60 when smoothing is applied to the gradient. We will discuss these choices further in that section. For now, we need only distinguish the smoothed or, in more general terms, preconditioned gradient as $P D_{SL_0}\mathcal {J}^i$, where *P* is an arbitrary preconditioning operator.

The gradient, $D_{SL_0}\mathcal {J}^i$, and preconditioned gradient, $P D_{SL_0}\mathcal {J}^i$, are used to determine the search direction using the method of steepest descent (Cauchy 1847). We have also explored using the conjugate gradient method instead (Polak & Ribiere 1969), but leave discussion of this algorithm to the companion paper. In the steepest descent method, the search direction, ψ^*i*^, is equal to the negative of the preconditioned gradient. Thus, updates to the initial sea level, *SL*_0_, can be obtained using


(19) \begin{eqnarray*}
SL_{0}^{i+1} = SL_{0}^i + \alpha \psi ^i,
\end{eqnarray*}

where *α* is the step length, for which we seek the optimum value that minimizes the misfit in eq. (16).

We determine the optimal step length for α by assuming that the misfit along the projection of the search direction forms a parabola, similar to the approach used by Tape *et al.* (2007) for seismic tomography. Given that at *α* = 0, we already have the misfit, $\mathcal {J}^i$, and can readily obtain the slope of this parabola by calculating the directional derivative along the search direction (i.e. $\left\langle D_{SL_0}\mathcal {J}^i,\psi ^i \right\rangle$), it only remains to determine the misfit for a trial step length. Here, this length is taken to be twice the *x*-intercept of the line described by the misfit and slope at *α* = 0, which is


(20) \begin{eqnarray*}
\alpha _t = -2 \frac{\mathcal {J}^i}{\left\langle D_{SL_0}\mathcal {J}^i,\psi ^i \right\rangle }.
\end{eqnarray*}

The resulting initial sea-level, $SL_{0}^i+\alpha _t\psi ^i$, is then used to perform another forward GIA simulation and we again calculate the misfit, $\mathcal {J}_{\alpha _t}^i$. With these pieces of information, we can now determine a unique quadratic curve and its minimum value


(21) \begin{eqnarray*}
\alpha = \frac{\left\langle D_{SL_0}\mathcal {J}^i,\psi ^i \right\rangle \alpha _{t}^2}{2 \left( \mathcal {J}^i - \mathcal {J}_{\alpha _t}^i + \left\langle D_{SL_0}\mathcal {J}^i,\psi ^i \right\rangle \alpha _t \right)},
\end{eqnarray*}

which is a suitable step length that can be used to obtain a revised estimate for the initial sea level, $SL_{0}^{i+1}$, using eq. (19). In the above procedure, it is critical to distinguish between the gradient, $D_{SL_0}\mathcal {J}^i$, and preconditioned gradient, $P D_{SL_0}\mathcal {J}^i$, since a failure to do so may cause the parabolic assumption to break down and result in an ineffective estimate of the optimal step length. The degree to which this occurs depends on the extent that the gradient is modified by preconditioning. Finally, this procedure is iteratively repeated until the convergence criteria is met. For recalibration of initial sea level, we choose the convergence criteria to be $\vert SL^i_{\rm prd}(\mathbf {x},t_p) - SL_{\rm obs}(\mathbf {x},t_p) \vert \lt 0.5$ m ∀**x** ∈ ∂*M* (i.e. the total difference between the predicted and observed present day sea level is less than 50 cm), based on the GIA benchmark study of Martinec *et al.* (2018).

## 5 FORWARD AND ADJOINT GIA SIMULATION SETUP

**Figure 1.**
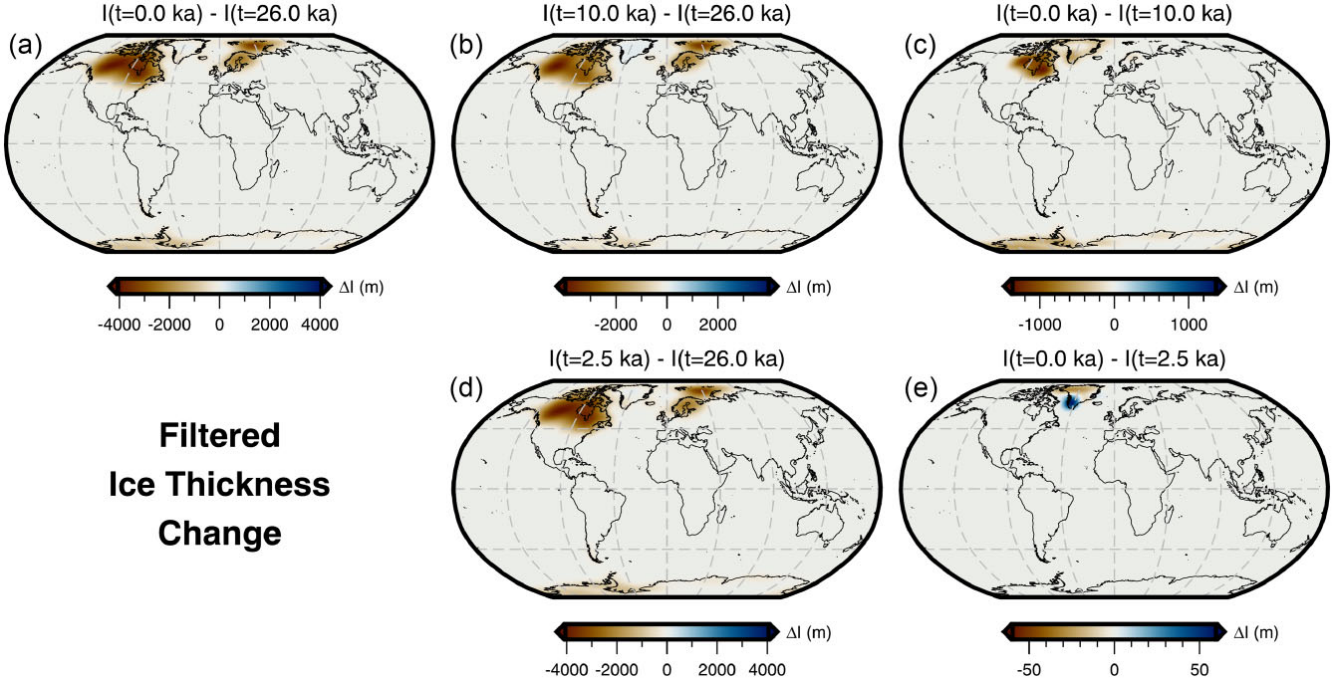
Ice thickness changes. Maps of low-pass filtered ice-thickness change based on the ICE6G(VM5a) ice-history model (Argus *et al.*^2014^; Peltier *et al.*^2015^) between (a) 26–0 ka, (b) 26–10 ka, (c) 10–0 ka, (d) 26–2.5 ka and (e) 2.5–0 ka. Panels (a), (b) and (c) are most appropriate for understanding the load changes associated with absolute sea-level observations at 10 and 0 ka and the relative sea-level spanning 10–0 ka (Sections 6.3 and 6.4). Likewise, panels (a), (d) and (e) are most appropriate for understanding the load changes associated with absolute sea-level observations at 2.2 and 0 ka and the relative sea-level spanning 2.2–0 ka (Section 6.4).

**Figure 2.**
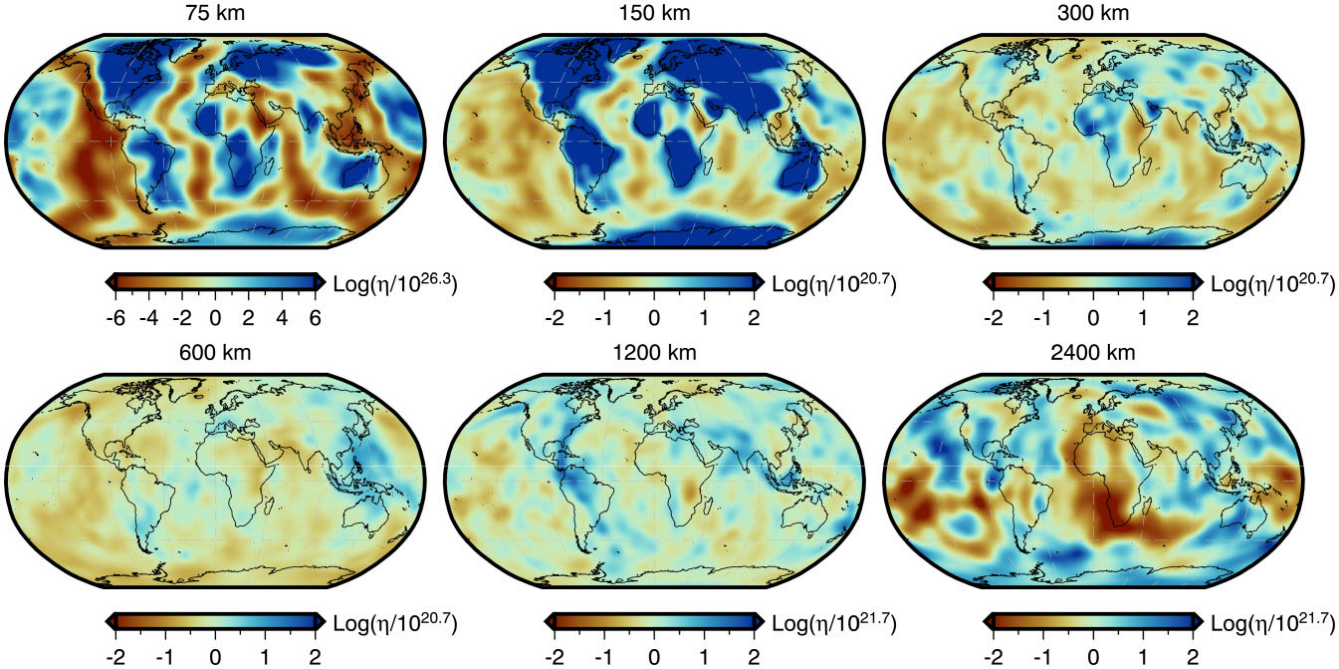
Filtered and bounded 3-D viscosity structure. Depth slices through the filtered and bounded version of our inferred 3-D viscosity model (the unmodified version is shown in Fig. S2). Viscosity anomalies at each depth are relative to the 1-D radial model described at the end of Section 5.1 and shown in Fig. S4. This 3-D model is used in the forward and adjoint simulations of Section 6.2 to determine a target present-day sea level for recalibration of the initial sea level. It is also used in Section 6.4 to explore the effect of 3-D structure on viscosity sensitivity kernels.

Throughout this study, we perform forward and adjoint GIA simulations at spherical harmonic degree 64 and for a duration of 26 kyr (i.e. 26 ka to 1950 CE) using a spatially filtered version of the ICE6G(VM5a) ice history model (Fig. 1; Argus *et al.*^2014^; Peltier *et al.*^2015^). The initial sea level (i.e. topography) at 26 ka is prescribed and is either $SL^{0}_{0}$ or $SL^{7}_{0}$, which are detailed in Sections 6.1 and 6.2. For the solid Earth structure, we use the 1-D elastic and density structure of PREM (Dziewonski & Anderson 1981) in combination with either a filtered and bounded 3-D viscosity structure (Fig. 2) or its 1-D radial representation (Section 5.1; Fig. S4). Our 3-D viscosity structure is based on the shear-wave speed model of GLAD-M25 (Bozdağ *et al.*^2016^; Lei *et al.*^2020^) and its creation will be discussed in detail in Section 5.1. Nevertheless, a couple of pertinent details are relevant to the setup of the forward and adjoint GIA simulations. For example, both viscosity models extend to Earth’s surface and thus, our simulations do not formally include an elastic lithosphere. Instead the extent of the high-viscosity regions in combination with the load change characteristics determines which regions will be dominated by elastic deformation. In this manner, simulations containing lateral viscosity variations also include effects due to lateral changes in lithospheric thickness.

Given the low resolution of the forward and adjoint GIA simulations in comparison to the resolution of the input fields (e.g. 3-D viscosity structure, surface topography and ice thickness), we spatially filter these data sets to avoid aliasing and minimize the Gibbs phenomenon due to truncation of the spherical harmonic series to degree 64. This low-pass filtering is achieved by applying a one-sided Hanning taper as a function of degree, *l* (eq. 18), to each of the fields in the spectral domain. Unlike our previous application of eq. (18), the cut-off degree *l*_c_ is set equal to 0, such that degree 0 (i.e. the spherical mean) is the only degree to retain its original amplitude.

As discussed in Appendix B, the simulations use an explicit time-stepping scheme and this time step is approximately one half of the smallest *Maxwell relaxation time*. Thus, for our 1-D viscosity structure, this time step is 50 yr, while for our unmodified 3-D viscosity inference (Fig. S2) the required time step would be approximately 0.05 yr. In the latter case, a single forward or adjoint simulation on a single compute node using OpenMP would take approximately two weeks and require more than a terabyte of memory to store the needed forward variables for the viscosity kernel calculation (eq. 4). This requirement exceeds our computational resources and, due to the exploratory nature of this study, we instead choose to limit the minimum viscosity to 2 × 10^19^ Pa·s. With this modification, the time step becomes 1 yr and the run time is ∼18 hr, however calculating the viscosity sensitivity kernels (eq. 4) remains memory intensive. Thus, we save the deviatoric stress tensor every 50 yr, which we find to be sufficient when numerically integrating eq. (4) using the rectangle rule.

### 5.1 An inference of 3-D mantle viscosity from GLAD-M25

We construct a new inference of 3-D mantle viscosity based on a similar approach to Austermann *et al.* (2021) and using the Voigt average shear-wave speeds of GLAD-M25 (Fig. 1; Bozdağ *et al.*^2016^; Lei *et al.*^2020^). GLAD-M25 is the second generation of a global adjoint tomography model (Bozdağ *et al.*^2016^), whose starting point consists of the S362ANI seismic model of Kustowski *et al.* (2008) combined with crustal structure from CRUST2.0 (Bassin 2000). Its construction over the course of 25 iterations minimizes the phase misfit of three-component body and surface waves (periods of 17–25 and 40–250 s, respectively), as well as reflections and overtones, from 1480 earthquakes. This minimization is achieved using gradient-based optimization in combination with the adjoint method and the computational package SPECFEM3D_GLOBE (Komatitsch & Tromp 2002a, b), which allows for accurate and efficient calculation of both synthetic three-component seismograms and the gradient of the misfit function with respect to the model parameters. Although a formal assessment of the model’s resolution remains a challenge, point-spread function tests (Fichtner & Trampert 2011) as well as comparisons with other global and regional tomography models suggest that GLAD-M25 is slowly beginning to close the gap between global and regional studies in densely sampled areas (Lei *et al.*^2020^). Nevertheless, we acknowledge that the absence of surface waves at periods less than 40 s suggests that the uppermost mantle may be less well resolved than in other global upper mantle tomography models (e.g. Schaeffer & Lebedev 2013). We note, however, that no current tomography model has the required global coverage and the required resolution to capture the shallow fine-scale structure that will be important for GIA modelling.

Our inference of 3-D mantle viscosity consists of three components: (1) an inverse calibration scheme for the upper mantle (Richards *et al.*^2020^), (2) a traditional inference for the transition zone and lower mantle (Austermann *et al.*^2021^) and (3) a merging of the two domains, which includes near-surface corrections and additional rheological constraints. In all instances, we relate shear-wave speed and attenuation to steady-state diffusion creep viscosity or viscosity perturbations by way of temperature. In so doing, we account for both linear anharmonic (Kumazawa & Anderson 1969) and non-linear anelastic (Cammarano *et al.*^2003^; Karato 1993) effects, with the latter being more pronounced in warm regions where temperatures approach the solidus. Failure to account for anelasticity can lead to overestimates of absolute mantle temperatures and, by extension, underestimates of absolute mantle viscosity by an order of magnitude (Austermann *et al.*^2021^). Furthermore, we assume that shear-wave speed variations relative to a reference model are due to temperature alone. Although this assumption is incorrect, it is common (e.g. Cammarano *et al.*^2003^; Priestley & McKenzie 2006, 2013; Richards *et al.*^2020^) and perhaps reasonable to assume that temperature effects dominate at global scales given uncertainties in material properties of the mantle (e.g. composition, grain size, and melt fraction; Schutt & Lesher 2006; Connolly & Khan 2016; Dannberg *et al.*^2017^; Debayle *et al.*^2020^)) and the rheological mechanisms controlling anelasticity (Jackson & Faul 2010; Yamauchi & Takei 2016). Equally important uncertainties arise from the tomographic models, whose imaged wave speeds are influenced by the inverse problem setup (e.g. choice of parametrization, regularization, and simplifying assumptions), the seismic phases of interest and their sensitivity to Earth structure, as well as the spatial and temporal distribution of sources (e.g. noise, earthquakes, etc.) and seismic stations. To manage and minimize these uncertainties, at least for the upper mantle, we use the approach of Richards *et al.* (2020).

The inverse calibration scheme of Richards *et al.* (2020) is rooted in the experimentally derived anelastic parametrization of Yamauchi & Takei (2016), which includes the effect of pre-melting (Takei *et al.*^2014^). It also follows the methodological philosophy of Priestley & McKenzie (2013) that any mapping of one mantle parameter to another should satisfy a range of average mantle properties for which there exists independent constraints. Thus, given a suite of experimentally determined parameters (Table 1) that capture the dependence of anelasticity on frequency, depth and homologous temperature, we can determine a set of globally averaged mantle material properties that satisfy existing independent constraints. An important advantage of this calibration procedure is that it ensures the non-linear decrease in shear-wave speeds and attenuation near the solidus are faithfully reproduced, regardless of the assumed relative contribution of temperature, composition, grain size and melt fraction to the observed seismic parameters. Since the non-linear behaviour is ultimately controlled by the diffusion creep viscosity and directly constrained by the seismological observations, our steady-state viscosity estimates are remarkably robust to uncertainty in these thermodynamic variables (see Text S1 in Hazzard *et al.*^2023^).

**Table 1.** Experimentally determined anelasticity parameters (left) from Yamauchi & Takei (2016) and the globally averaged mantle material properties (right) determined by the inverse calibration scheme of Richards *et al.* (2020).

Experimentally determined parameters		Globally averaged mantle material properties	
Variable	Value	Variable	Value
*A_B_*	0.664	*μ*_0_	80.82 GPa
*α_B_*	0.38	$\frac{\partial \mu }{\partial T}$	−0.02 GPa °C^−1^
$\tau ^{\prime }_P$	6 × 10^−5^	$\frac{\partial \mu }{\partial P}$	2.292
*β*(*φ*)	∼0	log_10_*η_r_*	23.301 [log_10_ (Pa·s)]
Δ_poro_(*φ*)	∼0	*E_a_*	545 kJ mol^−1^
*γ*	5	*V_a_*	9.633 × 10^−7^ m^3^ mol^−1^
$T^{\prime }_{\eta }$	0.94	$\frac{\partial T_s}{\partial z}$	0.8634 °C km^−1^
*λφ*	∼0		

In our mapping, as in Richards *et al.* (2020), we make use of four independent constraints and evaluate each with an L2 misfit function that is weighted by uncertainties and is appropriately normalized by the sample size. The observations consist of shear-wave speeds from oceanic regions of GLAD-M25 that are stacked with respect to lithospheric age and depth relative to sea level, as well as inferences of mantle properties (temperature, attenuation and bulk viscosity). Sampling of these observations is performed in an identical manner to Richards *et al.* (2020) unless otherwise stated. The first constraint compares the oceanic stack of shear-wave speed to those predicted by the plate-cooling model of Richards *et al.* (2018), in which we assume an ambient potential temperature of 1333 °C and an equilibrium plate thickness of 133 km. Secondly, we require the inferred temperature between 225 and 400 km depth beneath oceanic regions to be isentropic on average (i.e. both adiabatic and reversible) and to follow the 1333 °C isentrope (Shorttle *et al.*^2014^). Thirdly, the inferred average attenuation structure obtained from the relationships of Yamauchi & Takei (2016) must converge to the 1-D attenuation structure of QL6 (Durek & Ekström 1996), the same profile used in the construction of GLAD-M25, beneath old oceanic lithosphere. Finally, we require that the average of the inferred steady-state diffusion creep viscosity between 225 and 400 km depth be approximately 3 × 10^20^ Pa·s (Lau *et al.*^2016^). These four misfit functions are subsequently combined using weighting factors of 10, 1, 2 and 2, respectively, in order to calculate total misfit.

To determine the optimal set of globally averaged mantle material properties that satisfy the above constraints, we initially perform a coarse parameter sweep in order to bound the global minimum. The parameter set with the lowest misfit value is then chosen as the starting point in a conjugate gradient scheme (Powell 1964; Press *et al.*^1986^) that seeks to further converge on the global minimum. The resulting parameters can be found in Table 1 and are used to convert upper mantle shear-wave speeds of GLAD-M25 into temperature and absolute steady-state diffusion creep viscosity down to 400 km depth.

At greater depths, we lack sufficient observational constraints to apply the inverse calibration scheme of Richards *et al.* (2020) and must fall back on more traditional approaches. Here, we follow Austermann *et al.* (2021) and convert shear-wave speed variations relative to the 1-D radial average of GLAD-M25 into temperature variations about a quasi-steady state mantle geotherm (Schuberth *et al.*^2009^). The anharmonic component of this conversion assumes a pyrolitic mantle composition and makes use of the Perple_X Gibbs free-energy minimization software (Connolly 2005) along with the thermodynamic database of Stixrude & Lithgow-Bertelloni (2011). An anelastic correction is made based on the 1-D attenuation model Q5, associated relationships from Cammarano *et al.* (2003), and a mantle solidus from Andrault *et al.* (2011). Finally, these temperature variations are mapped to viscosity variations following Steinberger & Calderwood (2006).

We now merge these two domains in order to produce a spherical 3-D viscosity model of the mantle and crust that has a high-viscosity lid, an average viscosity of 5 × 10^20^ Pa·s in the sublithospheric upper mantle, and an average viscosity of 5 × 10^21^ Pa·s in the lower mantle. In doing so, we address the fact that GLAD-M25’s topology geometrically includes ellipticity, surface topography and internal seismic discontinuities (e.g. the Moho; Bozdağ *et al.*^2016^; Lei *et al.*^2020^), as well as the fact that updates to the model may cause crust or mantle wave speeds to exceed the extent of the *a priori* prescribed and fixed Moho. To determine crustal viscosities we first identify the extent of a a *crust-like* region. For the upper bound we ignore the topography and bathymetry present in GLAD-M25 and define the upper surface to coincide with present-day sea level. Meanwhile, the depth of the *crust-like* region is taken to be either the Moho prescribed by the starting model of GLAD-M25 (i.e. CRUST2.0; Bassin 2000) or the depth of the minimum temperature inferred by the inverse calibration scheme. Next, we identify the lithosphere–asthenosphere boundary (LAB) as the 1175 °C isotherm, similar to Austermann *et al.* (2021), and find that the spherical average depth of this boundary is ∼100 km. Furthermore, the volumetrically averaged viscosity of the mantle lithosphere is ∼1.5 × 10^26^ Pa·s and it is this value that we assign to the *crust-like* region. Thus, the volumetric average of the entire lithosphere remains unchanged, with constant viscosities within the crust and laterally variable ones within the lithospheric mantle.

At 400 km depth, we transition from using the inverse calibration scheme of Richards *et al.* (2020) to the more traditional approach of Austermann *et al.* (2020), which does not involve a calibration. At this depth, we average the two viscosity inferences in logarithmic space assuming a reference viscosity of 5 × 10^20^ Pa·s (or ∼20.699 in logarithmic space) for the traditional approach. It is this reference viscosity that we enforce as the volumetric average of the sublithospheric upper mantle extending from the LAB down to 670 km depth, similar to Austermann *et al.* (2021). However, unlike in their 3-D viscosity inference, we impose this condition differently. We calculate the volumetric average viscosity of the sublithospheric upper mantle (∼20.914 in logarithmic space) and apply a uniform shift of −0.215 in log  space in order to satisfy this constraint. Finally, within the lower mantle (i.e. 670–2891 km depth), absolute viscosity is determined assuming a reference viscosity of 5 × 10^21^ Pa·s, which is also the adopted average viscosity of the lower mantle.

The resulting 3-D viscosity inference is shown in Fig. S2 and the entire model may be found in the Supporting Information. Likewise, estimates of LAB depth based on the 1175 °C isotherm are provided and shown in Fig. S3. This separation is done to avoid confusion with the filtered and bounded 3-D viscosity model (Fig. 2) that is derived from this initial inference and used in the forward and adjoint GIA simulations. Our treatment of the mantle and crust as entirely viscoelastic is a departure from traditional GIA models that invoke an elastic lid (which implies knowledge of the effective elastic thickness of the lithosphere). Constraining this thickness remains challenging and its meaning varies across geophysical disciplines (e.g. Lau *et al.*^2020^). Instead, we believe a more elegant approach is to avoid defining the elastic thickness and instead allow the degree of elastic versus viscous deformation to be determined by material properties interacting with the geometry and timescale of surface load changes.

As a final step, we construct a comparable 1-D radial viscosity model based on this 3-D viscosity inference (Fig. S4). This model consists of a 100-km-thick, high-viscosity (∼1.5 × 10^26^ Pa·s) lid, a sublithospheric upper mantle (100–670 km depth) viscosity of 5 × 10^20^ Pa·s, and a lower mantle (670–2891 km depth) viscosity of 5 × 10^21^ Pa·s. We use this model in all forward and adjoint GIA simulations herein that adopt a 1-D viscosity model.

## 6 RESULTS AND DISCUSSION

### 6.1 Forward simulations of sea-level change

**Figure 3.**
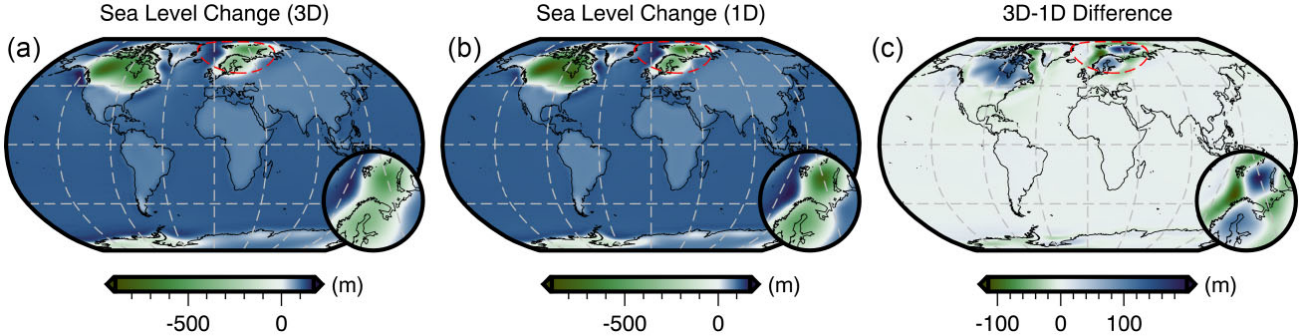
Predicted sea-level change from 26 ka to 1950 CE for different viscosity structures under the assumption of a fixed initial sea level. (a) Results that adopt the filtered and bounded 3-D viscosity structure (Fig. 2) or (b) the 1-D viscosity structure (Section 5.1). In these two maps blue colours indicate sea-level rise and green colours indicate sea-level fall. (c) The total sea-level change difference at 1950 CE between models that adopt the 3-D and 1-D viscosity structures. Here, blue (green) colours indicate greater (lesser) sea level relative to the 1-D simulation results. Finally, the red dashed line shows the location of the inset map.

The total sea-level change from 26 ka to 1950 CE predicted by forward GIA simulations and driven by the filtered ICE6G(VM5a) ice history model (Section 5) is shown in Fig. 3. This figure includes results that adopt both the filtered and bounded 3-D viscosity model (Fig. 2) and its 1-D radial representation (Section 5.1). As expected, the largest total sea-level change occurs near the former Laurentide and Fennoscandian ice sheets, in which peak sea-level fall reaches approximately −800 m over the course of 26 kyr for the 1-D viscosity model (Fig. 3b). In contrast, adoption of our 3-D viscosity model results in peak sea-level fall of approximately −700 m and approximately −500 m within the footprint of the Laurentide and Fennoscandian ice sheets, respectively (Fig. 3a). The difference in total sea-level change in these two simulations is shown in Fig. 3(c) and is equivalent to the difference in their final sea level since they use the same value of initial sea level [obtained from the filtered ICE6G(VM5a) ice history]. Thus, for later clarity, we refer to the results in Fig. 3(c) as the *difference in final sea level*.

In the near field, which includes the ice sheets and their forebulge, we observe higher sea level for the 3-D viscosity model within the footprint of the former Laurentide and Fennoscandian ice sheets, as well as within coastal regions of East Antarctica and Greenland (Fig. 3c). As expected, sea level is generally lower at the peripheries of these regions within the forebulge. In contrast, we find lower sea level within West Antarctica and central Greenland, and where a clear forebulge exists, higher sea level is observed. These differences in final sea level in part reflect the relative stiffness of our 3-D viscosity structure with respect to its 1-D radial representation. In our 3-D viscosity model, the Canadian Shield, Fennoscandian Shield, Greenland and East Antarctic Shield are all underlain by an overall stiffer mantle, which reflects their thick, cold, in some cases cratonic lithosphere and their long-term tectonic stability. As a result of stiffer mantle, these regions experience less solid Earth deformation in response to ice-mass change. Thus, areas of net ice-mass loss experience lower uplift and subsidence, leading to higher sea level within the footprint of the ice sheets and lower sea level within the forebulge. Within areas of net ice-mass gain (e.g. central Greenland) deformation is similarly muted, but the direction of deformation and by extension sea-level change is opposite. In contrast, the mantle underlying West Antarctica is weaker in our 3-D viscosity structure relative to its 1-D radial representation, which reflects the warmer mantle and thinner lithosphere that are characteristic of tectonically active regions. When these regions experience net ice mass loss greater solid Earth uplift (i.e. lower sea level) occurs directly beneath the load change, while greater solid Earth subsidence (i.e. higher sea level) is found at the peripheries. Finally, we note that a similar pattern is observed in Patagonia and reflects regional ice-mass loss and a weaker mantle, although this feature is of insufficient amplitude to be visible in Fig. 3(c).

In the far field (i.e. beyond the extent of forebulges), sea level is generally higher by up to 10 m in the open ocean for the 3-D viscosity model relative to its 1-D radial representation. As for the near field, final sea-level differences in the far field arise, in part, due to the difference in viscous structure and, by extension, lithospheric thickness between the two viscosity models. However, the strength of ocean siphoning and expulsion (i.e. sea floor subsidence and uplift, respectively) in the near field also modulates the far field sea level. Meanwhile, a more complex pattern with a similar magnitude is observed along coastal regions and often includes a switch in polarity across the coastline that reflects variations in the magnitude of continental levering. A detailed examination of the influence of 3-D structure on continental levering is beyond the scope of this work and we instead refer the reader to Austermann *et al.* (2021).

Although much of the difference in final sea level shown in Fig. 3(c) is due to the viscosity contrast between our 3-D viscosity model and its 1-D radial representation, a component is also due to our assumption that the initial sea level is the same for both simulations. As a result of their different viscoelastic properties, some regions, particularly marine-based sectors of the ice sheets, are subject to alternative histories of ocean loading and unloading, solid Earth deformation, and gravitational changes. Quantifying this contribution requires determination of an initial sea level for each individual simulation that will yield a consistent sea-level (i.e. topography) prediction at the final time step. Thus, we now turn our attention to recalibration of initial sea level.

### 6.2 An example of the initial sea-level recalibration

**Figure 4.**
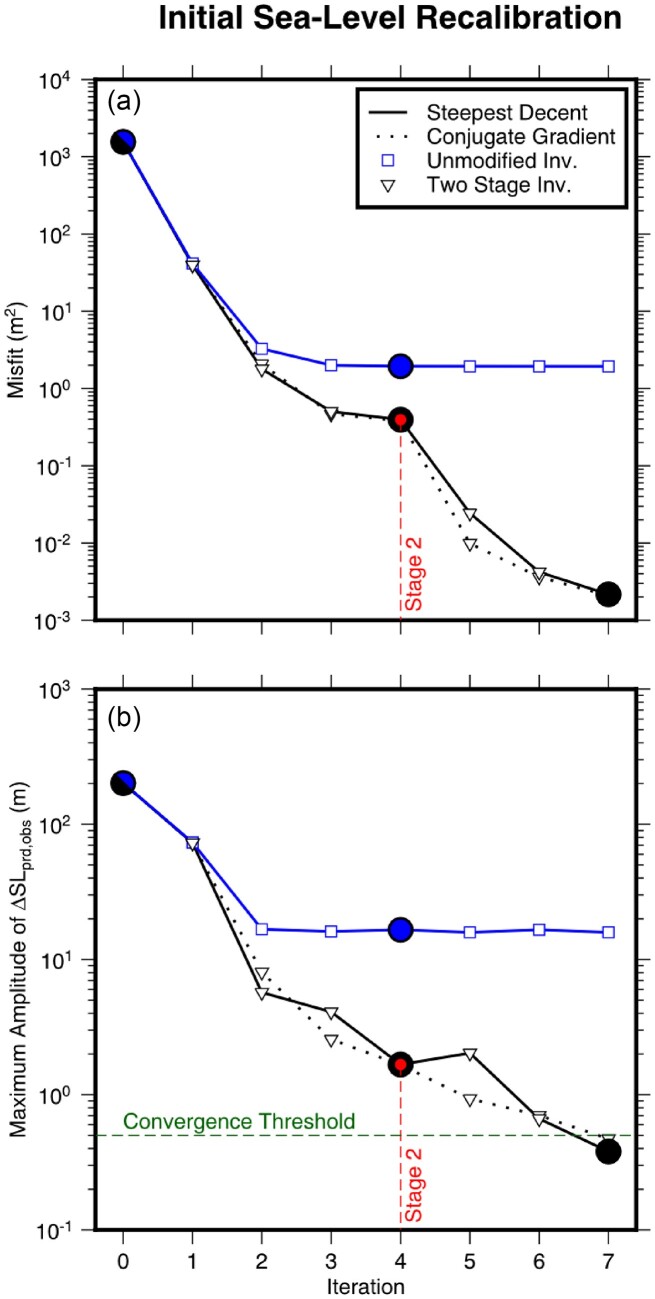
Evolution of the misfit and convergence of the initial sea-level recalibration. (a) A plot of misfit versus iteration number showing: (blue line) the unmodified recalibration procedure where no smoothing is applied to the gradient; and (black lines) two-stage recalibration procedure where the gradient is initially smoothed for four iteration (i.e. prior to the red line) using eq. (18) with *l*_c_ = 60, beyond which no smoothing is applied to the gradient. The solid and dashed lines indicate inversions whose search directions are determined by steepest decent or conjugate gradient, respectively. Finally, the large blue and black circles indicate iterations shown in Figs 5 and 6, respectively (b) A plot showing convergence, which is evaluated using the maximum amplitude of the difference between the observed and predicted final sea level. The green dashed line indicates the convergence threshold of 0.5 m and other annotations are similar to panel (a). In this study, we use the result from the two-stage procedure using steepest descent.

**Figure 5.**
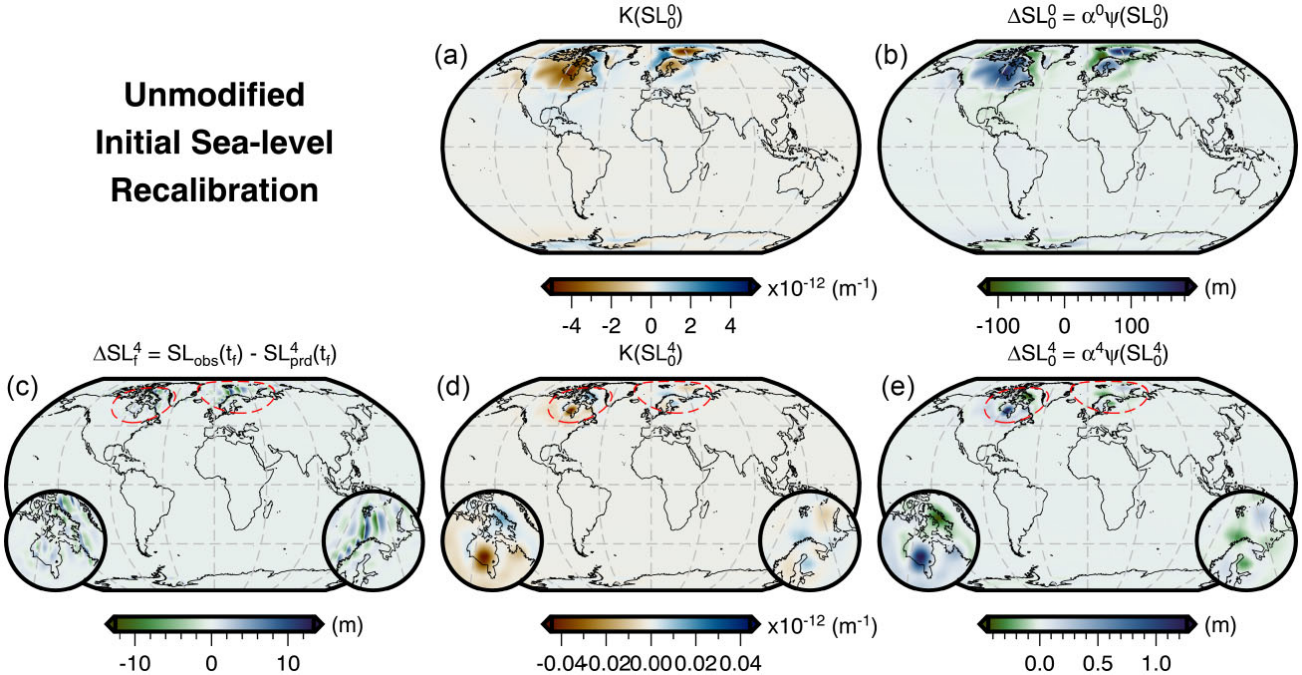
Unmodified initial sea-level recalibration. (a) The initial sea-level sensitivity kernel for the first iteration. (b) The update applied to the initial sea level in the first iteration. (c) The difference between the observed (i.e. target) present-day sea level and the prediction after four iterations (N.B., equivalent plot for the first iteration appears in Fig. 3c). (d) The initial sea-level sensitivity kernel for the fourth iteration. (e) The update applied to the initial sea level in the fourth iteration. The red dashed line shows the location of the 30°-wide inset maps over Canada and Fennoscandia plotted in lower left and right, respectively. These maps show the features that ultimately cause the inversion to fail to converge. The corresponding misfit and convergence evolution of this procedure are shown by the blue line in Fig. 4.

Following the procedure laid out in Section 4, we perform an initial sea-level recalibration using a synthetic example. We have chosen to adopt the final sea level predicted by the forward GIA simulation using the filtered and truncated 3-D viscosity model as the *observed* present-day sea level. We then iteratively invert for the initial sea level that is required to match this ‘observation’ for simulations that instead use the 1-D viscosity model. We find that this inversion converges rapidly over the course of 4–5 iterations, during which the greatest misfit reduction (≥90 per cent) occurs in the first iteration (Fig. 4a). Neglecting to implement a suitable smoothing strategy, however, leads the inversion to become easily trapped in local minima that are related to instabilities in the vicinity of the former marine ice sheets. For such an inversion without smoothing, this behaviour results in differences between predicted and observed final sea level of ±25 m to the north of Fennoscandia as well as ±10 m in Hudson Bay and the Northwestern Passages of North America (Fig. 5c). These instabilities dominate the highest degrees of our spherical harmonic basis functions and likely arise from their truncation above *l*_max_ = 64.

**Figure 6.**
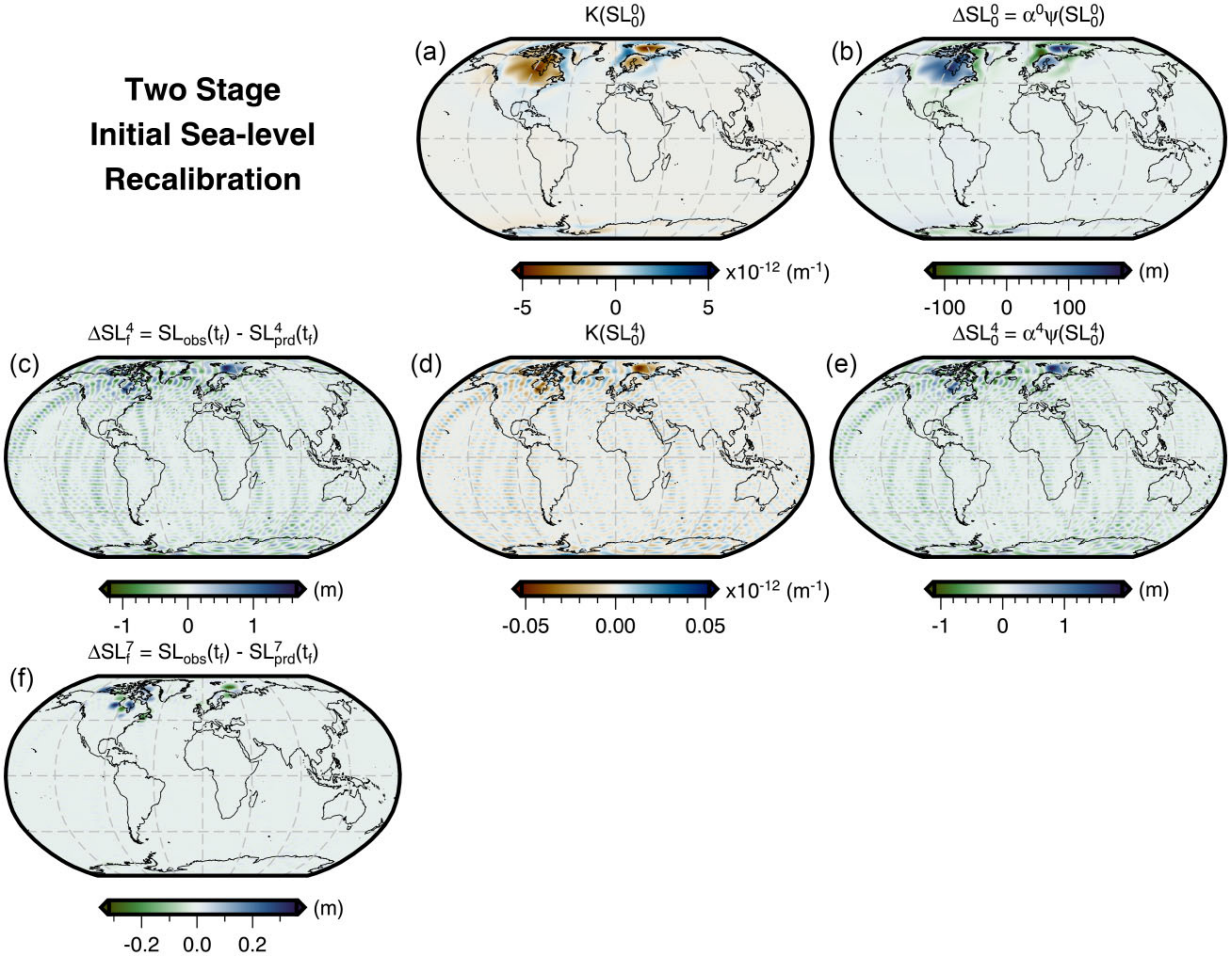
Two-stage initial sea-level recalibration. The panels are similar to Fig. 5, but show results from the two-stage recalibration procedure corresponding to the solid black line in Fig. 4. In the first four iterations, this inversion applies a low-pass filter to the initial sea-level kernel to exclude information from the highest spherical harmonic degrees. Thereafter, this filter is removed and the solution satisfies our convergence criterion by iteration seven. Panel (f) shows the difference between the observed (i.e. target) and predicted present-day sea level following convergence.

In order to avoid these numerical instabilities, as well as to improve the fit of the predicted and *observed* present-day sea level, we use the smoothing described in Section 4 within a two stage inversion procedure. In the first stage, we apply a one-sided Hanning taper to the initial sea-level kernel (eq. 5) and set *l_c_* equal to 60 (eq. 18). As in the example without smoothing, the inversion initially converges rapidly and achieves a similar degree of misfit reduction over 4–5 iterations (Fig. 4a), but now the maximum difference between the predicted and *observed* present-day sea level is reduced to ∼1.5 m (Fig. 4b). Fig. 6(c) shows that there remains some ringing artefacts radiating from points of highly localized discrepancy that have peak amplitudes of ∼1 m and are associated with the truncation of the spherical harmonic transformation. To further reduce these discrepancies and artefacts, we perform a second stage of the inversion that includes higher degree information. We now use the full, unfiltered initial sea-level kernel and, over the course of another four iterations, the misfit decreases by a further two orders of magnitude. The maximum difference between the predicted and *observed* present-day sea level is 0.38 m and satisfies our convergence criteria (Fig. 6). Although minor ringing artefacts persist, this second stage of the inversion procedure reduces their maximum amplitude to only ∼0.05 m. Thus, we now have a new initial sea level that, when used with our 1-D viscosity model, predicts present-day sea level that is consistent with that of the original forward GIA simulation for the filtered and bounded 3-D viscosity model.

**Figure 7.**
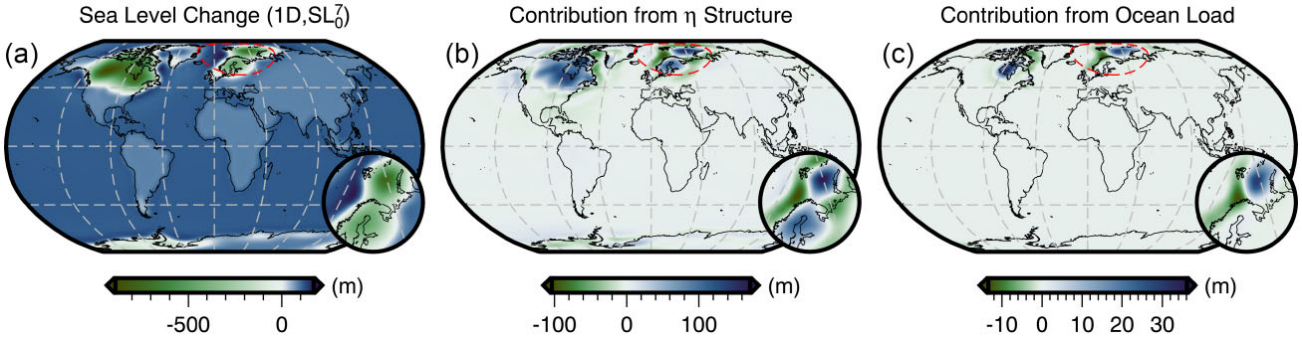
Influence of lateral viscosity variations and initial sea level. (a) The total sea-level change from 26 ka to 1950 CE for a simulation using the 1-D viscosity structure and recalibrated initial sea level, $SL^{7}_{0}$. (b) The contribution to the difference in the final sea level from Fig. 3(c) that is solely due to adopting the filtered and bounded 3-D viscosity model instead of the 1-D model. This field is obtained by differencing the sea-level change in Figs 3(a) and 7(a), and is equivalent to $SL^{0}_{0}-SL^{7}_{0}$. (c) The contribution to the difference in final sea level from Fig. 3(c) that is solely due to use of an incorrect initial sea level. This latter contribution can adversely affects the use of sea-level observations to image mantle structure.

Using results from the forward GIA simulation that adopts the 1-D viscosity model and the recalibrated initial sea level ($SL_{0}^{7}$), we can now decompose the difference in final sea level for our two original forward simulations (Fig. 3c) into a component that is due to the different viscosity models and another arising from our erroneous assumption of the same initial sea level ($SL_{0}^{0}$). The contribution of the former is shown in Fig. 7(b) and is obtained by differencing the total sea-level change predicted by the forward simulation with 3-D viscosity (Fig. 3a) from the 1-D case using the recalibrated initial sea level (Fig. 7a). Within numerical accuracy, this is equivalent to the difference between the two initial sea levels ($SL_{0}^{0}$ and $SL_{0}^{7}$). This difference (Fig. 7b) is more subdued within and near the former marine ice sheets in comparison to that of simulations using the same initial sea level (Fig. 3c). For example, the difference in the total sea-level change within the marine portion of the former Fennoscandian ice sheet has decreased from ∼200 to ∼170 m. This difference, and others shown in Fig. 7(c), reflect changes in the history of loading and unloading of the oceans, including their viscoelastic response, resulting from the use of different initial sea level ($SL_{0}^{0}$ and $SL_{0}^{7}$) and viscosity models (1-D and 3-D) that predict the same present-day sea level. The overall pattern of 3-D-minus-1-D sea-level change, nevertheless, remains similar and our prior discussion in Section 6.1 on the influence of relative changes in viscosity therefore remains valid.

Through this example, we have demonstrated the success of the initial sea-level recalibration based on the adjoint method and gradient based optimization, which can be implemented in more complex inversions that also updates other model parameters (e.g. mantle viscosity; Lloyd *et al.* in preparation). Although we focused here on results obtained using the method of steepest descent, we have also tested the conjugate gradient method and found that it produces consistent results (Fig. 4). In all instances, the degree of success of the inversion relies on a suitable smoothing strategy that assimilates and matches lower spherical harmonic degree structure first and then systematically introduces higher degree structure in latter iterations. This approach is similar to that taken in adjoint seismic tomography (e.g. Pratt 1999; Fichtner *et al.*^2009^; Zhu *et al.*^2015^), where progressively shorter period waveforms are assimilated in later iterations. In our inversion strategy, however, we have chosen to control the length scale of new information by low-pass filtering the gradient as opposed to filtering the predicted and observed data (e.g. Pratt 1999; Fichtner *et al.*^2009^; Zhu *et al.*^2015^). Finally, armed with two suitably calibrated initial sea levels ($SL_{0}^{0}$ and $SL_{0}^{7}$), we can now explore viscosity sensitivity kernels for sea-level observations in order to understand how these data will likely inform inversions for 3-D mantle viscosity.

### 6.3 Viscosity sensitivity kernels for sea-level observations adopting a 1-D viscosity model

We begin by examining 3-D viscosity sensitivity kernels that relate changes in sea-level observations to viscosity perturbation within the solid Earth adopting a 1-D viscosity model. We recall that these kernels are calculated following eq. (4) and that they are a linear approximation of the Fréchet derivative relative to the assumed viscosity structure, in which the range of their validity has been explored by Crawford *et al.* (2018), Tromp & Mitrovica (2000) and in Appendix C2. We consider two types of sea-level observations and hence two types of viscosity sensitivity kernels. First, an absolute sea-level point measurement at a given time, *t*_obs_, which was initially discussed by Crawford *et al.* (2018). Secondly, a relative sea-level point measurement that dates from a given time, *t*_obs_, but is defined as the difference in sea level between *t*_obs_ and present day, *t_p_*, and therefore reflects the change in sea level between these two times. This latter type generally corresponds to observations made in the field, since elevations of palaeo sea-level indicators are measured relative to present-day sea level. We note that both absolute sea level and relative sea level are spatially variable fields. Recall that calculations of relative sea-level viscosity kernels only require a change to the adjoint load (Section 3.1) and thus, eq. (4) remains unchanged. In addition, sensitivity kernels for relative sea-level observations can also be constructed by differencing those for two absolute sea-level observations [i.e. *K*_SL_(**x**_obs_, *t*_obs_) − *K*_SL_(**x**_obs_, *t_p_*); Section 3.1].

In order to explore how relative sea-level measurements might sense Earth’s viscosity structure and how these sensitivities differ from those of absolute sea-level measurements, we examine the viscosity sensitivity kernels in three settings: (1) in the near field of the Fennoscandian ice sheet at Andenes, Norway, (2) on the forebulge of the Laurentide ice sheet at Barbados and (3) in the far field at the Seychelles. To aid with intercomparison of the kernels, we consider ages of 10 and 0 ka for the absolute sea-level observations and 10–0 ka for the relative sea-level observation. For further simplicity, we adopt our 1-D viscosity model (Section 5.1), its newly determined initial sea level ($SL_{0}^{7}$), and perform the forward and adjoint GIA simulations as described in Section 5. Due to rotational symmetry of the 1-D solid Earth structure, differences in the viscosity kernel for each site reflects only its location with respect to the evolving ice sheet and oceans. By not adopting a 3-D viscosity model at this stage, we ensure that any laterally varying features of the kernel are related to the induced deviatoric stresses and not their dependence on *η*^−1^. Furthermore, although the adjoint method provides the contribution to the kernel, δ*K*, at each individual time step, yielding insight into the deformational processes that influence the observation at each point in time, we examine these kernels in their time-integrated form, *K*, to obtain a complete picture of the total sensitivity. From a geophysical imaging perspective, it is this time-integrated kernel that we relate to an observation or misfit. Thus, we will focus on building intuition concerning how the dominant physical processes are encoded within the viscosity sensitivity kernel, as well as how the definition of the sea-level observation influences the kernel structure and its dependency on various physical processes. In turn, this intuition will guide how we invert palaeo sea-level observations for 3-D viscosity structure and how we interpret the resulting images in our companion study.

Critical to decoding these kernels is the ability to interpret their meaning. For absolute sea-level observations, positive (negative) kernel values indicate that an increase in viscosity at that location within the Earth leads to an increase (decrease) in sea level at the observation site. For relative sea-level kernels, changes to viscosity affect both sea level at the time the observation was encoded and sea level at the present. This factor can lead to the cancellation of similarly sensed regions and will highlight processes that lead to differences in the sea-level signal between the time of the sea-level observation and the present. In terms of the behaviour of relative sea level, a positive (negative) kernel value indicates that an increase in viscosity at that location within the Earth will increase (decrease) relative sea level at the observation site. The link between relative sea-level kernels and corresponding relative sea-level change is, however, more obscure since it depends on the size and timing of the surface load changes (i.e. ice sheet and ocean) relative to *t*_obs_ and *t_p_*, and whether sea level has risen or fallen over this time window.

**Figure 8.**
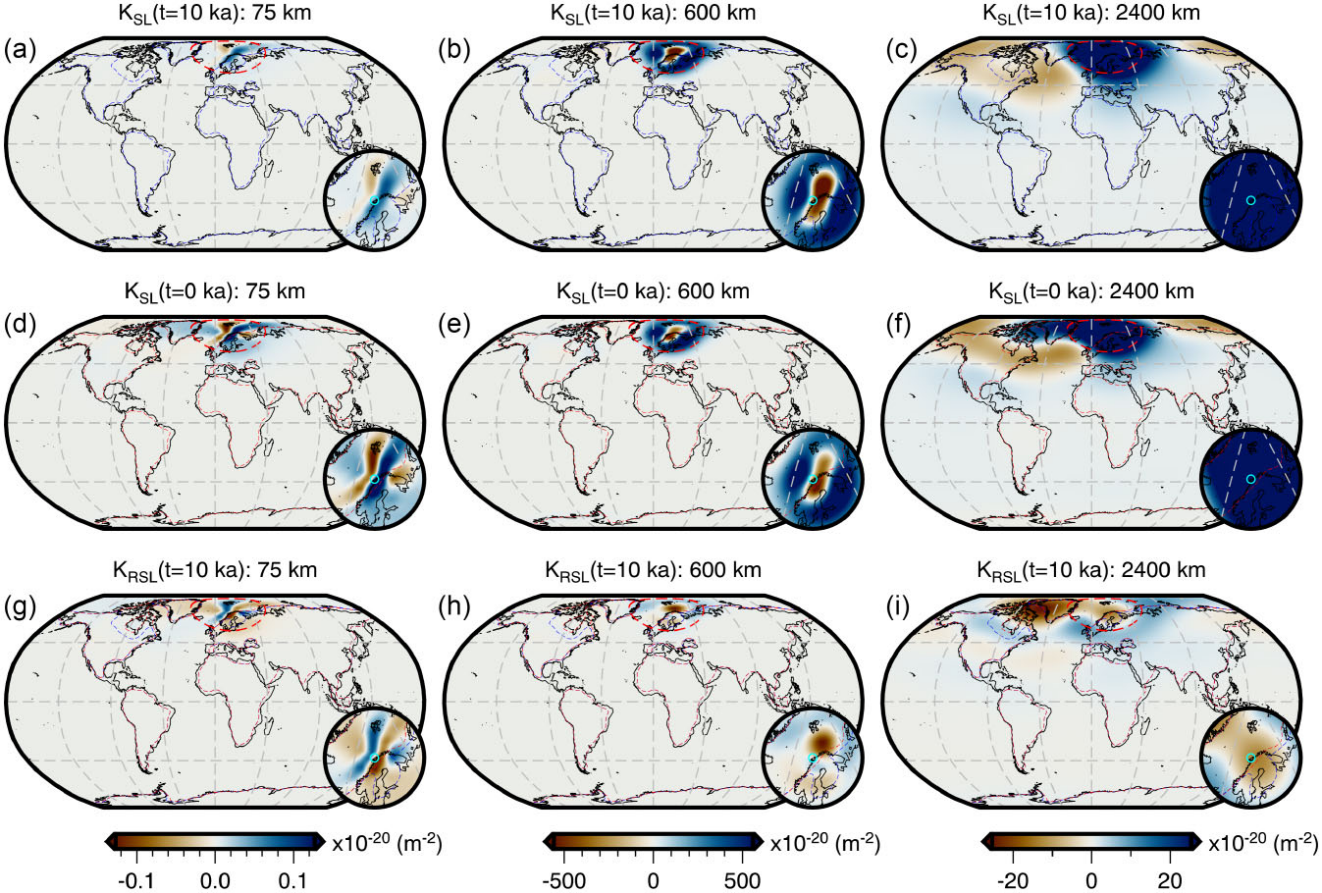
Comparison of viscosity sensitivity kernels for absolute and relative sea-level observations at Andenes, Norway. (a–c) Slices at 75, 600 and 2400 km depth through the viscosity sensitivity kernel for an absolute sea-level observation at 10 ka. The inset map is centred on the observation site (cyan circle) and has a width of 30°. It is extent is shown on the main map as a thick red dashed line. The thin blue dashed line shows the 0 m sea level contour at 10 ka. (d–f) The same, but for an absolute sea-level observation at 0 ka. The thin red dashed line shows the 0 m sea level contour at 0 ka. (g–i) The same, but for a relative sea-level observation from 10 ka. Note that the colour scale for each column is chosen to symmetrically span the range of values for the relative sea-level viscosity sensitivity kernel and thus, regions of high amplitude absolute sea-level sensitivity may be saturated.

**Figure 9.**
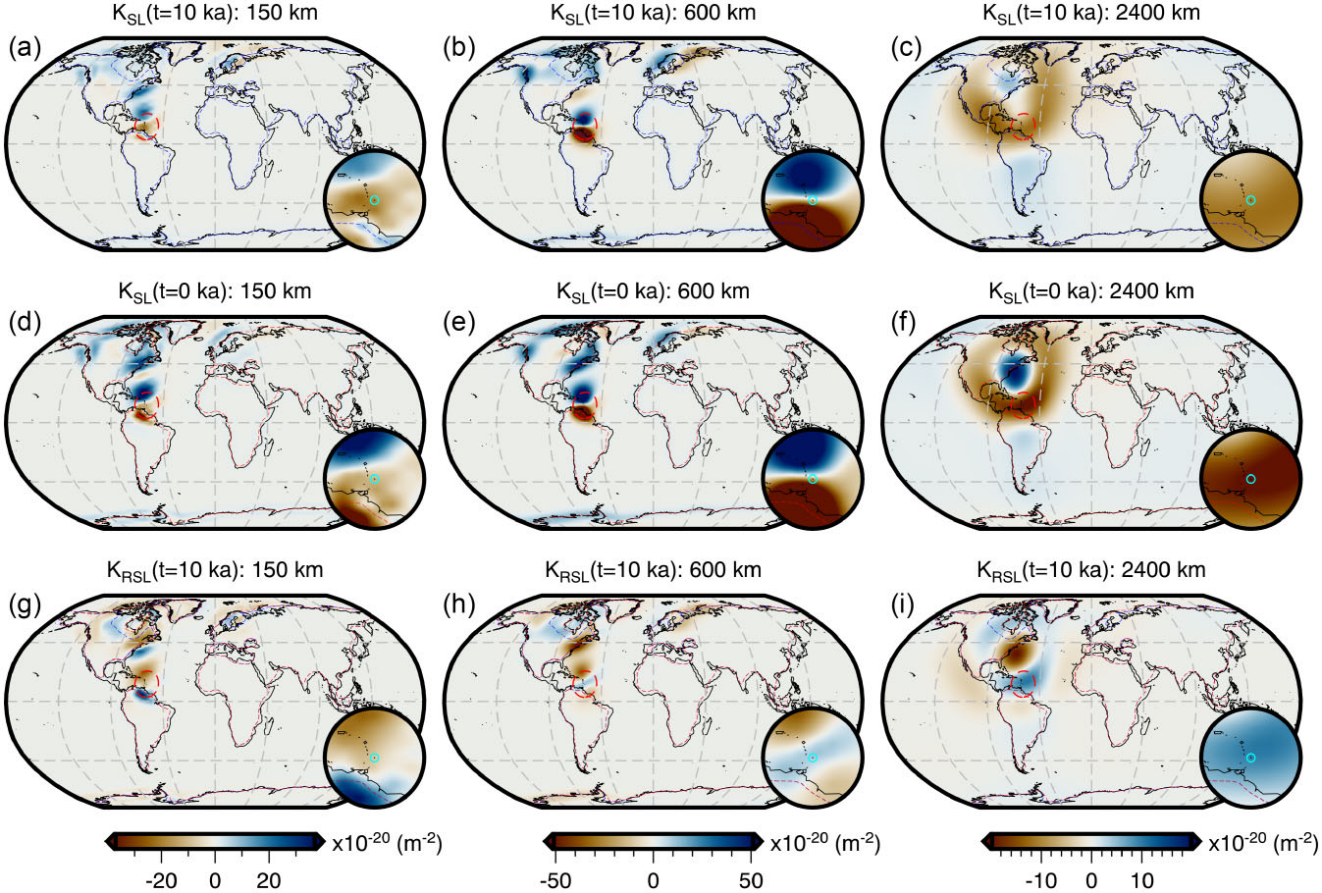
Comparison of viscosity sensitivity kernels for absolute and relative sea-level observations at Barbados. The panels are the same as Fig. 8 except that the shallowest depth slice is now at 150 km and the width of inset map is 20°.

**Figure 10.**
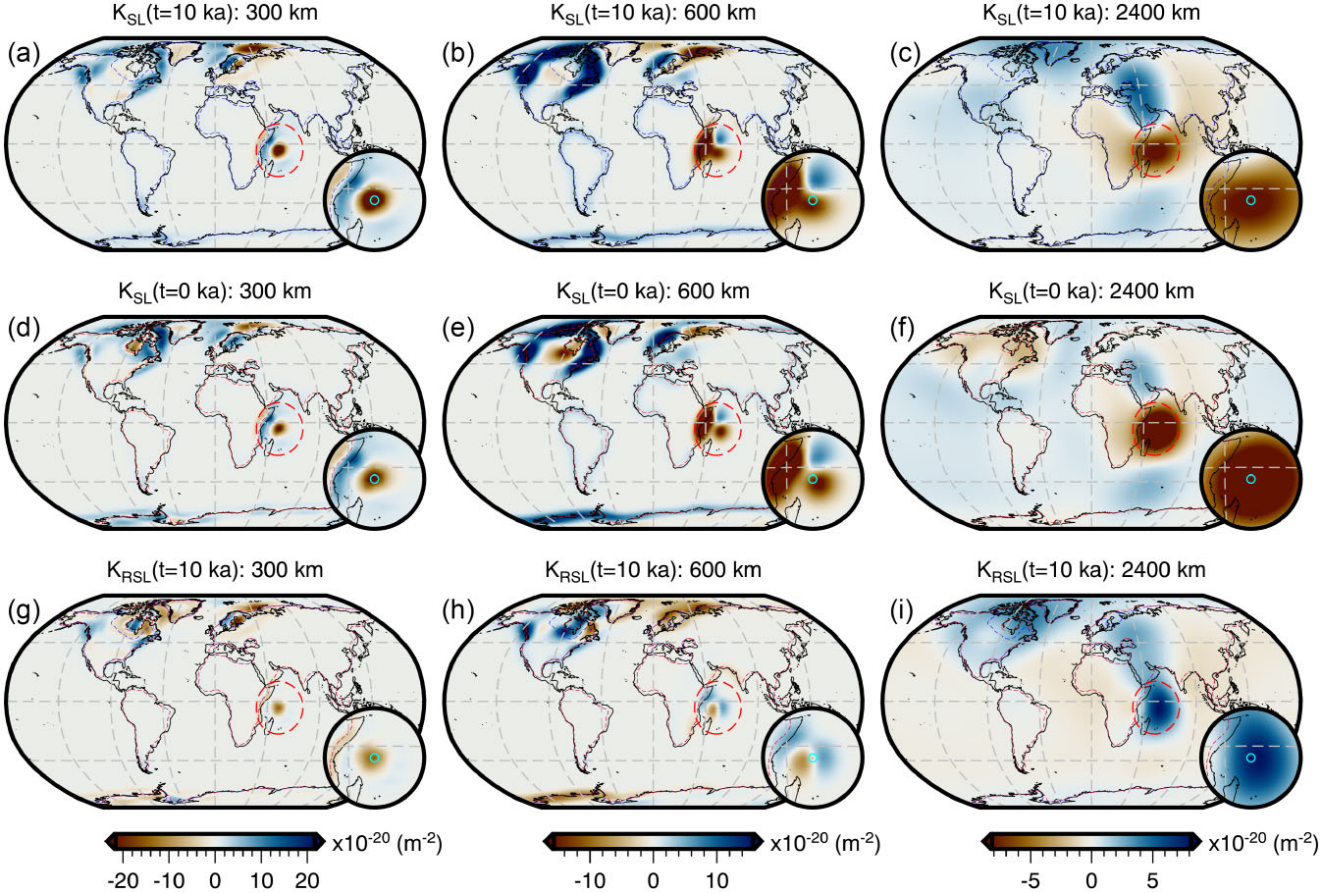
Comparison of viscosity sensitivity kernels for absolute and relative sea-level observations at the Seychelles. The panels are the same as Fig. 8 except that the shallowest depth slice is now at 300 km and the width of inset map is 40°.

A few characteristics appear to be ubiquitous to the viscosity sensitivity kernels for absolute sea-level and relative sea-level observations (Figs 8–10). With regards to absolute sea-level observations, some of these characteristics were originally reported by Crawford *et al.* (2018), but are listed here for completeness. First, the amplitude of the viscosity sensitivity kernels for near-field observation sites are 10–100 times greater than those for far-field observation sites. Secondly, there is sensitivity throughout all depths of the mantle. At shallow depths, peak sensitivities are concentrated beneath the observation site as well as beneath those regions experiencing significant surface-load changes due to the evolving ice sheets and redistribution of the oceans. These regions of sensitivity broaden with depth, consistent with the results of Paulson *et al.* (2005) and Wu (2006). As we approach the core–mantle boundary, far-field observation sites often have *visible* global coverage, while near-field observation sites have higher amplitude sensitivities that are spatially restrictive. Nevertheless, the surface integral of the 3-D sensitivity kernel at a given depth in the deep mantle is typically small compared to shallower depths, which is consistent with past studies that determined 1-D radial sensitivity kernels for mantle viscosity (e.g. Mitrovica & Peltier 1991b; Crawford *et al.*^2018^). It is only when the corresponding 3-D viscosity sensitivity kernels are calculated that one realizes the intuition gained from their 1-D counterparts can be misleading. Instead, 3-D sensitivity kernels for both absolute sea-level and relative sea-level observations have non-negligible sensitivities within the deep mantle and possess unique patterns that reflect the location of the observation site with respect to the surface load changes. Therefore, there exists great promise for imaging not just the upper portion of the 3-D viscosity structure, but also its deepest depths. Third, the existence of positive and negative regions within the viscosity kernels for both types of sea-level observations indicates that there is potential to mask the influence of Earth structure on an observation, which has previously been noted in forward modelling studies (e.g. Wu & van der Wal 2003). Although these generalizations are broadly correct, there are some deviations and finer-scale structures within the kernels whose origin is not easily discerned. Nevertheless, the structure of the kernels reflects physical processes that influence the behaviour of sea level at the observation site, which we will now discuss for three different settings.

#### 6.3.1 Viscosity sensitivity kernels for Andenes, Norway

In our first example, we consider a sea-level observation site at Andenes, Norway, where local sea-level has fallen over the last 10 kyr of the simulation. Fig. 8 shows depth slices at 75, 600 and 2400 km through the viscosity sensitivity kernels for absolute sea-level observations at 10 and 0 ka, as well as for a relative sea-level observation covering the period 10–0 ka. These kernels are dominated by high-amplitude features that reflect the nearest regions of ice-mass change. More distant load changes, such as the shrinking Laurentide ice sheet, generate sensitivities within the underlying mantle that have a similar magnitude to those observed in kernels for far-field observations at the same location (e.g. Seychelles; Fig. 10). Although these low-amplitude sensitivities are present within the kernel and may have relevance for imaging, we will focus on higher amplitude features at each depth and begin our discussion with the absolute sea-level observations at 10 and 0 ka.

Within the lithosphere, the maximum amplitude of the kernel is small relative to underlying regions of the sublithospheric mantle (Figs 8a and d). This reflects the high viscosity of the lithosphere (∼1.5 × 10^26^ Pa·s), which essentially behaves elastically at the time-scale of the simulation, and thus has a negligible effect on absolute sea-level observations if its viscosity is perturbed. For both absolute sea-level observations (10 and 0 ka) along profile A (Fig. S15), there is a general pattern of positive kernel values beneath Andenes extending to the southeast and negative kernel values to the northwest that reach a peak amplitude greater than 1000 × 10^−20^ m^−2^ at 300 km depth and persist down to ∼550 km. Further to the northwest, the kernel again becomes positive, though its amplitude is much smaller.

Across this region, the structure of the viscosity sensitivity kernels for absolute sea-level observations reflect a number of linked processes. First, the positive kernel region beneath and to the southeast, underlying the former Fennoscandian ice sheet, indicates that an increase to viscosity there will lead to an increase in absolute sea level at Andenes. This relationship follows from the fact that a stiffer mantle in this region will lead to slower uplift during deglaciation and hence higher absolute sea level. Secondly, the negative kernel region to the northwest indicates an increase in viscosity there will decrease absolute sea level at Andenes. We suggest that this is because stiffer viscosities will modify the behaviour of the forebulge, reducing its amplitude and increasing its width either side of the hinge point. As a result, the solid Earth at Andenes will become higher and absolute sea level will decrease. Finally, the transition back to positive kernel values further to the northwest again indicates that an increase in viscosity here will result in an increase in absolute sea level at Andenes. We speculate that this is because a stiffer mantle beneath this region would lead to less subsidence of the ocean basin, with the formerly accommodated water mass now redistributed over the global ocean leading to an increase in absolute sea level at Andenes.

From ∼550 to 670 km depth (i.e. the base of the transition zone) the dominant features within the viscosity sensitivity kernels for the absolute sea-level observations flip polarity. Here, the kernels are negative beneath Andenes, while the surrounding area is now positive (Figs 8b, e and Fig. S15). This negative region of the kernel indicates that an increase in viscosity there will decrease absolute sea level at Andenes. At the same time because this region underlies the former Fennoscandian ice sheet where greater solid Earth uplift occurs, we more intuitively expect an increase in viscosity to decrease solid Earth uplift and hence increase absolute sea level, similar to what is indicated by the kernels at shallower depth. Thus, a negative kernel value beneath the ice sheet initially seems puzzling. We suggest that this behaviour occurs due to coupling of the lower viscosity (5 × 10^20^ Pa·s) upper mantle and transition zone with the higher viscosity (5 × 10^21^ Pa·s) lower mantle, which is a consequence of the boundary condition that the change in displacement, **u**, across a solid-to-solid boundary is **0** (eq. 2.14 of Al-Attar & Tromp 2013; i.e. a void cannot form;). In part to satisfy this boundary condition and in order to match deformation at the top of the higher viscosity lower mantle, vertical uplift above the 670 km viscosity discontinuity must decrease relative to that predicted for an earth model with a uniform viscosity of 5 × 10^20^ Pa·s. Increasing mantle viscosity just above 670 km depth lowers the viscosity contrast and shrinks the required reduction in vertical uplift necessary to satisfy the boundary condition. Negative sensitivities between ∼550 and 670 km depth demarcate the region where an increase in viscosity will allow for greater overall uplift of the solid Earth and hence lower absolute sea level. This interpretation is consistent with the kernels switching back to positive at and below 670 km depth (Figs 8c, f and S15), where a decrease in viscosity (i.e. a reduction of the viscosity contrast) leads to a decrease in absolute sea level at Andenes. This simple depth-varying structure (i.e. +, −, +) of the kernel illustrates how the change in absolute sea level at the observation site due to viscosity perturbation in one region can be masked by an appropriately sized perturbation in another region. It is clear that this masking behaviour occurs with other observations (e.g. relative sea level) and thus, may explain why Wu & van der Wal (2003) found that relative sea-level observations near the centre of large load changes may be unable to detect lower mantle viscosity perturbations if the upper mantle and transition zone is perturbed in the opposite sense. It is important to note, however, that our sensitivity kernels are calculated for a different 1-D viscosity structure than Wu & van der Wal (2003) and that a proper comparison would require consideration of the full 3-D structure of the kernel.

Surrounding the negative region between ∼550 and 670 km depth, the kernel is positive. Although subsidence due to forebulge collapse does occur to the northwest of Andenes, we find that any vertical deformation associated with this process dissipates by ∼325 km depth and, at deeper depths, is characterized by low-amplitude uplift. Thus, we suggest that positive kernel values within the broader transition zone reflect the longer wavelength load change associated with deglaciation of the Fennoscandian ice sheet rather than forebulge collapse. From this standpoint, an increase in viscosity in this positive kernel region will reduce solid Earth uplift and increase absolute sea level.

Finally, at depths of 670 km and greater (Figs 8c, f and S15), the viscosity sensitivity kernel beneath northern Europe is again positive, indicating that an increase in viscosity there will increase absolute sea level at Andenes. The amplitude of the kernel is smaller due to the higher viscosity of the lower mantle (i.e. the *η*^−1^ dependence of eq. 4) and greater distance from the surface load change. The latter is a result of attenuation, which also more strongly dissipates the higher spherical harmonic degrees of deformation. Thus, deformation in the lower mantle beneath northern Europe is controlled by the lower spherical harmonic degree components of the shrinking Fennoscandian ice sheet. By increasing the viscosity beneath northern Europe, the extent of solid Earth uplift due to unloading of the ice sheet is reduced and hence absolute sea level at Andenes increases. In contrast, the kernels are negative beneath northern North America. Through similar logic, an increase in viscosity there will increase absolute sea level above that region, thereby siphoning water mass from other parts of the global ocean and, in turn, decreasing absolute sea level at Andenes.

With these considerations in mind, we next turn our attention to the viscosity sensitivity kernel for a relative sea-level observation spanning 10–0 ka (Figs 8g–i) and begin by addressing the relationship between absolute and relative sea-level change and their associated sensitivity kernels. We recall that sea level has fallen at Andenes over the final 10 kyr of the simulation, such that relative sea level is positive. Directly beneath Andenes at 75 km depth (Fig. 8g), the kernel is negative, indicating that an increase in viscosity there will decrease relative sea level at the observation site. To make sense of this result, we recall that the kernel for a relative sea-level observation is equivalent to the difference between the kernels for absolute sea-level observations at 10 and 0 ka. Within this region of the mantle, both absolute sensitivity kernels are positive, indicating that an increase in viscosity there will increase absolute sea level at the observation site. Furthermore, since *K_S_L*(*t* = 10 ka) < *K_S_L*(*t* = 0 ka), the same increase in viscosity will result in a greater increase in absolute sea level at 0 ka compared to 10 ka. When sea level has fallen, this behaviour reduces the difference between absolute sea level at 10 and 0 ka and thereby decreases the 10–0 ka relative sea-level change, consistent with negative kernel values.

Focusing now on its general structure, we see that the relative sea-level kernel is similar to those of absolute sea-level observations, but with flipped polarities (Figs 8g–i). This pattern indicates that, in most regions, the absolute sea-level observation at 0 ka has greater sensitivity to mantle viscosity than its equivalent at 10 ka. One notable exception is observed beneath the northern marine-based portion of the Fennoscandian ice sheet at 600 km depth (Fig. 8h). Here, kernels for both types of sea-level observations are negative and hence the kernel for a sea-level observation at 10 ka has a greater amplitude. This difference occurs because the ice sheet disappeared from this region prior to 10 ka (Fig. 1b) and illustrates that the amplitude of the kernel for absolute sea-level observations is greater when the time between the same surface-load change and observation time is smaller. In contrast, immediately southeast of Andenes, a localized region at 600 km depth does change polarity in the kernel for relative sea level due to further ice mass loss occurring after 10 ka (Fig. 1c). These effects demonstrate that the spatiotemporal history of loading has an important influence on the structure of both types of kernels.

#### 6.3.2 Viscosity sensitivity kernels for Barbados

In our second example, we consider an observation site at Barbados, which lies at the edge of the forebulge of the Laurentide ice sheet. Given its proximity to the ice sheet, it seems natural to assume that sea-level observations here are sensitive to many of the same deformational processes as the site at Andenes. However, the different location relative to the load changes causes these deformational process and potential perturbations to the 1-D viscosity structure to influence sea level at Barbados in a different manner. Fig. 9 shows that sensitivity to mantle viscosity is focused beneath the observation site and the closest regions of surface load change (i.e. the Laurentide ice sheet), with minor sensitivity beneath the Fennoscandian and West Antarctic ice sheets. Although we will focus on the higher amplitude features, it is worth noting that the Barbados absolute sea-level observation at 10 ka has little sensitivity to mantle viscosity beneath the West Antarctic ice sheet relative to that beneath the Fennoscandian ice sheet at 150 and 600 km depth (Fig. 9a,b). This is because much of the ice-mass change in West Antarctica occurs after 10 ka, where as much of the Fennoscandian ice-mass change occurs prior to 10 ka (Fig. 1). In contrast, for the Barbados absolute sea-level observation at 0 ka, the kernel amplitude beneath West Antarctica and Fennoscandia is similar (Figs 9d and e). Thus, this example highlights the complex inter-play of distance (e.g. observation site to load change) with the load change magnitude, timing, and spatial extent in controlling the amplitude of the sensitivity kernel. As a result of these same factors, we observe that the peak amplitudes of the sensitivity kernels for Barbados are an order of magnitude smaller than those of Andenes. This amplitude difference will require careful attention in future work that images mantle viscosity using sea-level data.

Viscosity sensitivity kernels for the two absolute sea-level observations at 10 and 0 ka are more complex for Barbados than Andenes and hence more difficult to interpret. Positive kernel values at 150 and 600 km depth are predominantly observed beneath the former Laurentide ice sheet and north of Barbados (Figs 9a and d), indicating that an increase in viscosity there will raise absolute sea level at Barbados. We speculate that, for areas around the periphery of the former Laurentide ice sheet, a stiffer mantle will lead to slower forebulge subsidence, which causes absolute sea level to be higher elsewhere, including Barbados. High positive kernel values along the transect from Barbados to the Laurentide ice sheet might indicate that a differently shaped forebulge, due to a stiffer mantle, can result in deeper water depths at Barbados. At upper mantle depths, the kernel is near zero or negative beneath Barbados itself (Figs 9a and d), indicating that an increase in viscosity will reduce subsidence of the solid Earth in response to the increased ocean load from deglaciation. Hence, absolute sea level will be lower if the upper mantle is stiffer directly beneath Barbados. This interpretation is consistent with Austermann *et al.* (2013), who showed that a high-viscosity slab in the Caribbean subduction zone acts to reduce local sea level.

Finally, at 2400 km depth, deformation is again dominated by long-wavelength load changes that will be primarily related to the shrinking Laurentide ice sheet. Here, the sensitivity kernel has a positive kernel value in the centre of the region between the Laurentide ice sheet and Barbados, which is ringed by negative kernel values (Figs 9c and f). This feature reflects the relative geographic location of the load change and the observation site. Although its full nature is unclear, we note that the boundary from positive to negative kernel values nearest Barbados corresponds to a change from uplift to subsidence of the solid Earth at this depth in the forward simulation.

We next turn our attention to the viscosity sensitivity kernel for a relative sea-level observation spanning 10–0 ka in Barbados (Figs 9g–i). In this example, sea level has risen at the observation site (i.e. relative sea level is negative) between 10 and 0 ka during the simulation. Directly beneath Barbados at 150 km depth, the sensitivity kernel for relative sea level is negative, indicating that an increase in viscosity there will decrease relative sea level (Fig. 9g). At this same location, the kernels for absolute sea-level observations are also negative with *K*_SL_(*t* = 10 ka) < *K*_SL_(*t* = 0 ka). Thus, for the same viscosity increase, the absolute sea level observation at 10 ka will decrease more than that at 0 ka, increasing the sea-level offset spanning 10–0 ka. Given this behaviour, along with the fact that sea level is rising, the relative sea level at the observation site will become more negative (i.e. decrease) as viscosity increases, consistent with negative kernel values for the relative sea-level observation.

At depths of 150, 600 and 2400 km, we find that amplitudes across the footprint of the former Laurentide ice sheet are more uniform at a given depth in comparison to the two sensitivity kernels for absolute sea-level observations. At 150 km depth, there are stronger changes in polarity at continent–ocean boundaries along the northeastern United States and northern South America, which we suggest relate to forebulge collapse and continental levering, respectively. Meanwhile at 600 km depth, we observe an intriguing pattern of negative, positive and then negative kernel values in the vicinity of Barbados, which is roughly orthogonal to the great circle path connecting Barbados to Hudson Bay. Because Barbados lies at the edge of the Laurentide forebulge this pattern likely relates to the dynamics of forebulge collapse (Fig. 9h). At 2400 km depth, we note that the amplitude of the viscosity sensitivity kernel is only a factor of two smaller than that observed at 150 km depth (Fig. 9i). As we will see in the next example, this pattern of non-negligible sensitivity to deep mantle viscosity structure is a ubiquitous feature of these sensitivity kernels.

#### 6.3.3 Viscosity sensitivity kernels for Seychelles

In our final example, we consider a far-field observation site in the Seychelles where sea level has risen during the final 10 kyr of the forward GIA simulation. Fig. 10 shows images of the viscosity sensitivity kernels at depths of 300, 600 and 2400 km for the two absolute sea-level observations at 10 and 0 ka, as well as for a relative sea-level observation spanning the period 10–0 ka. We observe two distinct groups of kernels for observations that are located at far-field sites. The first is characterized by a significant continental region lying between the observation site and the dominant region of ice mass change, such that there is no appreciable ocean load change within this intermediate region. As a result, a more diffuse sensitivity pattern develops similar to that observed in the Seychelles example (Fig. 10). The second group occurs for observation sites, such as Tahiti, where the intervening region is predominantly ocean basin. These kernels exhibit an approximately linear, high-amplitude zone of sensitivity between the site and locations of ice-mass change (Crawford *et al.*^2018^), which is reminiscent of *banana–doughnut* kernels in seismology (Dahlen *et al.*^2000^). While we have not illustrated an example of this second group here, our kernel for Barbados has some similar features (Fig. 9).

Within the viscosity sensitivity kernel for absolute sea-level measurements at 10 and 0 ka, there are again a number of local features that reflect a range of deformational processes. First, the negative kernel value at all depths beneath the Seychelles reflects the fact that, during deglaciation, the ocean load increases and a stiffer mantle therefore results in less subsidence and lower absolute sea level, similar to Barbados. Furthermore, the negative kernel values beneath the observation site are observed throughout the mantle, suggesting that, in contrast to the Andenes example, coupling between the upper and lower mantle has limited influence on the behaviour of sea level at this site. We speculate that this aspect occurs because the load change due to the ocean, though long wavelength, is small in amplitude relative to that of the Fennoscandian ice sheet. Meanwhile, surrounding the Seychelles and beneath the ocean, the sensitivity kernel is positive at 300 km depth (Figs 10a and d), indicating that an increase in viscosity there will increase absolute sea level. This relationship suggests that a stiffer mantle in this region will result in less subsidence of the solid Earth due to the growing ocean load, with the corresponding reduction in local ocean capacity raising absolute sea level at the observation site. Further to the west, we see a positive-to-negative polarity change at 300 km depth crossing from offshore to onshore east Africa (Figs 10a and d). This pattern reflects the influence of continental levering on the behaviour of sea level at the Seychelles, with an increase in viscosity causing deformation across the coastline to become lower amplitude and longer wavelength. We suggest that the Seychelles are sufficiently close to the east African shoreline to sense this reduction in offshore subsidence during deglaciation, raising the solid Earth and reducing absolute sea level at the observation site.

The upper mantle and transition zone kernels for absolute sea-level observations at both 10 and 0 ka have similar amplitudes within the vicinity of the ice sheets, with the highest values occurring beneath their peripheries. We argue that this sensitivity pattern is related to ocean siphoning (Mitrovica & Milne 2003), in which a higher viscosity leads to slower subsidence of the peripheral bulges and hence higher absolute sea level in the far field. Additionally, the kernel at 0 ka exhibits a negative anomaly beneath Hudson Bay. This area is rebounding in response to glacial unloading and, following the demise of the Laurentide ice sheet, continuing uplift will expel water from Hudson Bay and cause absolute sea level to rise in the far field.

Figs 10(g)–(i) also shows the viscosity sensitivity kernel for a relative sea-level measurement in the Seychelles dating from 10 ka. Although this kernel does exhibit differences in polarity in some locations, the more intriguing feature is its loss of sensitivity throughout some regions of the mantle. For example, at 300 km depth, there is a reduction in regional sensitivity to viscosity and the observation is restricted to sensing local viscosity structure predominantly beneath the observation site and in the vicinity of the east African coastline. This behaviour occurs because the evolution of the local ocean load leads to similar sensitivities for absolute sea-level observations at 10 and 0 ka, except for a slight westward (i.e. inland) shift of the coastline due to shoreline migration. Thus, it is near this region that *visible* sensitivities are focused, indicating that relative sea level in the Seychelles from 10 ka is more sensitive to shoreline migration than continental levering. Similarly at 300 and 600 km depth, there is a reduction in the spatial extent of sensitivities at the peripheries of the ice sheets. We conclude from this pattern that the relative sea-level measurement is less sensitive to forebulge deformation and associated ocean siphoning than its constituent absolute sea-level observations. Through these two examples, we have demonstrated that absolute and relative sea-level observations from the same location and time period can have quite distinct sensitivities to the viscosity structure of the mantle and thus, record distinct deformational processes.

To finish, we return to a striking characteristic of the viscosity sensitivity kernels for both types of far-field sea-level observations, which is that similar amplitude sensitivities are found beneath both the region of the observation site and the regions of ice mass change, even when the two are antipodal. This simple observation has two profound consequences for the use of far-field relative sea-level data to constrain mantle viscosity and, by extension, ice history. First, for a laterally heterogeneous Earth, their use will lead to an estimate that blends local and distal viscosity structure. Such biases in 1-D estimates of mantle viscosity have been demonstrated in forward analyses (e.g. Lau *et al.*^2018^), but the sensitivity kernels in Fig. 10 quantitatively illustrate the reasons for this behaviour. From the perspective of a local relative sea-level dataset, one cannot simply disentangle the influence of the local viscosity structure, which controls the relative local distribution of the ocean load, from the viscosity structure beneath the changing ice sheet and forebulge regions, which dominates the change in total water mass accommodated in the observation region. Furthermore, from the perspective of a global far-field relative sea-level dataset, this bias is exacerbated by the fact that the mantle underlying regions of ice-mass change is sampled by every observation, while the local mantle structure in the far field may only be sampled by a handful of observations. An important consequence is that 1-D estimates of mantle viscosity are likely biased towards the viscosity structure underlying regions of significant load change, such as beneath the Laurentide and Fennoscandian ice sheets.

Secondly, the sensitivity kernels in Fig. 10 hint at a means to minimize sensitivity to distal mantle structure, while preserving sensitivity to local structure. We can envision this idea by imagining that a second observation site exists on the northern coast of Madagascar. While its kernel will locally appear quite different, distal regions will be similar and thus, by differencing kernels (i.e. differencing the relative sea-level measurements), sensitivity is minimized to distal mantle structure whilst being locally enhanced. This thought experiment demonstrates the potential power of differential relative sea-level measurements for constraining local mantle rheology (e.g. Nakada & Lambeck 1989).

### 6.4 Viscosity sensitivity kernels for relative sea-level observations adopting a 3-D viscosity model

Now that we have gained some insight into the nature of viscosity sensitivity kernels for absolute and relative sea-level observations on a 1-D radial Earth, we turn our attention to exploring the effects of lateral variability in viscosity, which will begin to reveal the non-linear nature of the viscosity Fréchet derivatives. Importantly, these results represent the first time that global 3-D viscosity sensitivity kernels for absolute and relative sea-level observations have been robustly calculated for a 3-D viscosity model, following in the footsteps of recent kernels for the rate of change of the degree 2 zonal harmonic of Earth’s geopotential ($\dot{J}_2$; Kim *et al.*^2022^).

**Figure 11.**
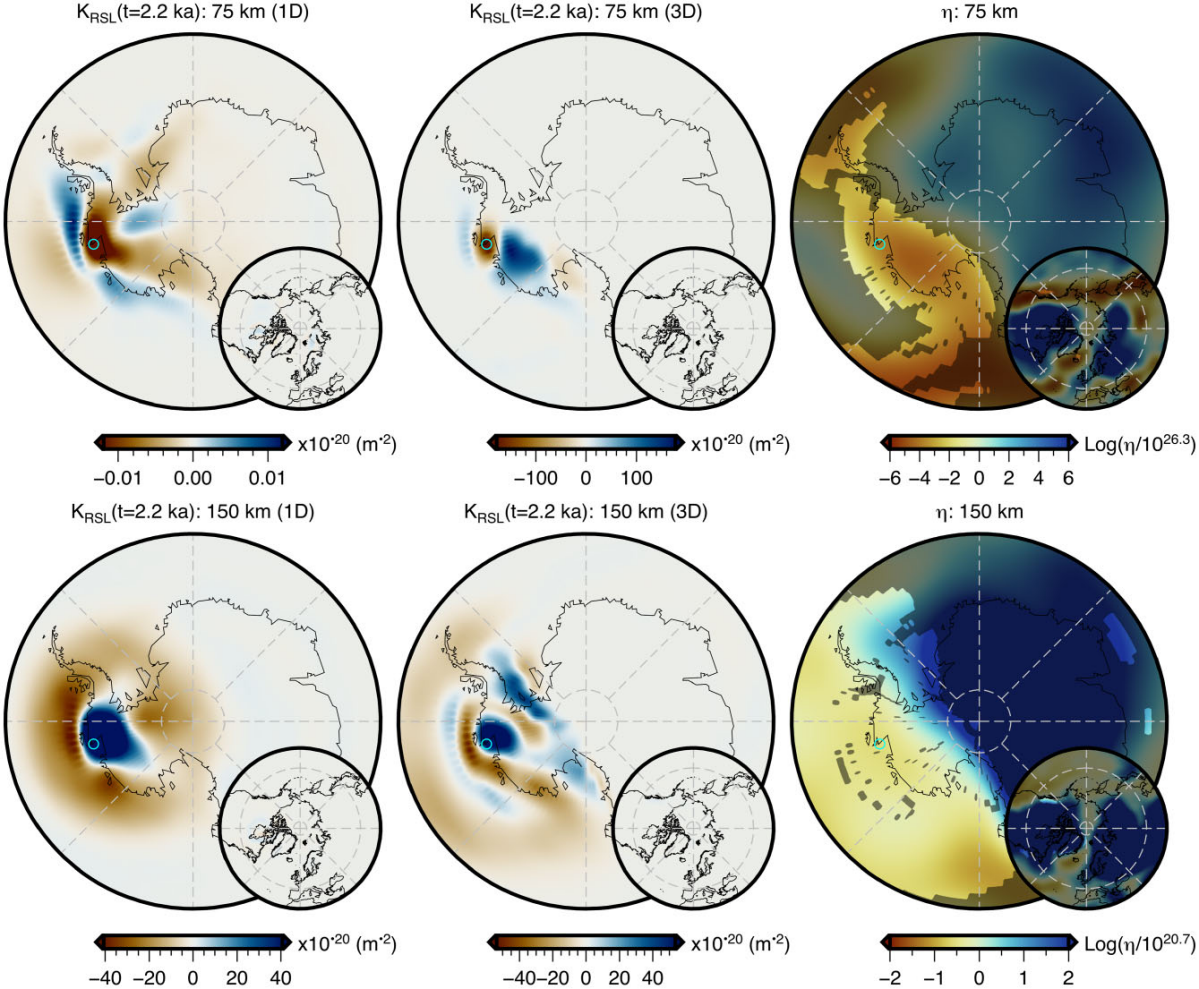
Relative sea-level viscosity sensitivity kernels for 1-D and 3-D viscosity structure. Slices at 75 and 150 km depth through the viscosity sensitivity kernels for a relative sea-level observation on an unnamed island in the Amundsen Sea Embayment (cyan circle) dating to 2.2 ka (Johnson *et al.*^2008^). The first column shows the sensitivity kernel obtained when assuming our 1-D viscosity model (Section 5.1 and Fig. S4) and the second column shows the sensitivity kernel obtained when assuming our filtered and bounded 3-D viscosity inference (Fig. 2). It is this 3-D viscosity structure that is shown in the third column and regions where the amplitude of the sensitivity kernel is less than 10^−20^ m^−2^ are shaded in grey. The inset map is centred on the North Pole and has a width of 90°.

**Figure 12.**
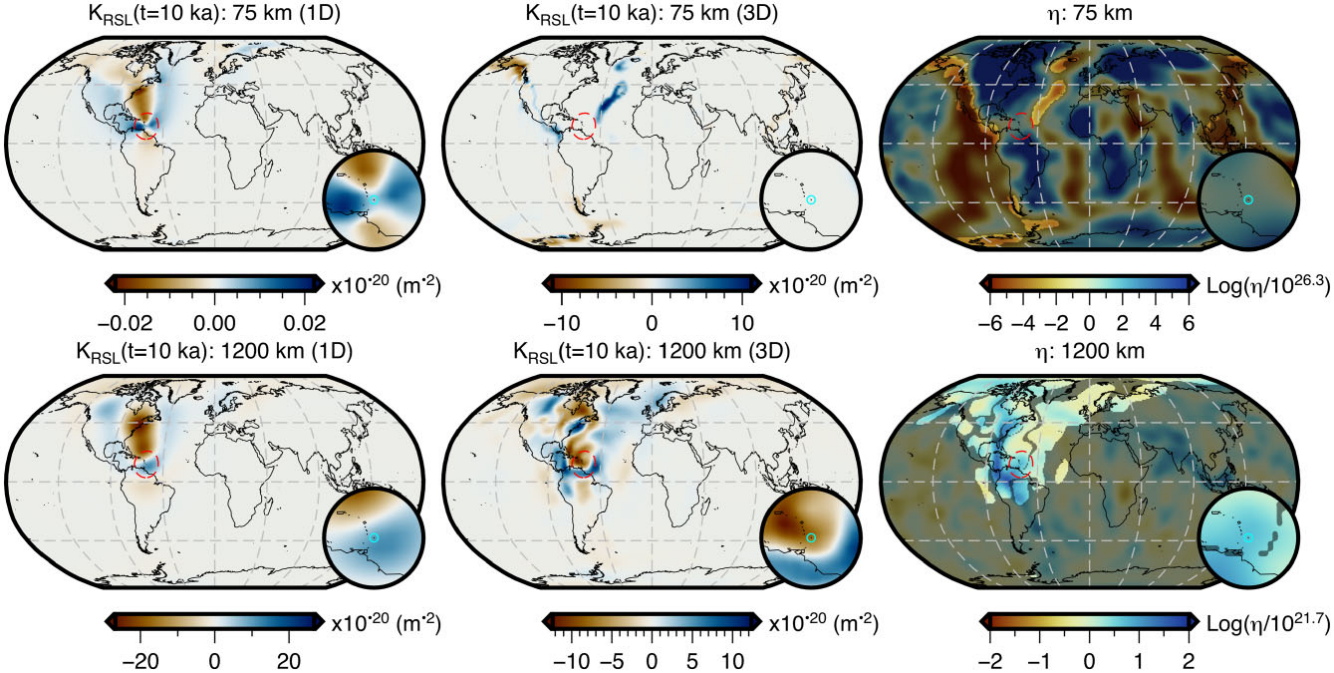
Relative sea-level viscosity sensitivity kernels for 1-D and 3-D viscosity structure. The panels are similar to Fig. 11, but for a relative sea-level observation located at Barbados dating to 10 ka. Here, we show slices at 75 and 1200 km depth with an inset map centred on Barbados that has a width of 20°.

Through two examples, we investigate the influence of geodynamic features including hotspots, slabs and variable lithospheric thickness, which are likely to be characterized by variations in viscosity structure. Although these sources of viscosity heterogeneity influence the sensitivity kernels for both types of sea-level observations (e.g. Figs 11–12 and S7–S18), we focus on those for relative sea-level observations since they form the foundation of the palaeo sea-level record and will be used to invert for 3-D mantle structure in our companion study (Lloyd *et al.* in preparation). Despite using a long-wavelength inference of Earth’s 3-D viscous structure, the kernels for both types of sea-level observations represent a more realistic quantification of observational sensitivity to viscosity compared to those based on a 1-D radial viscosity model (Section 6.3 and Crawford *et al.*^2018^).

In our first example, we consider a relative sea-level observation from the Amundsen Sea Embayment of Antarctica that dates to 2.2 ka (Johnson *et al.*^2008^). In this region, seismically slow mantle wave speeds (Lloyd *et al.*^2020^) and rapid uplift rates recorded by continuous GNSS stations installed on bedrock suggest the presence of a low viscosity (∼10^18^ Pa·s) upper mantle and transition zone (Barletta *et al.*^2018^). To first order, this feature is present in our filtered and bounded 3-D viscosity inference (Fig. 2), although it lacks the lowest of viscosities and finer scale structure that has been imaged by regional seismic tomography models (e.g. Lloyd *et al.*^2015^, ^2020^; Lucas *et al.*^2020^). Nevertheless, it still demonstrates the dramatic influence that even this modest degree of lateral viscosity heterogeneity can have on the structure of the viscosity sensitivity kernel (Fig. 11).

Inclusion of lateral viscosity variability causes a dramatic increase in the amplitude of the sensitivity kernel at shallow depths (e.g. 75 km) from ±0.01 × 10^−20^ to ±180 × 10^−20^ m^−2^, which reflects an increase in viscous deformation at this depth due to a weaker regional viscosity structure. In addition, we find that kernels based on the 3-D viscosity model are characterized by spatially restricted, more focused features that exhibit greater complexity with depth. This pattern reflects the length scale of deformation present within the simulation and is controlled by the interaction between the viscosity structure and the distribution and magnitude of the surface-load changes. Furthermore, the nearly pure elastic response of the thick East Antarctic lithosphere strongly zeros out the sensitivity to viscosity within this region (see Fig. 11 at 150 km depth).

In our second example, we consider a hypothetical relative sea-level observation from Barbados that dates to 10 ka. Barbados represents another end member of the plate tectonic regime, as it lies along the Caribbean subduction zone where cold, high-viscosity oceanic lithosphere is subducted into the mantle. The presence of this slab has previously been argued to suppress local viscous deformation and to reduce sea-level change due to local ocean loading (Austermann *et al.*^2013^). Although our filtered and truncated 3-D viscosity model does not have the resolution to fully capture the downgoing South American plate (Fig. 2), sufficient structure is present to capture its likely effects on the viscosity sensitivity kernel.

In Fig. 12, we see that introduction of 3-D structure beneath Barbados results in negligible sensitivity at 75 km depth within high-viscosity regions and indicates that, for these load changes and at these timescales, elastic deformation dominates within this region of the mantle. In contrast, weaker viscosity regions that are located further from the observation site exhibit notably higher sensitivity because they undergo greater viscous deformation. For example, portions of the mid-Atlantic ridge have positive kernel values, indicating that an increase in viscosity there would lead to an increase in relative sea-level at Barbados as mantle material cannot escape as efficiently along the mid-ocean ridge axis. This behaviour of distal viscous deformation more strongly influencing relative sea level at Barbados than local viscous deformation is consistent with the ocean loading model for the Caribbean subduction zone proposed by Austermann *et al.* (2013). In their model, they suggest that ocean loading at Barbados produces less viscous deformation because of the high viscosity of the subducting South American Plate, while adjacent regions underlain by weaker viscosities undergo greater viscous deformation. At greater depths (e.g. 1200 km), we observe significant and complex changes to the structure of the viscosity sensitivity kernel for 1-D versus 3-D viscosity models. For example, we observe a switch from negative to positive kernel values beneath the eastern coast of North America, which may be related to presence of the Farallon slab. Although the origin of these changes is not always obvious, they demonstrate the importance of 3-D viscosity structure in modulating which regions of the Earth an observation is sensitive to.

Finally, it is worth noting that many regions of the mantle are not characterized by strong viscosity heterogeneity, but rather small perturbations about the mean mantle viscosity (Figs 2 and S2). These regions generally exhibit more limited changes in first-order structure of the viscosity kernel (e.g. 300 km depth; Fig. S18), even in cases where stronger viscosity heterogeneity exists at nearby depths (e.g. 150 km depth; Fig. S18), although there are important exceptions to this general rule.

## 7 CONCLUSIONS

In part one of our efforts to lay out a robust framework for imaging 3-D mantle viscosity using palaeo sea-level observations, we have reviewed the conceptual description of Fréchet derivatives and how to calculate them for viscosity and initial sea level in the GIA problem. Furthermore, a review of the rate formulation of the forward and adjoint GIA problem as derived by Al-Attar & Tromp (2013) and Crawford *et al.* (2018) is provided in Appendix A. We have extended this work to calculate sensitivity kernels for observations of relative sea level and, in the process, have demonstrated that their adjoint loads are composed of equal but opposite sea-level adjoint loads at *t*_obs_ and *t_p_*. Moreover, we have shown that these kernels can also be determined by differencing the sensitivity kernels for absolute sea-level observations at *t*_obs_ and *t_p_*. Although we focus on viscosity sensitivity kernels, the approach can also be used to calculate sensitivity kernels for other model parameters, such as the rate of change in ice thickness.

We have also presented an extension to the numerical implementation of the forward and adjoint GIA problem that allows for the inclusion of 3-D viscosity, which is a fundamental requirement for 3-D imaging. In order to apply this extension sensibly, a new inference of 3-D mantle viscosity based on the shear-wave speed of GLAD-M25 (Bozdağ *et al.*^2016^; Lei *et al.*^2020^) has been produced by roughly following the approach of Austermann *et al.* (2021). Care has been taken during its construction to allow the entire mantle and crust to be viscoelastic. Through this choice, we naturally include lateral variations in lithospheric viscosity and thickness, thereby permitting characteristics of the surface load changes to determine the extent of elastic versus viscous deformation. This new 3-D viscosity inference is included within the Supporting Information.

We have demonstrated how to use the adjoint method to determine the initial sea level of the simulation, such that for any combination of Earth structure, rheology, and ice history, forward GIA simulations accurately arrive at the observed present-day topography. In order to minimize numerical artefacts due to truncation of the underlying spherical harmonic basis functions, we have shown the importance of a two-step inversion strategy that initially focuses on fitting long-wavelength observations before adding in shorter wavelength features. This same strategy can be effective in avoiding local minima and has been successfully used in seismic tomography based on the adjoint method (e.g. Pratt 1999; Fichtner *et al.*^2009^; Zhu *et al.*^2015^). Although a similar iterative approach to this problem is routine (e.g. Kendall *et al.*^2005^), our procedure permits simultaneous inversion for initial sea level and other model parameters (e.g. mantle viscosity, ice thickness changes).

Using a 1-D Earth structure, we have provided and discussed the characteristics of viscosity sensitivity kernels for both absolute and relative sea-level observations that are located in near-field, forebulge and far-field settings. Through these examples, we gain intuition concerning how physical processes are encoded within the structure of the kernel. For example, we have explored how the geometry of solid Earth rebound and forebulge collapse influences sea level. We have observed how sea-level observations are influenced by continental levering, by ocean siphoning and expulsion, as well as by coupling of weaker viscosity upper mantle with stiffer viscosity lower mantle. We acknowledge that identification of these processes can be challenging, but doing so provides deeper insight into the behaviour of sea level at a particular location and can improve the design of forward modeling experiments.

Although there are many differences amongst the viscosity sensitivity kernels for observations of absolute sea level and relative sea level, there are four general characteristics that are worth reiterating. First, kernels for near-field observations have amplitudes that are ∼10–100 times greater than those that are located on the forebulge or within the far field. Secondly, the sensitivities for near-field observations are dominated by the closest regions of surface mass change. In contrast, kernels for far-field observations have similar amplitude sensitivities both locally and beneath regions of major surface mass change (e.g. Laurentide ice sheet). This last point conveniently demonstrates why estimates of 1-D mantle viscosity based on far-field observations may be biased. Third, far-field viscosity sensitivity kernels fall into two groups that can be differentiated based on whether the region intermediate to the observation site and ice-mass change is dominantly continental or oceanic in nature. For the former, the structure of the kernel is more diffuse, while in the latter, a linear and higher amplitude zone of sensitivity develops that is reminiscent of *banana–doughnut* kernels in seismology (Dahlen *et al.*^2000^). Finally, observations of absolute sea level and relative sea level are uniquely sensitive to viscosity in the deep mantle and the amplitude of their 3-D sensitivity kernels are non-negligible, in contrast to what has previously been suggested by 1-D sensitivity kernels (e.g. Mitrovica & Peltier 1991b; Lau *et al.*^2016^).

Finally, for the first time, we have presented global 3-D viscosity sensitivity kernels for both absolute and relative sea-level observations that are calculated for a 3-D viscosity model. In general terms, inclusion of 3-D viscosity structure leads to greater complexity of the kernels. Using examples from the Amundsen Sea Embayment and Barbados, we have demonstrated that including lower viscosity regions introduces higher amplitude and shorter wavelength structure into the kernel. In high-viscosity regions, the inverse is true and there is a threshold above which elastic deformation dominates and the viscosity sensitivity kernel tends to zero. This latter effect leads to the greatest sensitivities being concentrated in a lower viscosity region that can be quite distal to the observation site. These examples begin to reveal the non-linear behaviour of the viscosity Fréchet derivatives and hence, indicate the highly non-linear nature of an inversion for 3-D mantle viscosity. It is this inversion that we will focus on in a companion study (Lloyd *et al.* in preparation), where we will use the tools and intuition developed herein to develop strategies for inverting synthetic palaeo sea-level observations in order to image a target 3-D mantle viscosity model.

## Supplementary Material

ggad455_Supplemental_Files **Figure S1**. Voigt average shear-wave speeds from GLAD-M25. Depth slices through the Voigt average shear-wave speed anomalies of GLAD-M25 (Bozdağ *et al.*^2016^; Lei *et al.*^2020^). Wave speed anomalies are plotted 1-D radial average of GLAD-M25.
**Figure S2**. Inferred viscosity structure based on GLAD-M25. Depth slices of the 3-D viscosity model inferred from the shear-wave speeds structure of GLAD-M25 (Fig. S1; Bozdağ *et al.*^2016^; Lei *et al.*^2020^). Viscosity anomalies are relative to the 1-D radial viscosity model discussed in Section 5.1 and shown in Fig. S4.
**Figure S3**. Depth to the 1175 °C isotherm. Map showing the depth to the 1175 °C isotherm in the intermediate temperature inference based on the shear-wave speeds of GLAD-M25 (Bozdağ *et al.*^2016^; Lei *et al.*^2020^). The 3-D temperature inference is provided in the Supporting Information.
**Figure S4**. Distribution of the inferred 3-D viscosity structure and a comparison with our 1-D viscosity model. Plot of our 1-D radial viscosity model (cyan line), which from the surface to the core–mantle boundary has viscosities of ∼1.8 *×* 10^26^, 5 × 10^20^ and 5 × 10^21^ Pa·s with discontinuities at 100 and 670 km depth. In the background is a globally normalized 2-D density heatmap of the inferred 3-D viscosity structure (Fig. S2). When computing the normalized density for each spherical shell, each viscosity element is weighted by the sin  of its colatitude in order to account for the change in element density along each line of latitude. In addition, the cyan dotted line indicates the minimum and maximum of the 3-D viscosity model as a function of depth.
**Figure S5**. Comparison of viscosity sensitivity kernels for sea-level and relative sea-level observations in the Amundsen Sea Embayment for a 1-D viscosity structure. Slices at 75, 150 and 300 km depth through the viscosity sensitivity kernels for (top row) a sea-level observation at 10 ka, (middle row) a sea-level observation at 0 ka, and (bottom row) a relative sea-level measurement at 10 ka. The inset map, centred on the observation site (cyan circle), has a width of 30° and it is extent is shown by the red dashed line the main map. The colour scale for each column is chosen to symmetrically span the full range of relative sea-level viscosity sensitivity kernel and thus, regions of the sea-level sensitivity kernels may be saturated.
**Figure S6**. Comparison of viscosity sensitivity kernels for sea-level and relative sea-level observations in the Amundsen Sea Embayment for a 1-D viscosity structure. Panels are the same as Fig. S5, but for slices at 600, 1200 and 2400 km depth.
**Figure S7**. Comparison of viscosity sensitivity kernels for sea-level and relative sea-level observations in the Amundsen Sea Embayment for a 3-D viscosity structure. Panels are the same as Fig. S5, but now we have used our filtered and bounded 3-D viscosity inference (Fig. 2).
**Figure S8**. Comparison of viscosity sensitivity kernels for sea-level and relative sea-level observations in the Amundsen Sea Embayment for a 3-D viscosity structure. Panels are the same as Fig. S7, but for slices at 600, 1200 and 2400 km depth.
**Figure S9**. Comparison of viscosity sensitivity kernels for sea-level and relative sea-level observations at Barbados for a 1-D viscosity structure. Panels are the same as Fig. S5, but now the observation site is at Barbados and the width of inset map is 20°.
**Figure S10**. Comparison of viscosity sensitivity kernels for sea-level and relative sea-level observations at Barbados for a 1-D viscosity structure. Panels are the same as Fig. S9, but for slices at 600, 1200 and 2400 km depth.
**Figure S11**. Comparison of viscosity sensitivity kernels for sea-level and relative sea-level observations at Barbados for a 3-D viscosity structure. Panels are the same as Fig. S5, but now we have used our filtered and bounded 3-D viscosity inference (Fig. 2) and the width of inset map is 20°.
**Figure S12**. Comparison of viscosity sensitivity kernels for sea-level and relative sea-level observations at Barbados for a 3-D viscosity structure. Panels are the same as Fig. S11, but for slices at 600, 1200 and 2400 km depth.
**Figure S13**. Comparison of viscosity sensitivity kernels for sea-level and relative sea-level observations at Andenes,Norway for a 1-D viscosity structure. Panels are the same as Fig. S5, but now the observation site is at Andenes, Norway and the width of inset map is 30°.
**Figure S14**. Comparison of viscosity sensitivity kernels for sea-level and relative sea-level observations at Andenes, Norway for a 1-D viscosity structure. Panels are the same as Fig. S13, but for slices at 600, 1200 and 2400 km depth.
**Figure S15**. Profile A–A’ Comparison of viscosity sensitivity kernels for sea-level and relative sea-level observations at Andenes, Norway for a 1-D viscosity structure. A radial slice along profile A–A’ through the viscosity sensitivity kernels for (*a*) an absolute sea-level observation at 10 ka,(*b*) an absolute sea-level observation at 0 ka and (*c*) a relative sea-level measurement at 10 ka. The location of this profile is shown on the bottom centre map, which is centred on the observations site (cyan circle). In the radial cross-sections the black dashed line shows the 670 km discontinuity. Above this discontinuity the values of the kernel correspond to the colour scale in the lower-left corner. Those values in the lower mantle are coloured using the colour scale in the lower-right corner. The colour scales are chosen to symmetrically span the full range of relative sea-level viscosity sensitivity kernel in these two regions and thus, regions of the sea-level sensitivity kernels may be saturated.
**Figure S16**. Comparison of viscosity sensitivity kernels for sea-level and relative sea-level observations at Andenes, Norway for a 3-D viscosity structure. Panels are the same as Fig. S5, but now we have used our filtered and bounded 3-D viscosity inference (Fig. 2) and the width of inset map is 30°.
**Figure S17**. Comparison of viscosity sensitivity kernels for sea-level and relative sea-level observations at Andenes, Norway for a 3-D viscosity structure. Panels are the same as Fig. S16, but for slices at 600, 1200 and 2400 km depth.
**Figure S18**. Relative sea-level viscosity sensitivity kernels for 1-D and 3-D viscosity structure. Slices at 150 and 300 km depth through the viscosity sensitivity kernels for a relative sea-level observation on at Andenes, Norway (cyan circle) dating to 10 ka. The first column shows the sensitivity kernel obtained when assuming our 1-D viscosity model (Section 5.1 and Fig. S4) and the second column shows the sensitivity kernel obtained when assuming our filtered and bounded 3-D viscosity inference (Fig. 2). It is this 3-D viscosity structure that is shown in the third column and regions where the amplitude of the sensitivity kernel are less than 0.1 per cent of the maximum amplitude of the kernel are shaded in grey. The inset map is centred on Andenes and has a width of 30°.
Please note: Oxford University Press is not responsible for the content or functionality of any supporting materials supplied by the authors. Any queries (other than missing material) should be directed to the corresponding author for the paper.

## Figures and Tables

**Figure C1.**
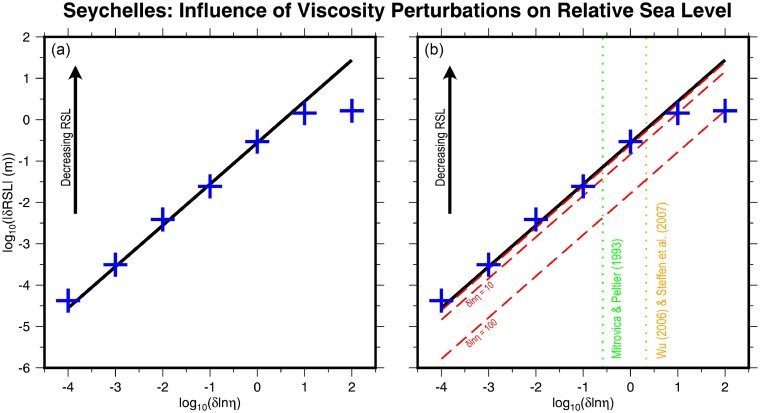
Comparison of the finite difference and adjoint approaches to calculating Fréchet derivatives. Results show the magnitude of the relative sea-level change as a function of viscosity perturbation for an observation covering the period 10–0 ka from the Seychelles. (a) The solid black line is determined by eqs (C6) and (C7) along with the sensitivity kernel, *K*_ln *η*_ obtained from the adjoint method (Figs 10g–i). The slope of this line is the partial derivative, $\frac{\partial RSL}{\partial \ln \eta _{i}}$, obtained from eq. (C6) and corresponds to the global sublithospheric upper mantle voxel. The plus symbols show the change in relative sea level obtained from a forward GIA simulation that adopts the corresponding perturbed viscosity model. (b) The right-hand panel is the same, but with the addition of three red dashed lines determined using the finite difference method (eq. C4) for viscosity perturbations of 0.1, 10 and 100 (left to right). The vertical dotted lines show the viscosity perturbation adopted by various other studies that have used the finite difference method to calculate Fréchet derivatives (Mitrovica & Peltier 1993; Wu 2006; Steffen *et al.*^2007^).

## Data Availability

The 3-D viscosity inference based on the shear-wave speeds of GLAD-M25 (Lei *et al.*^2020^; Bozdağ *et al.*^2016^) is included within the Supporitng Information.

## APPENDIX A: REVIEW OF THE RATE FORMULATION OF THE FORWARD AND ADJOINT GIA PROBLEM

### A1 The forward GIA problem

The approach taken here to solve the GIA problem differs from methods that rely on iteratively solving the sea level equation (e.g. Mitrovica & Milne 2003; Kendall *et al.*^2005^). Instead, Al-Attar & Tromp (2013) and Crawford *et al.* (2018) derive coupled evolution equations that embody the same physics as the sea-level equation, but can be solved numerically with an explicit time-stepping scheme and are ideally suited for the adjoint method. Consistent with other solutions of the GIA problem, the solid Earth is assumed to undergo quasi-static deformation, to be self-gravitating, and to be initially at rest and in hydrostatic equilibrium. Previously, it has been assumed to be spherical, isotropic, compressible and composed of an elastic inner core, an inviscid fluid outer core, a viscoelastic mantle, and an elastic lithosphere. In our simulations, however, we allow the mantle and crust to deform viscoelastically and allow the lithosphere to be defined by the extent of high-viscosity regions and the characteristics of the load changes (Section 4). In addition, we assume that deformation in viscoelastic regions is governed by a Maxwell rheology and neglect bulk viscosity, which are both common assumptions in GIA studies (Whitehouse 2018), although it is worth noting that transient linear and non-linear rheologies can also be implemented within our strategy (as discussed in Crawford *et al.*^2016^).

Crawford *et al.* (2018) extended the work of Al-Attar & Tromp (2013) to include gravitationally self-consistent sea-level change with shoreline migration. This extension is achieved by assuming that the oceans and ice sheets are sufficiently thin such that they can be represented as surface loads. Strictly speaking, the inclusion of ice sheets, non-global oceans and continents violates the model’s initial condition of hydrostatic equilibrium. Nevertheless, the expected departure from a hydrostatic pre-stessed field due to realistic lateral heterogeneity will be small, and so additional terms associated with the deviatoric pre-stress are neglected (Dahlen & Tromp 1999). It is also assumed that the oceans remain in hydrostatic equilibrium and are interconnected, thus requiring their surface to lie along the same gravitational equipotential. Under the assumption that water mass is conserved between the oceans and ice sheets, the rate of sea-level change is

(A1)
\begin{eqnarray*}
\dot{SL} = -\frac{1}{g}(\dot{\mathbf {u}} \cdot \nabla \Phi + \dot{\phi }) + \frac{1}{gA} \int _{\partial M} C(\dot{\mathbf {u}} \cdot \nabla \Phi + \dot{\phi }) \, \mathrm{d}S - \frac{\rho _i}{\rho _wA} \int _{\partial M} (1-C)\dot{I} \, \mathrm{d}S,
\end{eqnarray*}

where dots are used to denote time derivatives and the variables are defined in Table A1. The reduced weak form of the forward GIA problem with gravitationally self-consistent sea-level change and shoreline migration is

(A2)
\begin{eqnarray*}
\mathcal {A}\left(\dot{\mathbf {u}},\dot{\phi }|\mathbf {u^{\prime }},\phi ^{\prime }\right)& -& \frac{\rho _w}{g} \int _{\partial M} \left[ \dot{\mathbf {u}} \cdot \nabla \Phi + \dot{\phi } - \frac{1}{A} \int _{\partial M} C(\dot{\mathbf {u}} \cdot \nabla \Phi + \dot{\phi }) \, \mathrm{d}S \right] C(\mathbf {u}^{\prime } \cdot \nabla \Phi + \phi ^{\prime }) \, \mathrm{d}S \\
&=& \int _{M_S} 2\mu _{0} \left[ \dot{\mathbf {m}} :\mathbf {m}^{\prime } + \frac{1}{\tau } (\mathbf {d}-\mathbf {m}) :(\mathbf {d}^{\prime }-\mathbf {m}^{\prime }) \right] \, \mathrm{d}V \\
&-& \rho _i \int _{\partial M} (1-C)\dot{I}_c \left[ \mathbf {u}^{\prime } \cdot \nabla \Phi + \phi ^{\prime } - \frac{1}{A} \int _{\partial M} C(\mathbf {u}^{\prime } \cdot \nabla \Phi + \phi ^{\prime }) \, \mathrm{d}S \right] \, \mathrm{d}S.
\end{eqnarray*}

Within this equation, **m** is an *internal variable* used to simulate a Maxwell rheology, with the evolution of this term governed by

(A3)
\begin{eqnarray*}
\dot{\mathbf {m}} + \frac{1}{\tau }(\mathbf {m}-\mathbf {d}) = \mathbf {0}.
\end{eqnarray*}

Here, the *internal variable***m** contains the memory of past deformation, in which the duration of this memory is controlled by the *Maxwell relaxation time, τ*, and is by extension related to viscosity, *η*. The derivation of these equations can be found in Al-Attar & Tromp (2013) and Crawford *et al.* (2018). We now focus on a few key aspects of eqs (A1) and (A2).

**Table A1.** Variables used in the forward and adjoint formalism.

Variable	Meaning	Variable	Meaning
*Mathematical symbols, superscripts, subscripts*			
$\dot{*}$	Time derivative	∇	Gradient
*′	Test function	*^†^	Adjoint variable
·	Dot product	:	Contraction
*M_S_*	Union of the solid regions	$\, \mathrm{d}V$	Volume integral
∂*M*	Surface	$\, \mathrm{d}S$	Surface integral
*Time parameters*			
*t*	Forward time	*t* ^†^	Adjoint time (*t*^†^ = *t*_1_ − *t* + *t*_0_)
*t*_0_	Initial time (e.g. 26 ka)	*t*_1_	Final time (e.g. 0 ka)
*Ice-sheet parameters*			
*I*	Ice thickness	*ρ*_i_	Density of ice
*I*_0_	Initial ice thickness	*I*_c_	Current ice thickness
*Sea-level parameters*			
*SL*	Sea level	*ρ*_w_	Density of water
*C*	Ocean mask	*A*	Area of Ocean
*SL*_0_	Initial sea level		
*Solid-Earth parameters*			
**u**	Displacement	*ϕ*	Gravitational potential perturbation
Φ	Gravitational potential of the reference model	*g*	Magnitude of gravitational acceleration
μ_0_	Unrelaxed shear modulus	$\mathcal {A}$	Bilinear form associated with elasto-gravitational forces
*η*	Viscosity	τ	*Maxwell relaxation time*
**m**	*Internal memory variable*	**d**	Deviatoric strain tensor
$\boldsymbol{\tau }$	Deviatoric Stress		
*Select adjoint parameters*			
$\dot{h}_{SL,\mathbf {u},\phi }$	Adjoint loads: sea level, displacement, gravitation perturbation	$K_{\eta ,SL_{0}}$	Sensitivity kernel: viscosity, initial sea level

First, and foremost, eqs (A1) and (A2) are a non-iterative formulation for gravitationally self-consistent sea-level change with shoreline migration. The non-linearity of shoreline migration, due to the interplay of ocean height and solid Earth deformation, is captured through dependence of the ocean function, *C*, on the ocean height, *SL* and the ice thickness, *I*. Note that this rate formulation does not yet include rotation, which is the subject of ongoing work. It is our expectation that rotation will have a minor and long-wavelength effect on the viscosity sensitivity kernels because it is composed primarily of a spherical harmonic degree-two and order-one signal (e.g. Han & Wahr 1989; Milne & Mitrovica 1998). In addition, rotation is not required to develop an adjoint-based recalibration scheme for initial sea level nor is it required to initially explore inversion strategies for imaging 3-D mantle viscosity (Lloyd *et al.* in prepration).

Eq. (A2) forms the core of the forward GIA problem and consists of the elasto-gravitational terms within $\mathcal {A}(\dot{\mathbf {u}},\dot{\phi }|\mathbf {u^{\prime }},\phi ^{\prime })$, the surface-load changes due to the ice sheets and ocean are manifested within the second and fourth integral terms, and the viscous response of the system within the third integral term. As written, all terms containing the unknown deformation field components, $\left\lbrace \dot{\mathbf {u}},\dot{\phi }\right\rbrace$, are on the left-hand side and the right-hand side contains the integral terms that are readily calculated or known. Since these terms are linear with respect to $\dot{\mathbf {u}}$ and $\dot{\phi }$, eq. (A2) has the schematic form $\mathbf {A}\dot{\mathbf {x}}=\mathbf {b}$, which means that the time derivatives of the deformation fields can be obtained by solving a set of linear equations. Finally, time derivatives of sea level and the *internal variables* can be directly calculated from their stated evolution equations and, in this manner, the whole system can be time-stepped.

### A2 The adjoint GIA problem

Analagous to the previous subsection, we do not review the full derivation of adjoint GIA equations presented in Crawford *et al.* (2018), but rather go over the essential ideas behind their derivation in a schematic manner. We then state the form of the adjoint equations and briefly discuss their structure, as well as explaining how the relevant sensitivity kernels are derived.

Let the vector, *U*, denote the state of the physical system and a vector, *P*, the underlying model parameters. The state vector *U* is defined over an interval *t* ∈ [*t*_0_, *t*_1_], while *P* may also have an explicit time dependence. We suppose that the forward problem governing the physics is posed as an initial-value problem

(A4)
\begin{eqnarray*}
\dot{U} - g(U,P) - \mathcal {F} = 0, \quad U(t_0) = U_0,
\end{eqnarray*}

where *g* is a given function of *U* and *P*, while $\mathcal {F}$ describes the forcing of the system. This equation is assumed to have a unique solution, *U*, for any appropriate value of *P*.

We also consider a scalar-valued *objective function, F*(*U, P*), which could be an observation (e.g. sea level at a specific location and time) or the misfit between predictions and observations. The explicit dependence of *F* on the model vector *P* would, in practice, be due to regularization terms within the misfit, which might seek to dampen or smooth the solution. By solving the forward problem, the state of the system *U* becomes an implicit function of the model parameters *P*, the initial state *U*_0_ and the system forcing $\mathcal {F}$ and can be written as $U = \hat{U}(P,U_{0},\mathcal {F})$. The corresponding value of *F* then depends on *P* and *U*_0_ alone and, to illustrate this fact, we define the *reduced* functional

(A5)
\begin{eqnarray*}
\hat{F}(P,U_{0}) = F\left[\hat{U}(P,U_{0},\mathcal {F}),P\right].
\end{eqnarray*}

Our goal is to differentiate the function, $\hat{F}$, with respect to the model parameters, *P* and initial conditions, *U*_0_. This procedure is equivalent to differentiating *F*(*U, P*) with respect to *P*, subject to the constraint that *U* satisfies the stated initial value problem. To achieve this task, we apply the method of Lagrange multipliers and so introduce the *Lagrangian*

(A6)
\begin{eqnarray*}
\mathcal {L}(U,U^{\prime },P,U_{0},U_{0}^{\prime }) \equiv F(U,P) - \int _{t_{0}}^{t_{1}} \left\langle \dot{U} - g(U,P) - \mathcal {F} \, , \, U^{\prime } \right\rangle \, \mathrm{d}t - \left\langle U(t_{0}) - U_{0} \, , \, U_{0}^{\prime } \right\rangle .
\end{eqnarray*}

Here, $\left\langle \cdot \, ,\, \cdot \right\rangle$ denotes an appropriate inner product for state vectors, *U*′ is a time-dependent Langrange multiplier associated with the differential equation for *U*, and $U_{0}^{\prime }$ is a time-independent Lagrange multiplier linked to the initial conditions. The Lagrange multiplier theorem states that

(A7)
\begin{eqnarray*}
D_{P} \hat{F}(P,U_{0}) &=& D_{P}L(U,U^{\prime },P,U_{0},U_{0}^{\prime }), \\
D_{U_{0}} \hat{F}(P,U_{0}) &=& D_{U_{0}}L(U,U^{\prime },P,U_{0},U_{0}^{\prime }), \\
D_{\mathcal {F}} \hat{F}(P,U_{0}) &=& D_{\mathcal {F}}L(U,U^{\prime },P,U_{0},U_{0}^{\prime }),
\end{eqnarray*}

subject to the following conditions holding:

(A8)
\begin{eqnarray*}
D_{U^{\prime }}L(U,U^{\prime },P,U_{0},U_{0}^{\prime }) = 0, \quad D_{U^{\prime }_{0}}L(U,U^{\prime },P,U_{0},U_{0}^{\prime }) = 0, \quad D_{U}L(U,U^{\prime },P,U_{0},U_{0}^{\prime }) = 0.
\end{eqnarray*}

The first two conditions, stating that *L* is stationary with respect to the Lagrange multipliers, simply require that the state vector solves the given initial value problem. The final condition, however, gives rise to new equations that must be satisfied by the Lagrange multipliers. To demonstrate this aspect, we note that for any variation δ*U* to the state vector, we must have

(A9)
\begin{eqnarray*}
\int _{t_{0}}^{t_{1}} \left\langle \delta U \, , \, \mathcal {H}^{\prime } \right\rangle \, \mathrm{d}t + \int _{t_{0}}^{t_{1}} \left\langle \delta U \, , \, \dot{U}^{\prime } + \left[D_{U}g(U,P)\right]^{*} U^{\prime } \right\rangle \, \mathrm{d}t + \left\langle \delta U (t_{0}) \, , \, U^{\prime }(t_{0}) + U^{\prime }_{0}) \right\rangle - \left\langle \delta U(t_{1}) \, , \, U^{\prime }(t_{1}) \right\rangle = 0,
\end{eqnarray*}

where the first term involving $\mathcal {H}^{\prime }$ arises through variation of *F* with respect to *U*. Note that, to isolate δ*U* within the second integral, an integration by parts has been performed and the definition of the adjoint (indicated by the superscript *) of a linear operator has been applied. In order for this relationship to hold for arbitrary *δU*, we see that *U*′ must satisfy the following differential equation

(A10)
\begin{eqnarray*}
\dot{U}^{\prime } + \left[D_{U}g(U,P)\right]^{*} U^{\prime } + \mathcal {H}^{\prime } = 0,
\end{eqnarray*}

subject to the terminal condition *U*′(*t*_1_) = 0, while we also have $U^{\prime }_{0} = U^{\prime }(t_{0})$.

As it is more usual to work with initial value problems, a new variable *U*^†^ can be introduced through

(A11)
\begin{eqnarray*}
U^{\dagger } = \mathbb {T} U^{\prime },
\end{eqnarray*}

where we have introduced a *time reversal operator* by

(A12)
\begin{eqnarray*}
(\mathbb {T}U)(t) = U(t_{1}-t+t_{0}).
\end{eqnarray*}

Here, we note that the terminal condition on *U*′ at *t*_1_ is mapped to an initial condition on *U*^†^ at *t*_0_. Having made this definition, we see that *U*^†^ satisfies the differential equation

(A13)
\begin{eqnarray*}
\dot{U}^{\dagger } - \mathbb {T} \left[ D_{U}g(U,P) \right]^{*} \mathbb {T} U^{\dagger } + \mathcal {H}^{\dagger } = 0 ,
\end{eqnarray*}

where equivalently $\mathcal {H}^{\dagger } = \mathbb {T} \mathcal {H}^{\prime }$. Closer examination of eq. (A13) reveals that time is reversed only for [*D_U_**g*(*U, P*)]* relative to other terms, which all share a superscript ^†^. In other words, the first appearance of $\mathbb {T}$ reverses the direction of time, while the second appearance returns the flow of time to its original direction. Similarly, the initial condition is transformed to *U*^†^(*T*_0_) = 0 and we also see that $U^{\dagger }(t_{1}) = U^{\prime }_{0}$. It is conventional to call *U*^†^ the *adjoint state vector* and the above equations the *adjoint problem*, which are driven by the *fictitious* adjoint forcing, $\mathcal {H}^{\dagger }$. Finally, we note that the structure of the adjoint problem (eq. A13) is very similar to that of the forward problem (eq. A4) and, in such instances, solutions for *U*^†^ can often be obtained using the same numerical scheme as the forward problem.

Calculation of the derivative of $\hat{F}$ with respect to *P* and/or *U*_0_ requires us to solve: (1) the forward problem for the state vector *U* and (2) the closely related adjoint problem for the adjoint state *U*^†^, with this latter problem depending on the state vector both through the adjoint force, *H*^†^, and, for non-linear forward problems, through the linear operator *D_U_**g*(*U, P*). Having solved these problems, we can use eq. (A7) to obtain the derivative of $\hat{F}$ with respect to *P, U*_0_ or $\mathcal {F}$. For example, the first-order change in $\hat{F}$ due to a perturbation δ*P* to the model parameters, *δU*_0_ to the initial conditions, and $\delta \mathcal {F}$ to the system forcing, is given by

(A14)
\begin{eqnarray*}
\delta \hat{F} = \left\langle D_{P}F(U,P) \, , \, \delta P \right\rangle + \int _{t_{0}}^{t_{1}} \left\langle [ D_{P} g(U,P)]^{*} \mathbb {T} U^{\dagger } \, , \, \delta P \right\rangle \, \mathrm{d}t + \left\langle U^{\dagger }(t_{1}) \, , \, \delta U_{0} \right\rangle . + \int _{t_{0}}^{t_{1}} \left\langle U^{\dagger } \, , \, \delta \mathcal {F} \right\rangle \, \mathrm{d}t
\end{eqnarray*}

Here, the first term on the right-hand side typically arises due to regularization and the second term contains the interaction of the forward and adjoint simulations. It is notable that the choice of objective function, *F*, enters into the adjoint problem only through the adjoint forcing $\mathcal {H}^{\dagger }$. This aspect means that minimal changes are required to apply the theory to new types of measurements or misfit functions.

Following on from this mathematical schematic, the appropriate *Lagrangian* for the GIA problem (eq. 78 of Crawford *et al.*^2018^) is given by

(A15)
\begin{eqnarray*}
\mathcal {L} &=& F - \rho _wg \int _{\partial M} \left[SL(t_0) - SL_0\right]SL^{\prime }(t_0) \, \mathrm{d}S + \rho _ig \int _{\partial M} \left[I(t_0) - I_0\right]I^{\prime }(t_0) \, \mathrm{d}S \\
&& + \int _{t_0}^{t_1} \mathcal {A}(\dot{\mathbf {u}},\dot{\phi }|\mathbf {u}^{\prime },\phi ^{\prime }) - \int _{M_S} 2\mu _{0} \left[ \dot{\mathbf {m}} :\mathbf {m}^{\prime } + \frac{1}{\tau } (\mathbf {d}-\mathbf {m}) :(\mathbf {d}^{\prime }-\mathbf {m}^{\prime }) \right] \, \mathrm{d}V - \rho _wg \int _{\partial M} \dot{SL}SL^{\prime } \, \mathrm{d}S \\
&& - \frac{\rho _w}{g} \int _{\partial M} \left[ \dot{\mathbf {u}} \cdot \nabla \Phi + \dot{\phi } - \frac{1}{A} \int _{\partial M} C(\dot{\mathbf {u}} \cdot \nabla \Phi + \dot{\phi }) \, \mathrm{d}S \right] [gSL^{\prime } + C(\mathbf {u}^{\prime } \cdot \nabla \Phi + \phi ^{\prime })] \, \mathrm{d}S - \rho _ig \int _{\partial M} (\dot{I}_c - \dot{I})I^{\prime } \, \mathrm{d}S \\
&& + \rho _i \int _{\partial M} (1-C)\dot{I}_c \left[ \mathbf {u}^{\prime } \cdot \nabla \Phi + \phi ^{\prime } - \frac{1}{A} \int _{\partial M} [gSL^{\prime }+C(\mathbf {u}^{\prime } \cdot \nabla \Phi + \phi ^{\prime })] \, \mathrm{d}S \right] \, \mathrm{d}S \, \, \mathrm{d}t,
\end{eqnarray*}

where again, descriptions of the variables can be found in Table A1. Here, the second term on the right-hand side imposes the prescribed initial sea level, *SL*_0_, the third term imposes the prescribed ice thickness, *I*_0_ and the remainder is the time integrated weak form of the forward GIA problem. Although all time-dependent variables are evaluated at time *t*, when working with the *Lagrangian*, we will find it useful to introduce the adjoint state variables (eqs A11 and A12). In order to more easily recognize their time reversal, we define the adjoint time, *t*^†^ = *t*_1_ − *t* + *t*_0_, in which the subscripts 0 and 1 are the initial and final time of the forward simulation, respectively.

Crawford *et al.* (2018) derived the adjoint equations for the GIA problem using the third condition of eq. (A8) and the introduction of the adjoint state variables (eqs A11 and A12). The resulting adjoint equations are solved for unknowns ($SL^{\dagger },\dot{\mathbf {u}}^{\dagger },\dot{\phi }^{\dagger }$), which are comparable to those in the forward equations (eqs A1 and A2), and take the form

(A16)
\begin{eqnarray*}
\dot{SL}^{\dagger } = -\frac{\dot{h}_{SL}^{\dagger }}{\rho _wg} - \frac{\dot{C}^{\dagger }}{g} \left[ \dot{\mathbf {u}}^{\dagger } \cdot \nabla \Phi + \dot{\phi }^{\dagger } - \frac{1}{A^{\dagger }} \int _{\partial M} \left[gSL^{\dagger } + C^{\dagger }(\dot{\mathbf {u}}^{\dagger } \cdot \nabla \Phi + \dot{\phi }^{\dagger })\right] \, \mathrm{d}S \right],
\end{eqnarray*}

and

(A17)
\begin{eqnarray*}
\mathcal {A}(\dot{\mathbf {u}}^{\dagger },\dot{\phi }^{\dagger }|\mathbf {u}^{\prime },\phi ^{\prime }) &-& \frac{\rho _w}{g} \int _{\partial M} \left[ \dot{\mathbf {u}}^{\dagger } \cdot \nabla \Phi + \dot{\phi }^{\dagger } - \frac{1}{A^{\dagger }} \int _{\partial M} C^{\dagger }(\dot{\mathbf {u}}^{\dagger } \cdot \nabla \Phi + \dot{\phi }^{\dagger }) \, \mathrm{d}S \right] C^{\dagger }(\mathbf {u}^{\prime } \cdot \nabla \Phi + \phi ^{\prime }) \, \mathrm{d}S \\
&=& \int _{M_S} \frac{2\mu _0}{\tau } (\mathbf {d}^{\dagger }-\mathbf {m}^{\dagger }) :\mathbf {d}^{\prime } \, \mathrm{d}V + \int _{\partial M} \left( \dot{\mathbf {h}}_{\mathbf {u}}^{\dagger } \cdot \mathbf {u}^{\prime } + \dot{h}_{\phi }^{\dagger } \cdot \phi ^{\prime } \right) \, \mathrm{d}S \\
&-& \frac{1}{g} \int _{\partial M} \dot{h}_{SL}^{\dagger } \left[\mathbf {u}^{\prime } \cdot \nabla \Phi + \phi ^{\prime } - \frac{1}{A^{\dagger }} \int _{\partial M} C^{\dagger } (\mathbf {u}^{\prime } \cdot \nabla \Phi + \phi ^{\prime }) \, \mathrm{d}S \right] \, \mathrm{d}S,
\end{eqnarray*}

respectively. The adjoint internal variable satisfies

(A18)
\begin{eqnarray*}
\dot{\mathbf {m}}^{\dagger } + \frac{1}{\tau }\left(\mathbf {m}^{\dagger }- \mathbf {d}^{\dagger }\right) = \mathbf {0},
\end{eqnarray*}

which is much like the internal variable in the forward problem (eq. A3).

A description of these variables is provided in Table A1, but we note that $\dot{h}_*^{\dagger }$ are the adjoint loads, which are equivalent to $\mathcal {H}^{\dagger }$ in the mathematical schematic. In this work, only $\dot{h}_{SL}^{\dagger }$ is non-zero because we consider only measurements directly related to sea level and not, for example, those for solid-Earth displacements or gravity in isolation. In eq. (A17), the adjoint sea-level load, $\dot{h}_{SL}^{\dagger }$, interacts with the test functions **u**′ and *ϕ*′, giving rise to two adjoint loads that act on the solid Earth and the gravitational field. Finally, we have similarly written eq. (A17) such that the unknown components of the deformation field, $\left\lbrace \dot{\mathbf {u}}^{\dagger },\dot{\phi }^{\dagger }\right\rbrace$, are on the left-hand side and the right-hand side contains the integral terms that are readily calculated or known.

Although the right-hand side of the reduced weak form of the forward and adjoint equations (eqs A2 and A17, respectively) are different, both are of the form $\mathbf {A}\dot{\mathbf {x}}=\mathbf {b}$. Thus, solutions for the adjoint deformation field rate, $\left\lbrace \dot{\mathbf {u}}^{\dagger },\dot{\phi }^{\dagger }\right\rbrace$, can be obtained using the same numerical scheme (Appendix B), but the elements of **b** will be different. In so doing, we can readily calculate the adjoint deviatoric stress, similar to the forward problem. Obtaining the adjoint sea level is, however, more challenging because eq. (A16) is potentially singular and so cannot be easily integrated. Crawford *et al.* (2018) presents a method for integrating this equation that circumvents these singularities by introducing auxiliary variables.

As we have shown in the schematic example, by solving the forward equations (eqs A1 and A2) and adjoint equations (eqs A16 and A17), we can use their results to calculate the desired derivative of $\hat{F}$ with respect to *P* (i.e. sensitivity kernel). In order to obtain the form of this derivative, we must perturb the *Lagrangian* with respect to the desired model parameter. In this study, we require expressions for the sensitivity kernels for initial sea level, *SL*_0_, and viscosity, *η*. Thus, by varying the Lagrangian in eq. (A15) with respect to *SL*_0_ and by introducing the adjoint state variables (eqs A11 and A12), we find

(A19)
\begin{eqnarray*}
\left\langle D_{SL_{0}} \hat{F},\delta SL_{0} \right\rangle = \int _{\partial M} K_{SL_{0}} \delta SL_{0} \, \mathrm{d}S,
\end{eqnarray*}

where we have defined the sensitivity kernel for initial sea level to be

(A20)
\begin{eqnarray*}
K_{SL_{0}} = \rho _wgSL_{0}^{\dagger }(t^{\dagger }_1).
\end{eqnarray*}

Note that this sensitivity kernel depends only on the adjoint sea level at the final time, $t^{\dagger }_1$, of the adjoint simulation.

Likewise, we obtain the viscosity sensitivity kernel by recalling that *τ* = *η*/*μ*_0_ for a Newtonian fluid and differentiating eq. (A15) with respect to *η* to find

(A21)
\begin{eqnarray*}
\left\langle D_{\eta }\hat{F},\delta \eta \right\rangle &= \int _{t_0}^{t_1} \int _{M_S} \frac{1}{2\eta } \boldsymbol{\tau } :\boldsymbol{\tau }^{\dagger } \delta \ln {\eta } \, \mathrm{d}V \, \mathrm{d}t
\end{eqnarray*}

where $\boldsymbol{\tau } = 2\mu _{0}(\mathbf {d}-\mathbf {m})$ is the deviatoric stress, $\boldsymbol{\tau }^{\dagger }$ is the corresponding adjoint field and $\delta \ln {\eta } = \frac{\delta \eta }{\eta }$ is a viscosity perturbation. The sensitivity kernel for a viscosity perturbation is therefore

(A22)
\begin{eqnarray*}
K_{\ln {\eta }} = \int _{t_0}^{t_1} \frac{1}{2 \eta } \boldsymbol{\tau } :\boldsymbol{\tau }^{\dagger } \, \mathrm{d}t.
\end{eqnarray*}

We see that *K*_ln *η*_ depends on the interaction between the forward and adjoint deviatoric stresses for the full duration of the simulation. Finally, we note that both sensitivity kernel equations (eqs A20 and A22) are equivalent to those determined by Al-Attar & Tromp (2013) and Crawford *et al.* (2018).

## APPENDIX B: NUMERICAL IMPLEMENTATION FOR 1-D RADIAL VISCOSITY MODELS

A detailed description of the numerical implementation of the forward and adjoint GIA equations can be found in the Appendix of Crawford *et al.* (2018), but for completeness, we briefly review how eqs (A2) and (A17) are solved at an arbitrary instant in time when adopting a 1-D radial viscosity model. In this scenario, we numerically solve eqs (A2) and (A17) by representing scalar, vector, and tensor fields within and on the solid Earth using generalized spherical harmonics to capture their angular dependence (Gelfand & Shapiro 1956; Burridge 1969), while their depth dependence is described by a 1-D radial mesh of spectral-elements that each consist of five Gauss–Lobatto–Legendre interpolation points. Using these basis functions, eqs (A2) and (A17) both decouple into spheriodal and toroidal subsystems, in which the latter are not excited by radial surface loads for a laterally homogeneous Earth. Unlike in the viscoelastic loading problem of Al-Attar & Tromp (2013), however, the two spherical harmonic components (radial and consoidal) and the gravitational perturbation do not decouple for each spherical harmonic degree-*l* and order-*m*. This behaviour occurs because the ocean load (i.e. the second integral term on the left-hand side of eqs A2 and A17) depends on the entire $\dot{\mathbf {u}}$ and $\dot{\phi }$ fields. Therefore, instead of solving the simple form

(B1)
\begin{eqnarray*}
\mathbf {A}_l\dot{\mathbf {x}}_{lm}=\mathbf {b}_{lm},
\end{eqnarray*}

for each *l*, we must solve the more complex form

(B2)
\begin{eqnarray*}
\mathbf {A}_l\dot{\mathbf {x}}_{lm} + \mathbf {g}_{lm}(\dot{\mathbf {x}})=\mathbf {b}_{lm},
\end{eqnarray*}

which requires an iterative solution (see appendix b of Crawford *et al.*^2018^). Note that the matrix **A**_*l*_ and the vectors $\dot{\mathbf {x}}_{lm}$, **g**_*lm*_, and **b**_*lm*_ are defined identically to those of eq. (14). We assume that the solution for the deformation field, $\left\lbrace \dot{\mathbf {u}},\dot{\phi }\right\rbrace$, has converged when the difference in subsequent solutions is less than $1.0\%$ of the difference between the final and initial solution. For completeness, recall that the sea-level rate, $\dot{SL}$, is obtained directly from eq. (A1) and the adjoint sea-level rate, $\dot{SL}^{\dagger }$, is obtained from eq. (A16) following a change of variables, which is described in Crawford *et al.* (2018). This setup just leaves the matter of time-stepping the forward and adjoint GIA simulations.

Inspection of eqs (A1), (A2), (A16) and (A17) reveals that the deformation field $\left\lbrace \dot{\mathbf {u}},\dot{\phi }\right\rbrace$, and sea-level rate, $\dot{SL}$, depend only on the current state of the system. Thus, an explicit time-stepping scheme is straightforward to implement and we, as in Al-Attar & Tromp (2013) and Crawford *et al.* (2018), use the second order Runge–Kutta method (Press *et al.*^1986^) and set the time step to be approximately half of the minimum *Maxwell relaxation time, τ*, which is a suitable choice for an explicit method (Bailey 2006). The inclusion of low viscosity regions (∼10^18^ Pa·s; e.g. Whitehouse *et al.*^2019^; Russo *et al.*^2022^; and Fig. S2) in these simulations, however, requires a time step much smaller than a year and gives rise to challenges both in terms of run-time and memory usage. Thus, it is clear that explicit time-stepping schemes are not ideal for simulations spanning tens of thousands of years or more and future improvements to our numerical implementation might be obtained through the use of implicit time-stepping schemes.

## APPENDIX C: VISCOSITY SENSITIVITY KERNELS FOR GIA

### C1 Approaches to computing Fréchet derivatives for GIA

Until recently, efforts to determine Fréchet derivatives (i.e. sensitivity kernels) for GIA observations relied on the finite difference method to approximate this derivative. This approach requires discretizing the solid Earth (i.e. the mantle) into *n cells* or *voxels*, whose viscosity structure can be written as a vector (ln *η*_1_, …, ln *η_n_*), where we use log-viscosities for convenience. As discussed in Section 2.1, we can define a scalar-valued functional, *F*, based on the solution of the forward problem. The value of *F* depends implicitly on the viscosity parameters, and so *F* = *F*(ln *η*_1_, …, ln *η_n_*). We can also form derivatives of *F* with respect to each of the parameters, such that the *i*th component of this discretized gradient is

(C1)
\begin{eqnarray*}
\frac{\partial F}{\partial \ln \eta _{i}}.
\end{eqnarray*}

The value of this partial derivative can be approximated using the finite-difference formula

(C2)
\begin{eqnarray*}
\frac{\partial F}{\partial \ln \eta _{i}}(\ln \eta _{1}, \dots , \ln \eta _{n}) \approx \frac{F(\ln \eta _{1}, \dots , \ln \eta _{i} + \delta \ln \eta ,\dots , \ln \eta _{n}) - F(\ln \eta _{1}, \dots , \ln \eta _{i},\dots , \ln \eta _{n})}{\delta \ln \eta }
\end{eqnarray*}

where δln *η* is a sufficiently small perturbation (i.e. step size) to the *i*th viscosity parameter for this first-order expression to be accurate. In this manner, the discretized gradient can be obtained from *n* + 1 forward calculations: one for the unperturbed model and *n* additional calculations where each model parameter is perturbed in turn.

In practice, the finite-difference approximation has been implemented for GIA in a variety of ways ranging from individual normal modes (Mitrovica & Peltier 1991a, b) to directly observable surface observations (e.g. Mitrovica & Peltier 1993, 1995; Milne *et al.*^2004^; Paulson *et al.*^2005^; Wu 2006). In these studies, *n* is chosen with computational limitations in mind and hence perturbations to the model parameters are generally applied over a voxel that is larger than the discretization of the mesh on which the simulation is performed. This necessity limits the resolution of the Fréchet derivative because the sensitivity of an observation to parameters within a subregion of the perturbed voxel cannot be isolated (Wu 2006). Furthermore, the amplitude of the model perturbation is also important, as it must be sufficiently small that the finite-difference method yields a robust approximation of the desired derivative, but not too small that numerical instabilities dominate the result. These two details (i.e. voxel size and perturbation amplitude) are therefore important considerations when using the finite difference method. By way of comparison, since the adjoint method does not require defining a voxel size or a viscosity perturbation amplitude, it negates these potential limitations and thus, the kernel’s accuracy and resolution is simply limited by the mesh resolution of the forward and adjoint GIA simulation.

Early efforts to compute viscosity Fréchet derivatives for GIA observations were methodologically limited to 1-D radial kernels that describe the influence of viscosity purely as a function of depth. The first complete kernel was calculated by Mitrovica & Peltier (1991b), who built on the work of Peltier & Andrews (1976) by providing the formalism relating viscosity to both the decay times and modal amplitudes. This approach, however, does not include the the ocean load and therefore avoids solving the sea-level equation. To circumvent this limitation, Mitrovica & Peltier (1993) devised a fully numerical approach using a finite difference approximation of the Fréchet derivative that has since been applied to compute 1-D viscosity kernels for observations of gravitational change (Mitrovica & Peltier 1993), relative sea level (Mitrovica & Peltier 1995) and three-component solid Earth deformation (Milne *et al.*^2004^). In these latter studies, kernels were constructed by perturbing viscosity by ∼−0.587 in log  space (i.e. ϵ′ = 0.1; Mitrovica & Peltier 1993) within either 22 or 32 radial layers spanning the mantle. Mitrovica & Peltier (1995) also showed that the linear approximation of the Fréchet derivatives appears to be accurate when using viscosity perturbation within a factor of 10 of the reference model, which is consistent with later results obtained from the adjoint method (Crawford *et al.*^2018^ and Appendix C2).

The transition from 1-D viscosity Fréchet derivatives that have purely radial sensitivity to 3-D kernels, which also express an observation’s sensitivity to lateral changes in viscosity, began with the work of Paulson *et al.* (2005) and ushered in a period of subtle differences in definitions within the literature that can lead to some confusion. In their study, Paulson *et al.* (2005) present the first images illuminating which regions of the mantle influence the vertical solid Earth deformation at a given location, **x′** between *t*_0_ and *t*_1_. Although their implementation is identical to the finite-difference approach, the quantity computed, ϵ, for each individual mantle voxel, **m**_*i*_ ∈ *M*, is different and is given by

(C3)
\begin{eqnarray*}
\epsilon (\mathbf {m}_{i}) = \frac{\int _{t_0}^{t_1} | u_{i}(\mathbf {x}^{\prime },t) - u_{a}(\mathbf {x}^{\prime },t)| \, \mathrm{d}t}{\int _{t_0}^{t_1} | u_{i}(\mathbf {x}^{\prime },t) | \, \mathrm{d}t}.
\end{eqnarray*}

Here, *u_i_*(*t*) is the solid Earth uplift rate at time *t* for an isoviscous reference viscosity model and *u_a_* is the uplift rate at the same time for a viscosity model that has been perturbed within that discrete mantle voxel, **m**_*i*_. Although successful in delineating a *sensitivity region*, the quantity computed is not, strictly speaking, the Fréchet derivative. *Full* 3-D Fréchet derivatives, preserving both their amplitude and polarity, were later presented by Wu (2006) for observations of relative sea level, rate of solid Earth deformation and gravitational change at discrete points in space and time. In that work and subsequent regional studies focusing on the Fennoscandian ice sheet (Steffen *et al.*^2007^; Steffen & Wu 2014), sensitivity kernels relative to a 1-D reference viscosity model were constructed using a finite difference approximation, such that

(C4)
\begin{eqnarray*}
K_{m} = \frac{\delta p}{\delta m \ \Delta V}.
\end{eqnarray*}

Here, δ*p* is the difference in the predicted observation for the perturbed versus unperturbed simulation, δ*m* is the model perturbation and Δ*V* is the voxel volume, which serves to balance variations in voxel size. In the GIA literature, the terms in the denominator of eq. (C4) are often non-dimensionalized, so that the kernel units are consistent with those of the observation. Furthermore, by comparison to eq. (C2), we see that δ*p* is equivalent to *F*(ln *η*_1_, …, ln *η_i_* + δln *η*, …, ln *η_n_*) − *F*(ln *η*_1_, …, ln *η_i_*, …, ln *η_n_*) and δ*m* is equivalent to δln *η*. The global analysis of Wu (2006) used a longitudinally symmetric ice sheet and Earth system, in which the latter is composed of 36 mantle voxels distributed as 4 layers with depth and 9 regions in colatitude. This configuration results in relatively coarse kernels that average over much of the detail found in our study and discussed in Sections 6.3 and 6.4, hindering direct comparison of sensitivities. Meanwhile, voxels in regional studies are naturally smaller but lack global coverage throughout the mantle (Steffen *et al.*^2007^; Steffen & Wu 2014). In the studies of Wu (2006) and Steffen *et al.* (2007), the viscosity within these voxels was perturbed by a factor of ∼0.332 in log  space. In Steffen & Wu (2014), however, the magnitude of each viscosity perturbation was set by the local difference between the 1-D reference viscosity model (U1L1_V1; Steffen *et al.*^2006^) and a 3-D viscosity inference of seismic tomography (U3L1_V1; Steffen *et al.*^2006^). Notably, this choice results in some voxels being perturbed by >3 orders of magnitude relative to 4 × 10^20^ Pa·s in the uppermost mantle. Such viscosity perturbations are quite large and may therefore lead to inaccuracy of the linear approximation of the Fréchet derivative in those voxels. While this issue does not detract from the goals of Steffen & Wu (2014), it leads to errors when using the Fréchet derivative to predict the change in a functional with respect to a perturbation of the viscosity structure (Appendix C2).

The studies of Wu *et al.* (2010) and others (Steffen *et al.*^2012^; Steffen & Wu 2014; Li *et al.*^2018^) use a different definition of sensitivity that results in 2-D maps. These maps are calculated for a particular time by differencing the predicted observations from two forward GIA simulations that adopt a 1-D reference viscosity model and 3-D viscosity inference. This definition is inconsistent with our study and the work described above because the resulting map: (1) is not for a single observation, but rather all surface locations combined and (2) is a 2-D field defined at the surface of the Earth as oppose to a 3-D field defined within the mantle. Most importantly, these sensitivities are not true Fréchet derivatives and are in fact equivalent to our Fig. 3(c), which shows the total sea-level change difference between simulations adopting a 3-D and 1-D viscosity structure.

### C2 Comparison of finite difference and adjoint methods

Here we compare the value and predictive accuracy of the viscosity Fréchet derivative for a single mantle voxel obtained from the finite difference and adjoint methods. To do so, we must relate the discrete derivative of eq. (C2) to its continuous counterpart. We recall from Section 2.1 that

(C5)
\begin{eqnarray*}
\delta F = \int _{M} K_{\ln \eta }\delta \ln \eta \, \mathrm{d}V,
\end{eqnarray*}

where δ*F* is the first-order change in *F* due to the viscosity perturbation, δln *η* and *K*_ln *η*_ is the viscosity Fréchet derivative. The two approaches can be linked by differentiating with respect to ln *η_i_*, yielding

(C6)
\begin{eqnarray*}
\frac{\partial F}{\partial \ln \eta _{i}} = \int _{M} K_{\ln \eta }\frac{\partial \ln \eta }{\partial \ln \eta _{i}} \, \mathrm{d}V.
\end{eqnarray*}

For our chosen parametrization, the partial derivative $\frac{\partial \ln \eta }{\partial \ln \eta _{i}}$ is equal to one within the *i*th voxel and zero elsewhere. We note that this formula is equally applicable to other model parametrizations, such as splines. Importantly, eq. (C6) indicates that the partial derivatives of *F* with respect to a given discretization can be obtained by performing suitable integrals of the continuous Fréchet derivative.

We proceed by considering a relative sea-level observation covering the period 10–0 ka from the Seychelles and a single voxel spanning the global sublithospheric upper mantle (100–600 km depth). We note, however, that any arbitrary voxel could have been chosen and that our selection was made for simplicity. In this example, the forward and adjoint GIA simulation setups are identical to those of Section 5 and they adopt our 1-D radial viscosity model [Litho (0–100 km): ∼1.5 × 10^26^ Pa·s; UM (100–670 km): 5 × 10^20^ Pa·s; LM (670–2891 km): 5 × 10^21^ Pa·s]. The resulting sensitivity kernel (Figs 10g–i) along with eq. (C6) is used to determine the change in relative sea level with respect to the viscosity perturbation of the voxel, $\frac{\partial RSL}{\partial \ln \eta _{i}}$. In the case of the finite difference approach, we perform additional forward simulations for a range of potential viscosity perturbations (δln *η_i_* ∈ [10^−4^, 10^2^]) that are applied to the global sublithospheric upper mantle voxel. These results are used to estimate the partial derivative, $\frac{\partial RSL}{\partial \ln \eta _{i}}$, based on eq. (C4). Here, δ*p* = δ*RSL* has units of meters, $\delta m = \delta \ln \eta _{i} = \frac{\delta \eta _{i}}{\eta }$ is unitless, and Δ*V* is 2.569 × 10^20^ m^3^, such that the partial derivative has units of m^−2^ and is consistent with the partial derivative obtained using the adjoint method. Finally, using the partial derivative, $\frac{\partial RSL}{\partial \ln \eta _{i}}$, obtained from the adjoint method and a subset of those estimated using the finite difference approach, we predict the change in relative sea level across the same range of viscosity perturbations by evaluating

(C7)
\begin{eqnarray*}
\delta RSL = \frac{\partial RSL}{\partial \ln \eta _{i}} \delta \ln \eta _{i}.
\end{eqnarray*}

Fig. C1 summarizes our findings for the relative sea-level observation from the Seychelles and highlights two key points. First, we see that predictions of the change in relative sea level from the adjoint method (black line) agree well with simulation results (blue pluses) when the viscosity model is modified by a perturbation of up to 0 in log  space. Beyond this threshold, the quality of the prediction rapidly worsens, confirming the non-linear dependence of relative sea level on the underlying viscosity structure. Similar predictability limits for absolute sea level are found by Crawford *et al.* (2018) and have been reported for other GIA observables (Mitrovica & Peltier 1993, 1995). This example also begins to demonstrate how if the viscosity perturbation is too small [i.e. log (δln *η_i_*) = −4] numerical instabilities may dominate the result.

Secondly, Fig. C1(b) demonstrates how the amplitude of the viscosity perturbation influences the estimated partial derivative for the same sublithospheric upper mantle voxel and, by extension, the associated predictions for the change in relative sea level (red dashed lines). We observe that for viscosity perturbations greater than 1 in log  space, the finite difference estimate of the partial derivative becomes increasingly poor, as does the predicted change in relative sea level. This result underlines the importance of choosing a reasonable viscosity perturbation and illustrates the potential pitfalls of adopting overly large values, consistent with the findings of Mitrovica & Peltier (1993) and others (e.g. Wu 2006; Steffen *et al.*^2007^). Finally, the non-linearity indicated by these results are consistent with those of Section 6.4 that also demonstrate how weak and stiff viscosity regions can significantly alter the amplitude and structure of the sensitivity kernels at regional scales.
