# Supplementary material for: GIA imaging of 3-D mantle viscosity based on palaeo sea level observations – Part I: Sensitivity kernels for an Earth with laterally varying viscosity
Source: Geophys J Int. 2023 Nov 21;236(2):1139–71. doi: 10.1093/gji/ggad455 (PMC10753356; doi:10.1093/gji/ggad455)
Supplement: ggad455_Supplemental_Files — Figure S1. Voigt average shear-wave speeds from GLAD-M25. Depth slices through the Voigt average shear-wave speed anomalies of GLAD-M25 (Bozdağ et al.2016; Lei et al.2020). Wave speed anomalies are plotted 1-D radial average of GLAD-M25. Figure S2. Inferred viscosity structure based on GLAD-M25. Depth slices of the 3-D viscosity model inferred from the shear-wave speeds structure of GLAD-M25 (Fig. S1; Bozdağ et al.2016; Lei et al.2020). Viscosity anomalies are relative to the 1-D radial viscosity model discussed in Section 5.1 and shown in Fig. S4. Figure S3. Depth to the 1175 °C isotherm. Map showing the depth to the 1175 °C isotherm in the intermediate temperature inference based on the shear-wave speeds of GLAD-M25 (Bozdağ et al.2016; Lei et al.2020). The 3-D temperature inference is provided in the Supporting Information. Figure S4. Distribution of the inferred 3-D viscosity structure and a comparison with our 1-D viscosity model. Plot of our 1-D radial viscosity model (cyan line), which from the surface to the core–mantle boundary has viscosities of ∼1.8 × 1026, 5 × 1020 and 5 × 1021 Pa·s with discontinuities at 100 and 670 km depth. In the background is a globally normalized 2-D density heatmap of the inferred 3-D viscosity structure (Fig. S2). When computing the normalized density for each spherical shell, each viscosity element is weighted by the sin of its colatitude in order to account for the change in element density along each line of latitude. In addition, the cyan dotted line indicates the minimum and maximum of the 3-D viscosity model as a function of depth. Figure S5. Comparison of viscosity sensitivity kernels for sea-level and relative sea-level observations in the Amundsen Sea Embayment for a 1-D viscosity structure. Slices at 75, 150 and 300 km depth through the viscosity sensitivity kernels for (top row) a sea-level observation at 10 ka, (middle row) a sea-level observation at 0 ka, and (bottom row) a relative sea-level measurement at 10 ka. Th [file ggad455_supplemental_files.zip › Supp_Figures.pdf]

Supplementary Material – GIA imaging of 3D mantle viscosity based on paleo sea-level observations - Part I: Sensitivity kernels for an Earth with laterally varying viscosity

Andrew J. Lloyd*[,1], Ophelia Crawford[2], David Al-Attar[2], Jacqueline Austermann[1], Mark J. Hoggard[3], Fred D. Richards[4], Frank Syvret[2]

1. Lamont Doherty Earth Observatory, Columbia University, Palisades, NY 10964, USA.
2. Bullard Laboratories, Department of Earth Sciences, University of Cambridge, Madingley Road, Cambridge CB3 0EZ, UK.
3. Research School of Earth Sciences, Australia National University, Acton, ACT 0200, Australia.
4. Department of Earth Science and Engineering, Imperial College London, London SW7 2AZ, UK.

*andrewl@ldeo.columbia.edu

3D Viscosity Inference

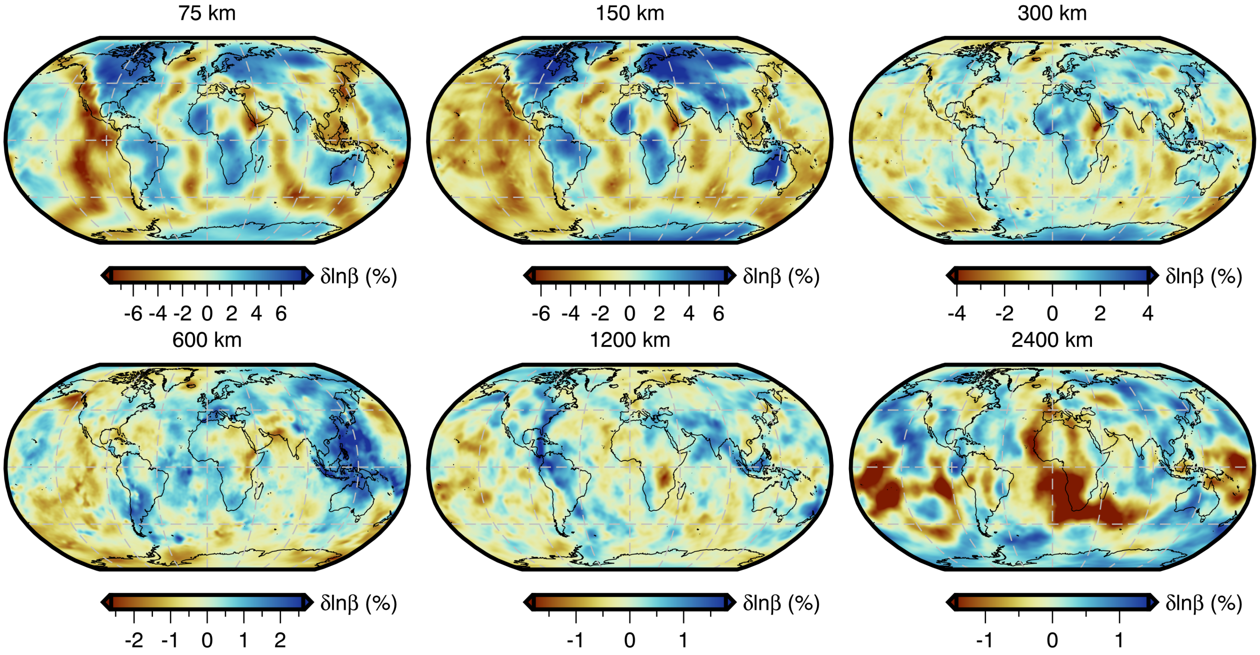

Supp. Figure S1: **Voigt average shear-wave speeds from GLAD-M25.** Depth slices through the Voigt average shear-wave speed anomalies of GLAD-M25 (Bozdağ *et al.*, 2016; Lei *et al.*, 2020). Wave speed anomalies are plotted 1D radial average of GLAD-M25.

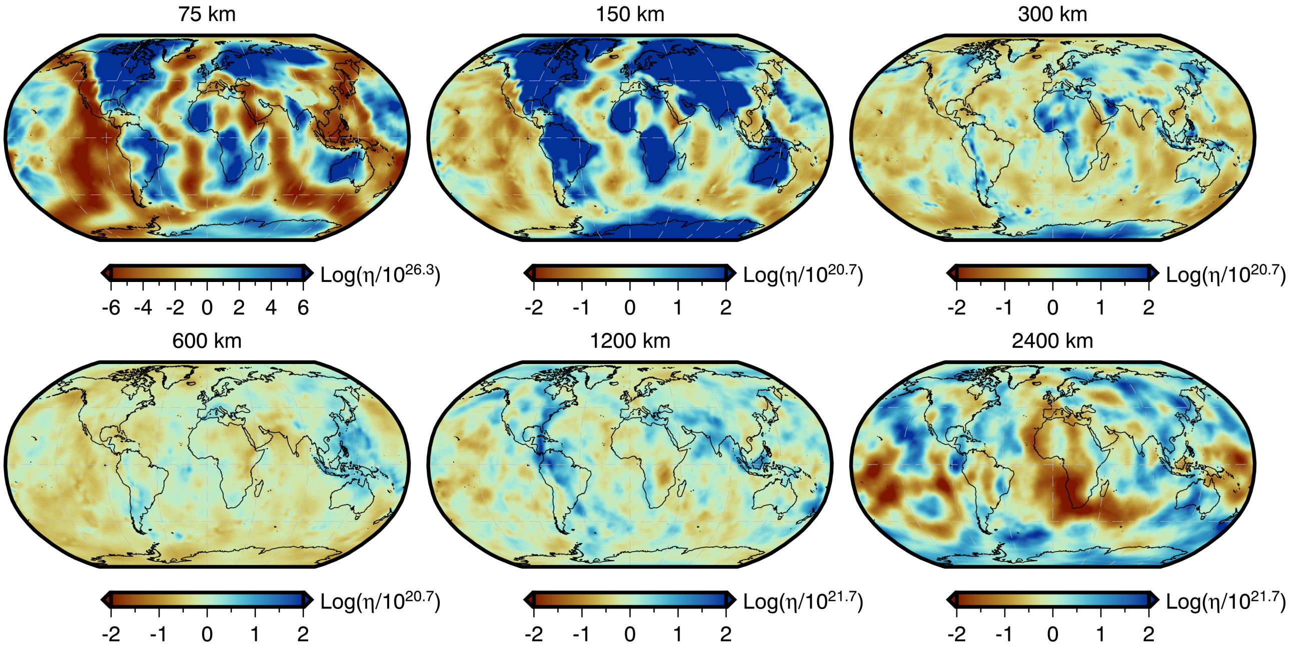

Supp. Figure S2: **Inferred viscosity structure based on GLAD-M25.** Depth slices of the 3D viscosity model inferred from the shear-wave speeds structure of GLAD-M25 (Supp. Figure S1; Bozdağ *et al.*, 2016; Lei *et al.*, 2020). Viscosity anomalies are relative to the 1D radial viscosity model discussed in Section 5.1 and shown in Supp. Figure S4.

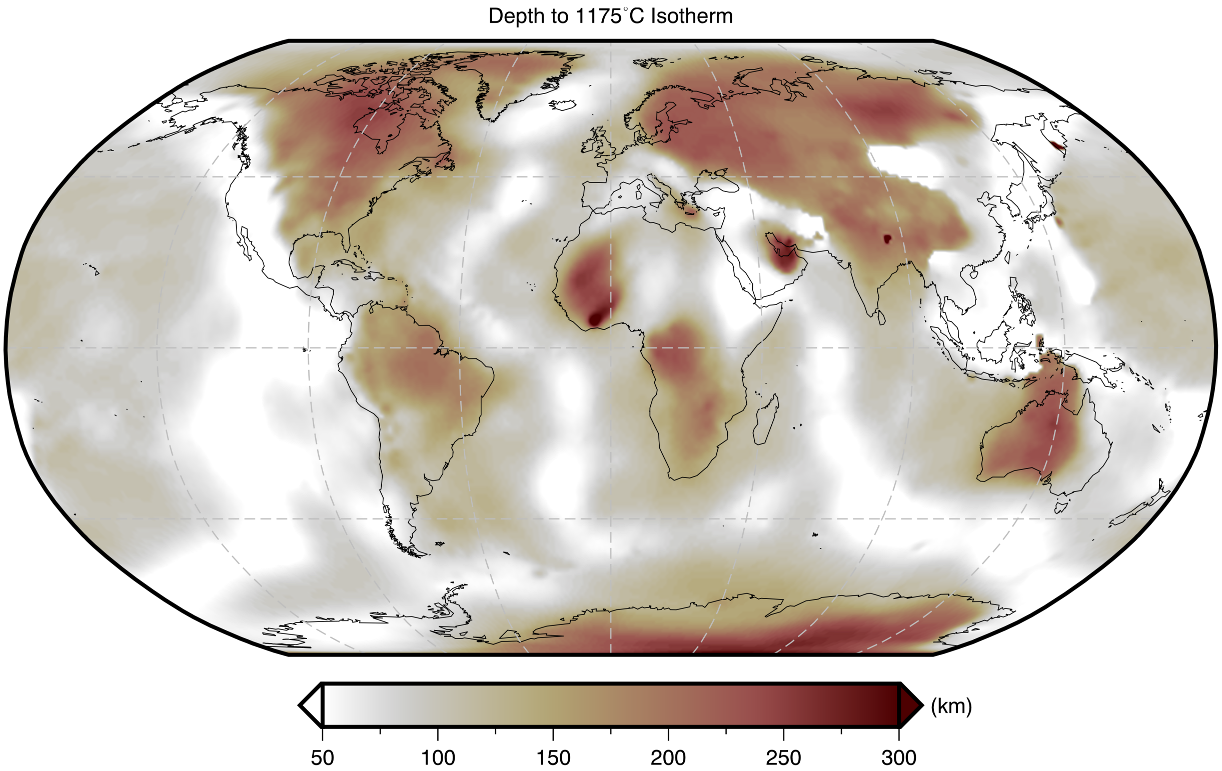

Supp. Figure S3: **Depth to the 1175°C isotherm.** Map showing the depth to the 1175°C isotherm in the intermediate temperature inference based on the shear-wave speeds of GLAD-M25 (Bozdağ *et al.*, 2016; Lei *et al.*, 2020). The 3D temperature inference is provided in the supplementary materials.

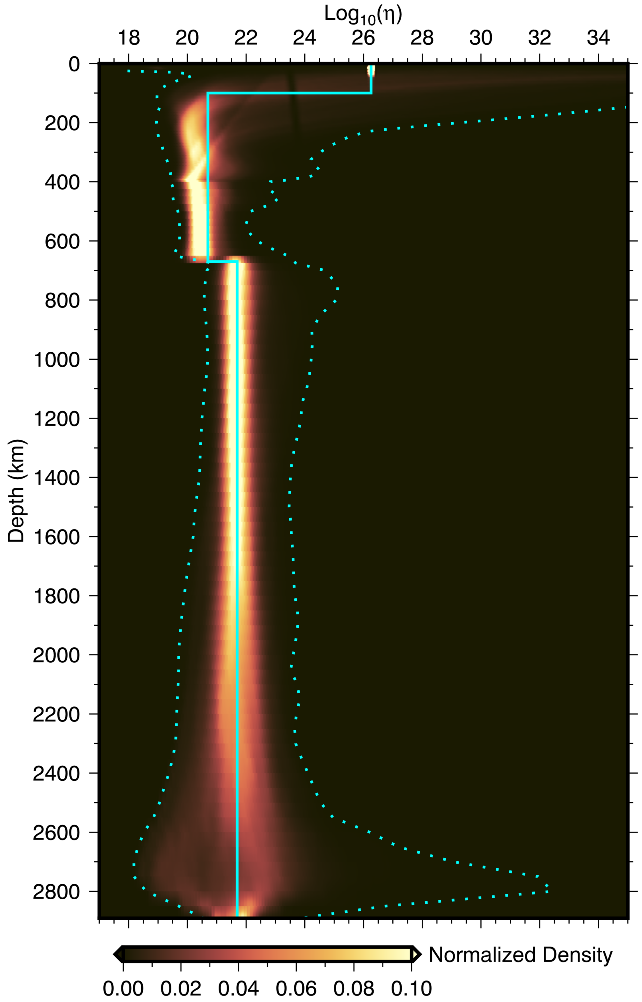

Supp. Figure S4: **Distribution of the inferred 3D viscosity structure and a comparison with our 1D Viscosity model.** Plot of our 1D radial viscosity model (cyan line), which from the surface to the core mantle boundary has viscosities of $\sim 1.8x10^{26}$, $5x10^{20}$, and $5x10^{21}$ Pa·s with discontinuities at 100 and 670 km depth. In the background is a globally normalized 2D density heatmap of the inferred 3D viscosity structure (Supp. Figure S2). When computing the normalized density for each spherical shell, each viscosity element is weighted by the sin of its co-latitude in order to account for the change in element density along each line of latitude. In addition, the cyan dotted line indicates the minimum and maximum of the 3D viscosity model as a function of depth.

Viscosity Sensitivity Kernels

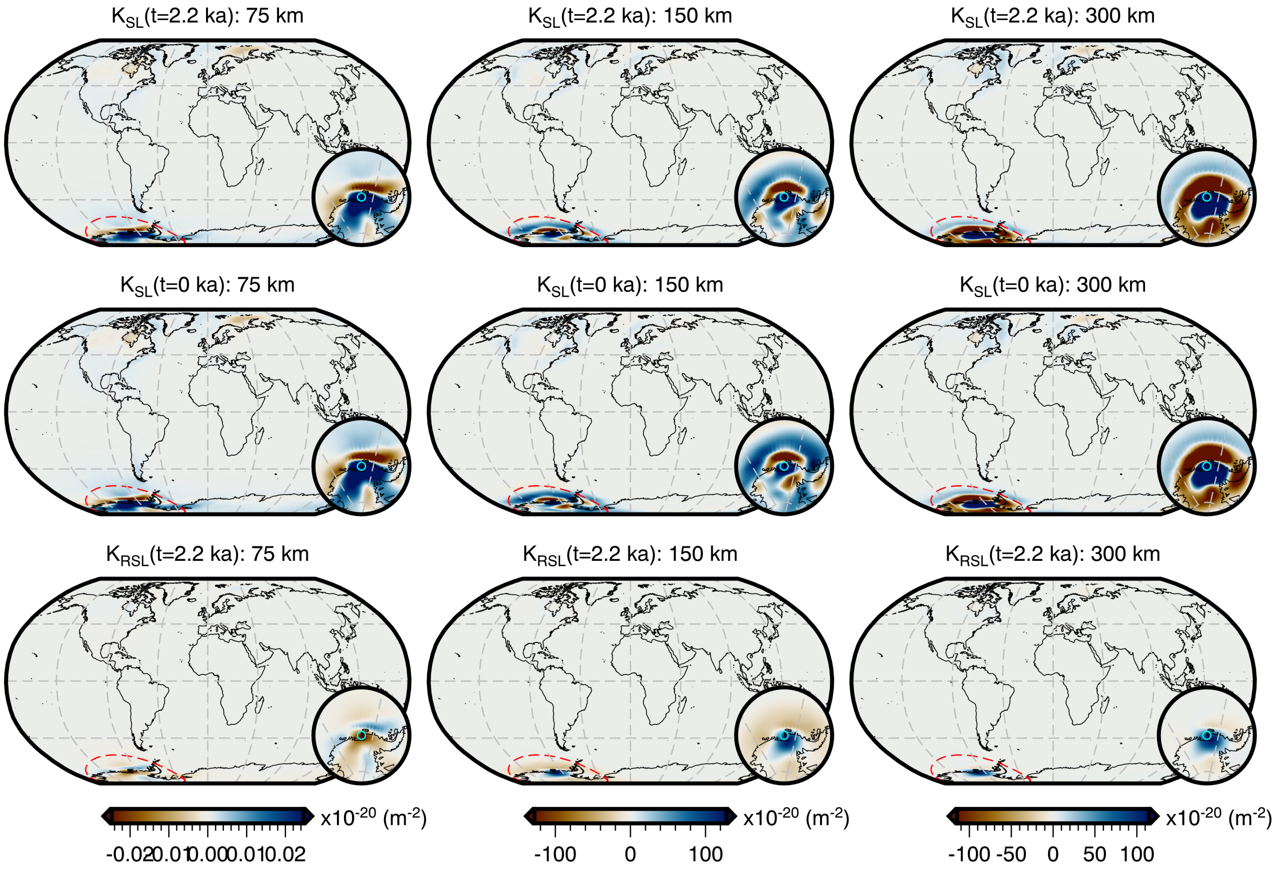

Supp. Figure S5: **Comparison of viscosity sensitivity kernels for sea-level and relative sea-level observations in the Amundsen Sea Embayment for a 1D viscosity structure.** Slices at 75, 150, and 300 km depth through the viscosity sensitivity kernels for (top row) a sea-level observation at 10 ka, (middle row) a sea-level observation at 0 ka, and (bottom row) a relative sea-level measurement at 10 ka. The inset map, centered on the observation site (cyan circle), has a width of 30° and it's extent is shown by the red dashed line the main map. The color scale for each column is chosen to symmetrically span the full range of relative sea-level viscosity sensitivity kernel and thus, regions of the sea-level sensitivity kernels may be saturated.

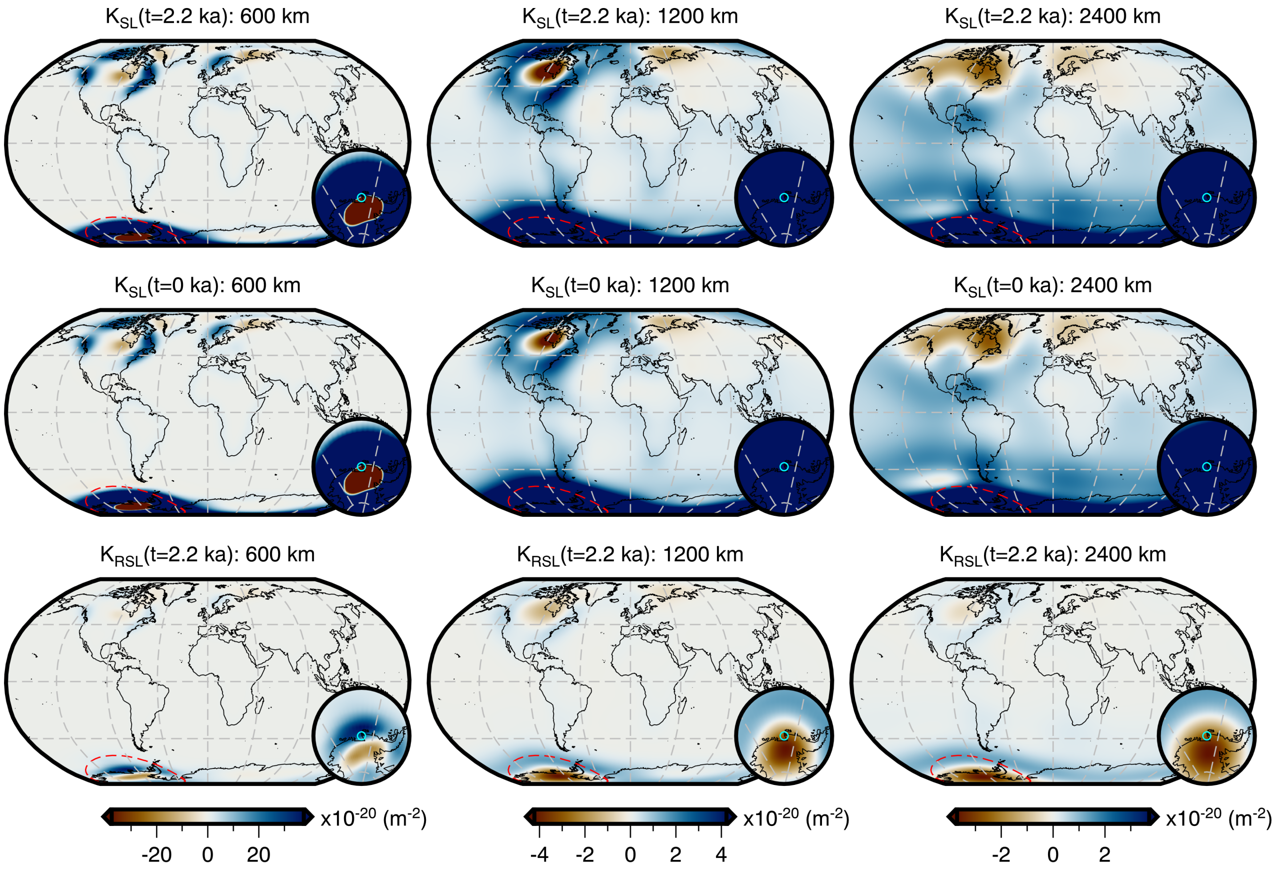

Supp. Figure S6: **Comparison of viscosity sensitivity kernels for sea-level and relative sea-level observations in the Amundsen Sea Embayment for a 1D viscosity structure.** Panels are the same as Figure S5, but for slices at 600, 1200, and 2400 km depth.

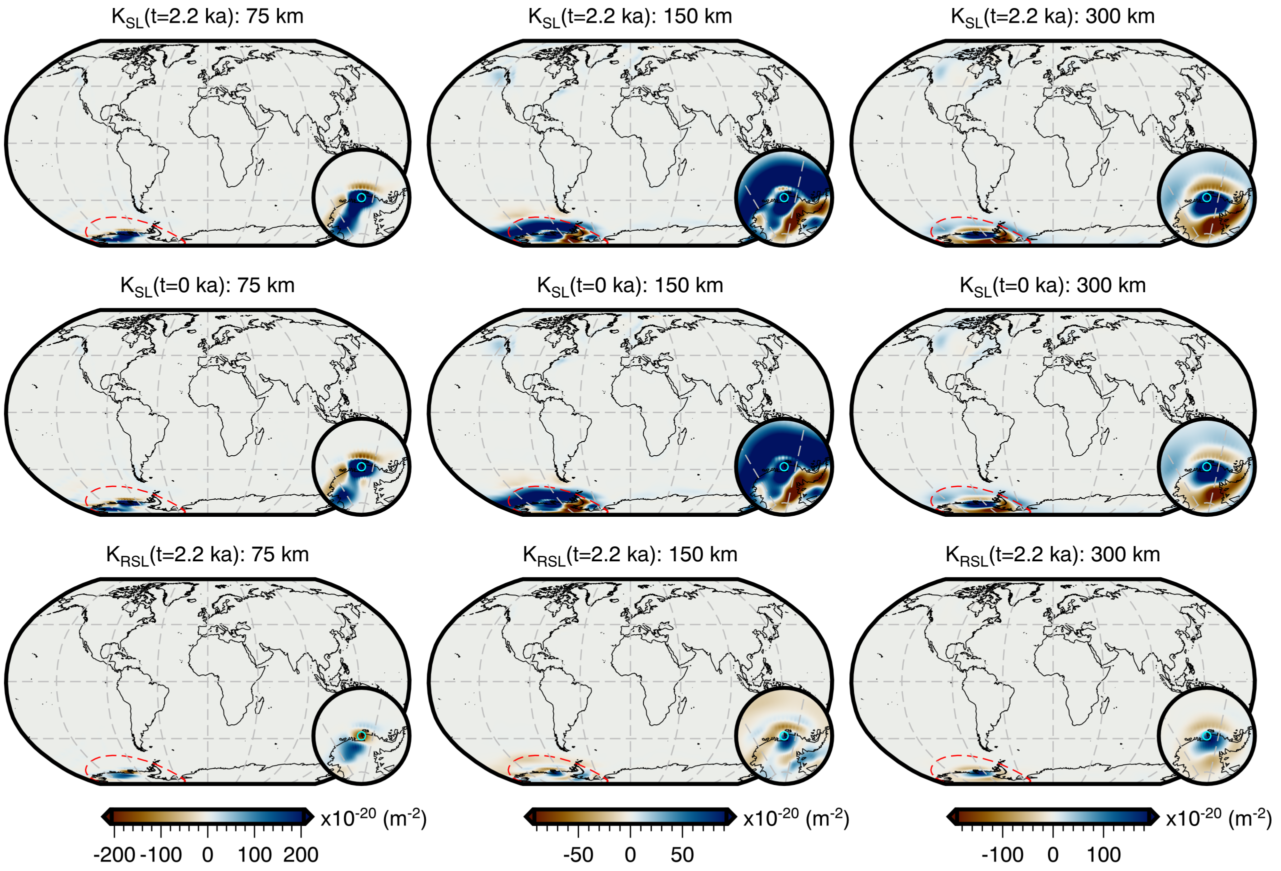

Supp. Figure S7: **Comparison of viscosity sensitivity kernels for sea-level and relative sea-level observations in the Amundsen Sea Embayment for a 3D viscosity structure.** Panels are the same as Figure S5, but now we have used our filtered and bounded 3D viscosity inference (Figure 2).

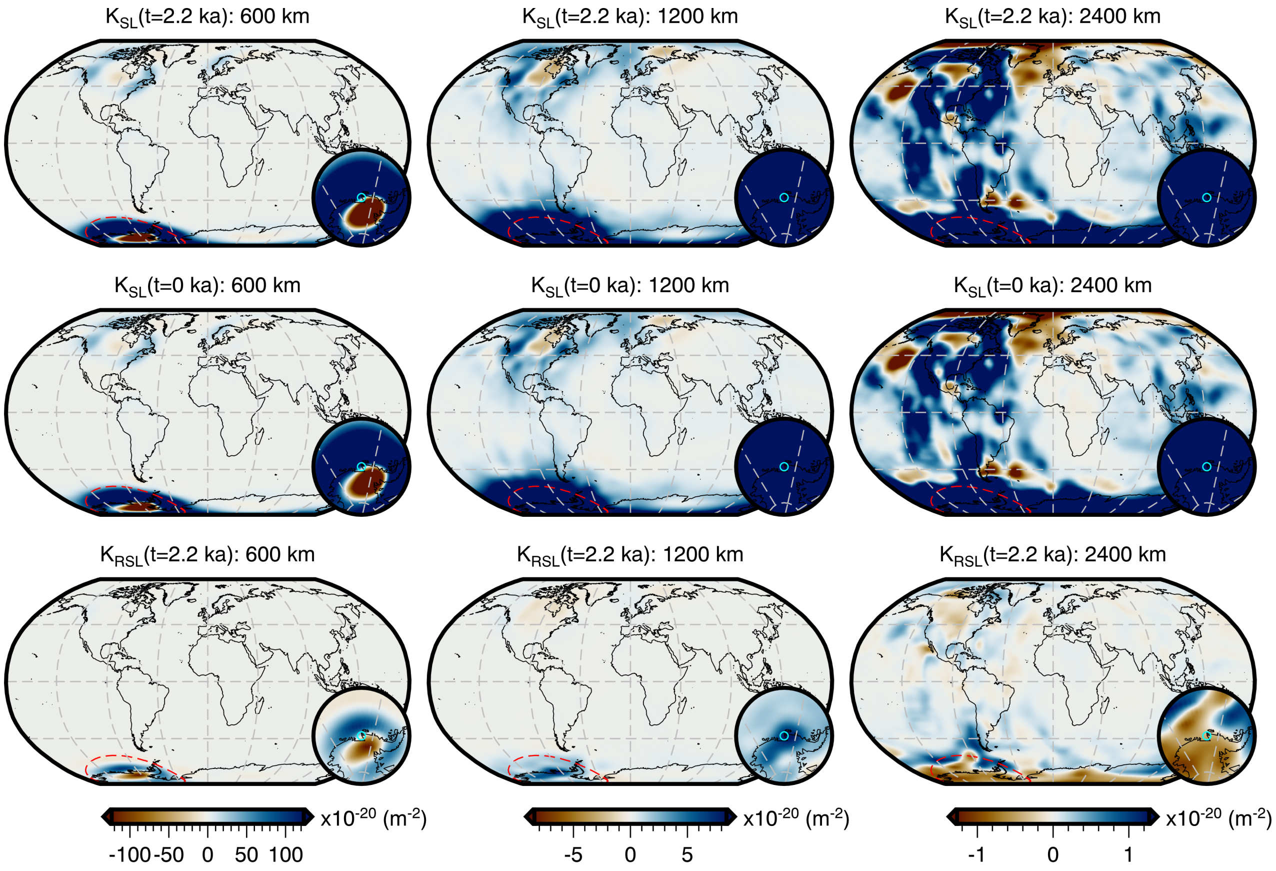

Supp. Figure S8: **Comparison of viscosity sensitivity kernels for sea-level and relative sea-level observations in the Amundsen Sea Embayment for a 3D viscosity structure.** Panels are the same as Figure S7, but for slices at 600, 1200, and 2400 km depth.

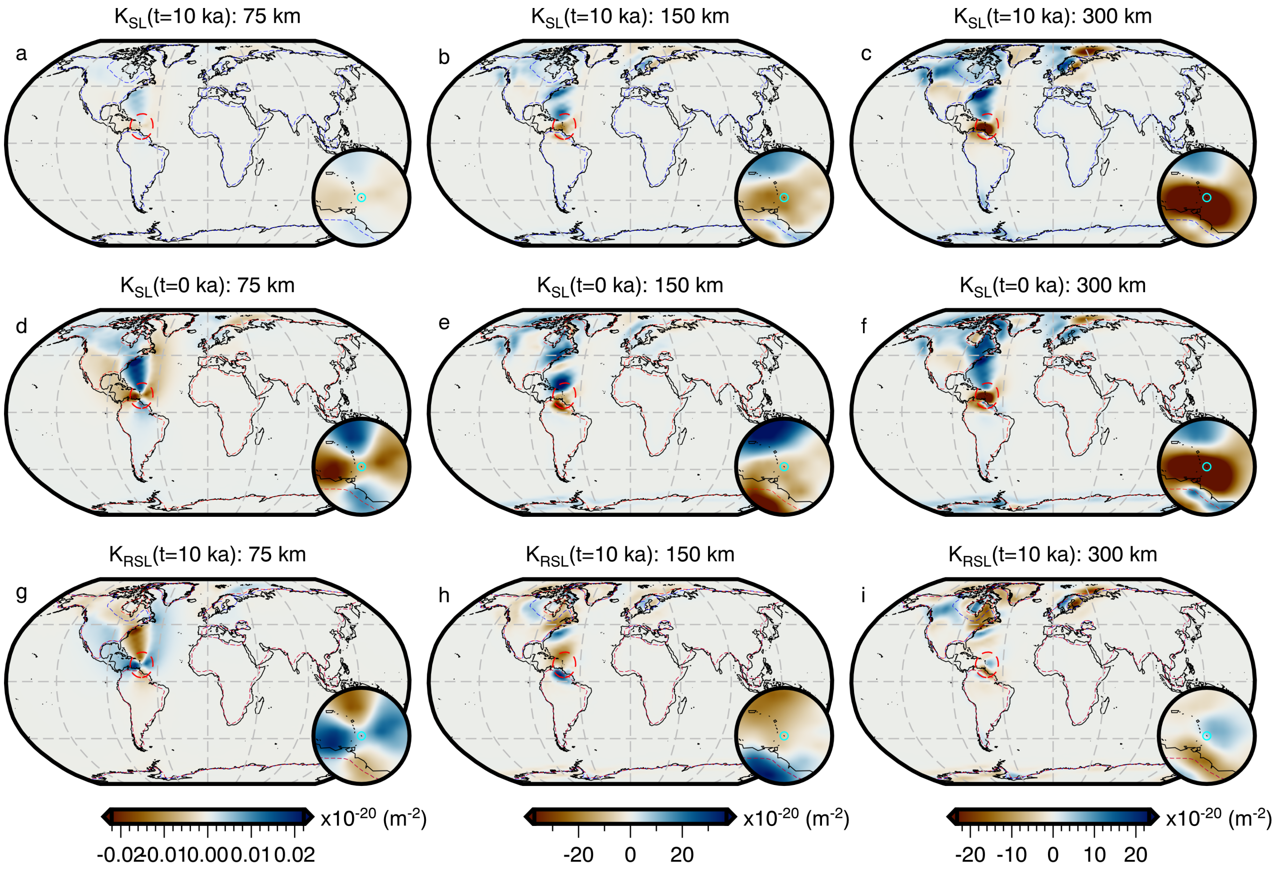

Supp. Figure S9: **Comparison of viscosity sensitivity kernels for sea-level and relative sea-level observations at Barbados for a 1D viscosity structure.** Panels are the same as Figure S5, but now the observation site is at Barbados and the width of inset map is 20°.

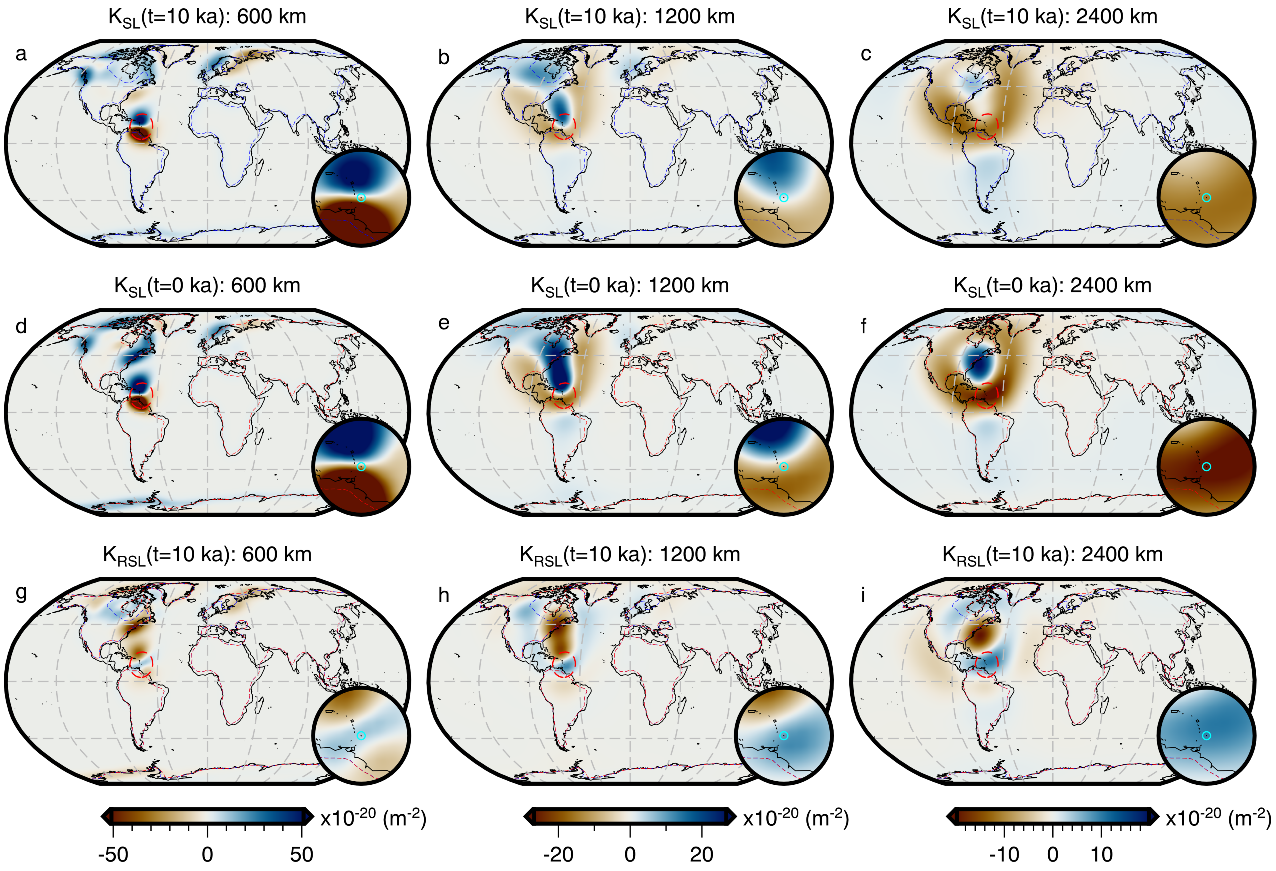

Supp. Figure S10: **Comparison of viscosity sensitivity kernels for sea-level and relative sea-level observations at Barbados for a 1D viscosity structure.** Panels are the same as Figure S9, but for slices at 600, 1200, and 2400 km depth.

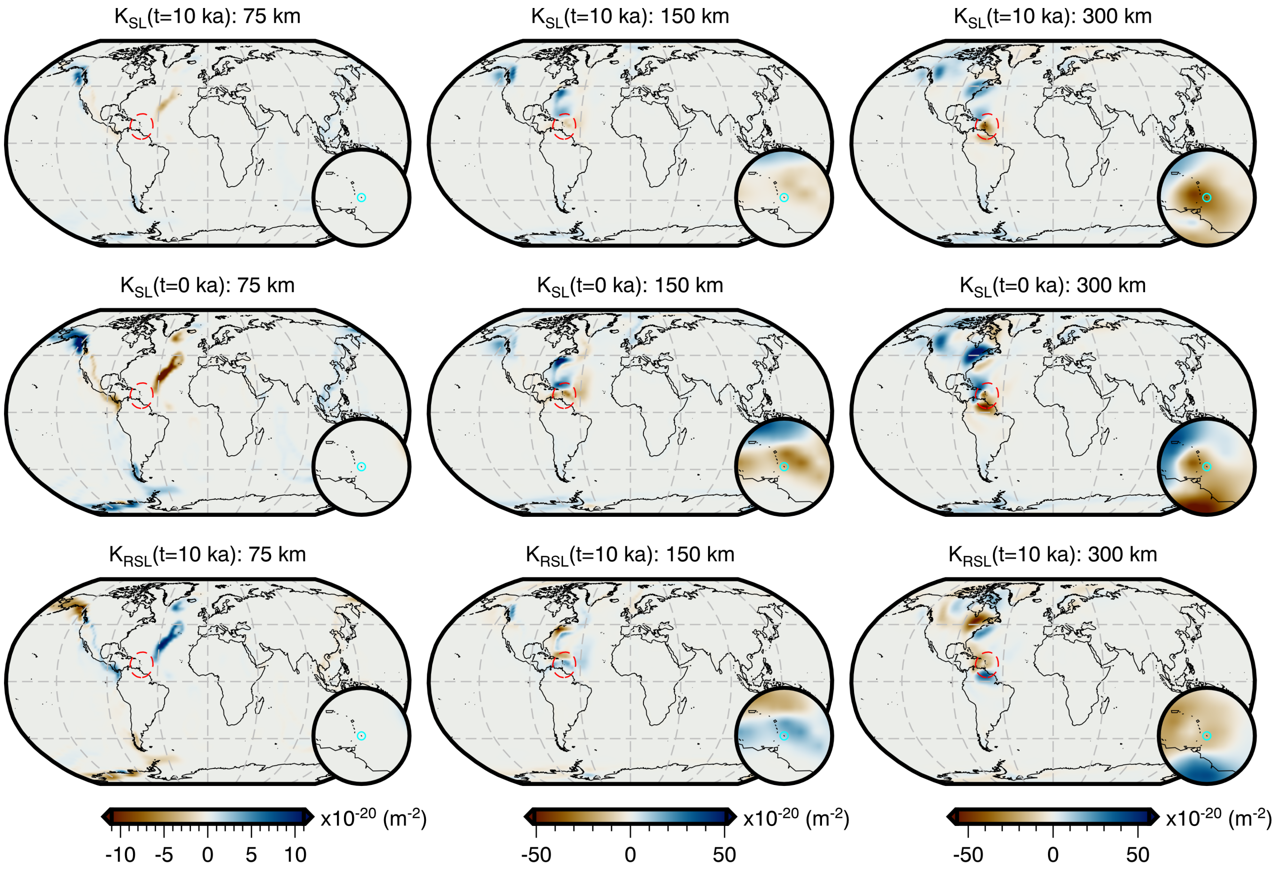

Supp. Figure S11: **Comparison of viscosity sensitivity kernels for sea-level and relative sea-level observations at Barbados for a 3D viscosity structure.** Panels are the same as Figure S5, but now we have used our filtered and bounded 3D viscosity inference (Figure 2) and the width of inset map is 20°.

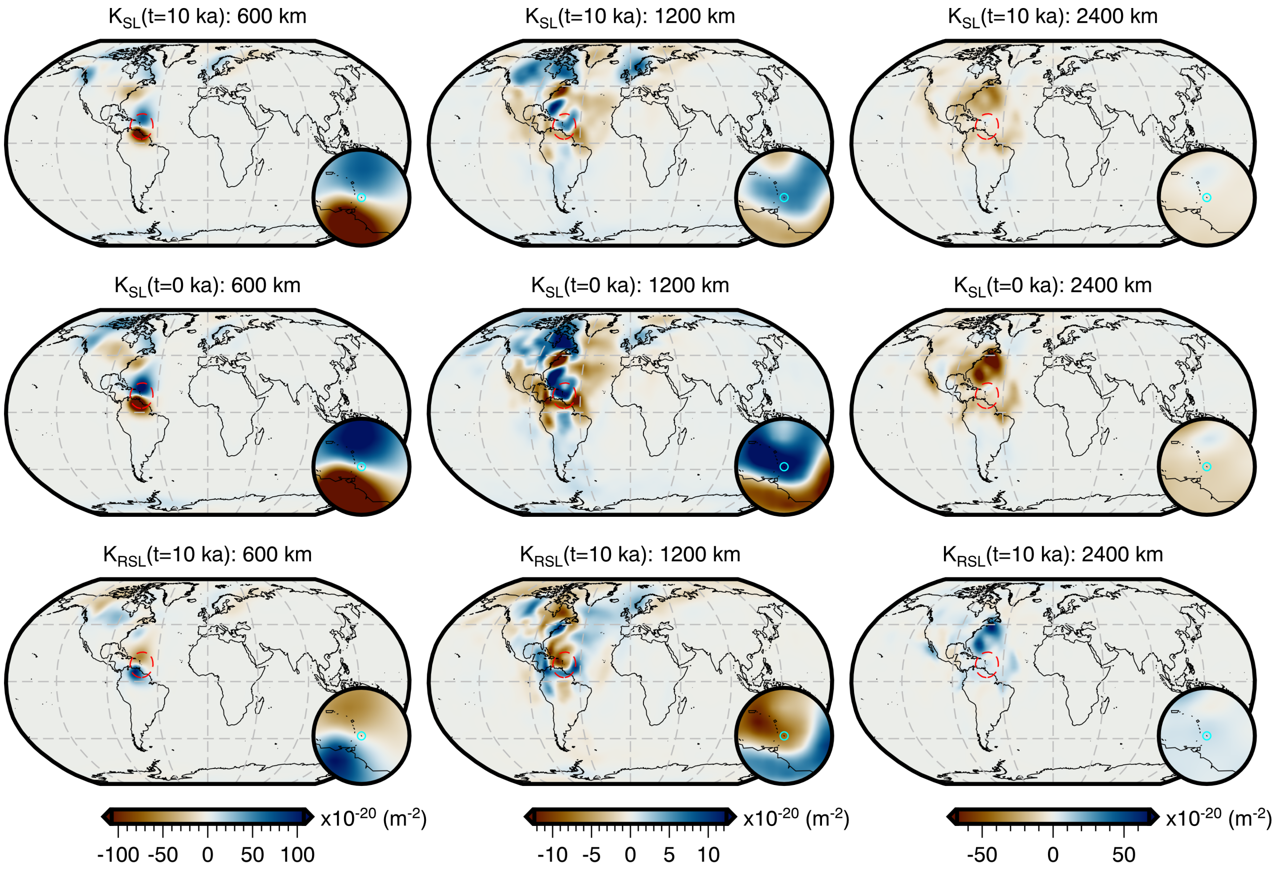

Supp. Figure S12: **Comparison of viscosity sensitivity kernels for sea-level and relative sea-level observations at Barbados for a 3D viscosity structure.** Panels are the same as Figure S11, but for slices at 600, 1200, and 2400 km depth.

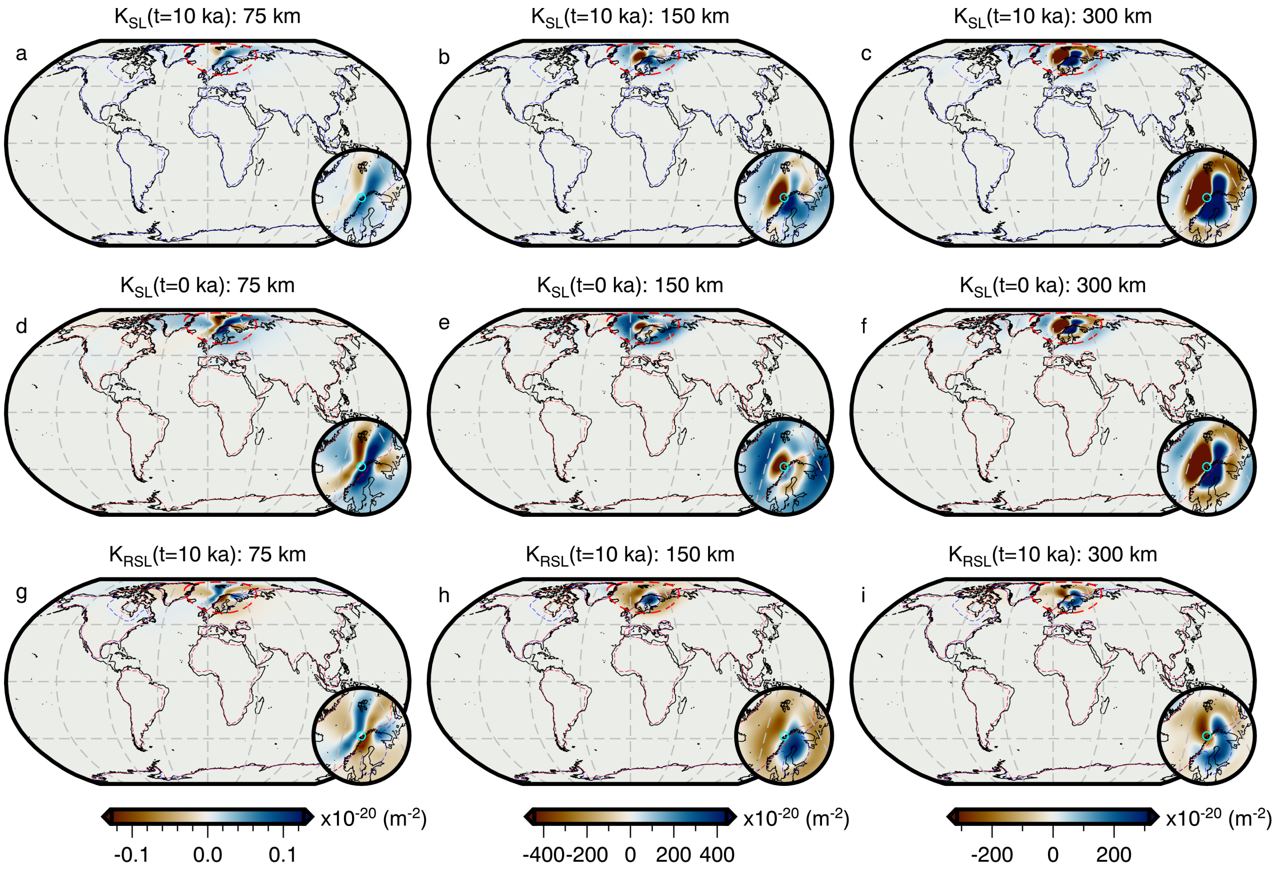

Supp. Figure S13: **Comparison of viscosity sensitivity kernels for sea-level and relative sea-level observations at Andenes,Norway for a 1D viscosity structure.** Panels are the same as Figure S5, but now the observation site is at Andenes, Norway and the width of inset map is 30°.

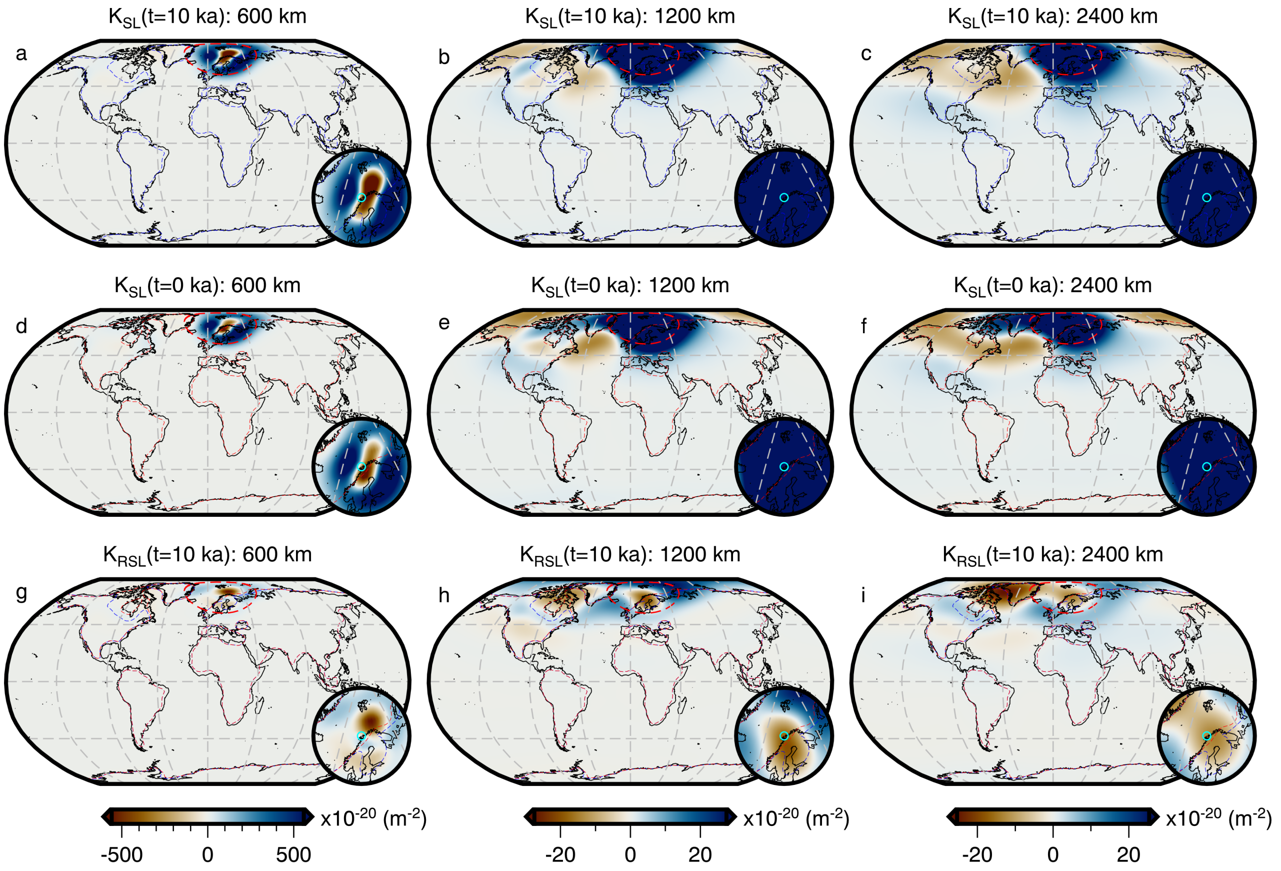

Supp. Figure S14: **Comparison of viscosity sensitivity kernels for sea-level and relative sea-level observations at Andenes, Norway for a 1D viscosity structure.** Panels are the same as Figure S13, but for slices at 600, 1200, and 2400 km depth.

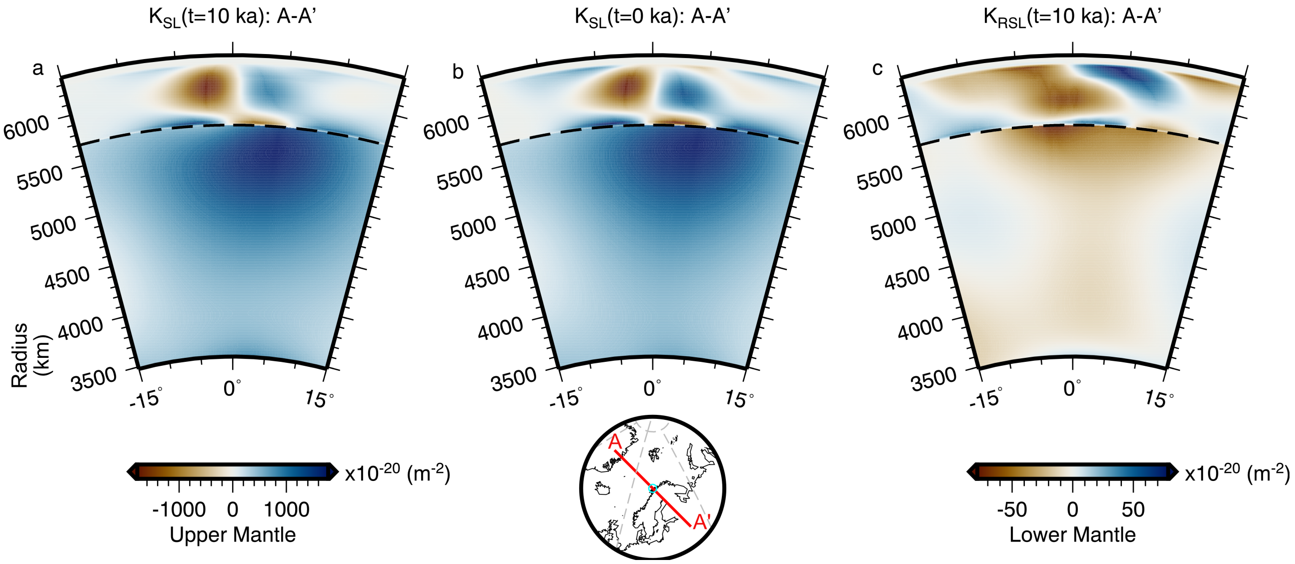

Supp. Figure S15: **Profile A–A' Comparison of viscosity sensitivity kernels for sea-level and relative sea-level observations at Andenes, Norway for a 1D viscosity structure.** A radial slice along profile A–A' through the viscosity sensitivity kernels for (*a*) an absolute sea-level observation at 10 ka,(*b*) an absolute sea-level observation at 0 ka, and (*c*) a relative sea-level measurement at 10 ka. The location of this profile is shown on the bottom center map, which is centered on the observations site (cyan circle). In the radial cross-sections the black dashed line shows the 670 km discontinuity. Above this discontinuity the values of the kernel correspond to the colour scale in the lower-left corner. Those values in the lower mantle are coloured using the colour scale in the lower-right corner. The color scales are chosen to symmetrically span the full range of relative sea-level viscosity sensitivity kernel in these two regions and thus, regions of the sea-level sensitivity kernels may be saturated.

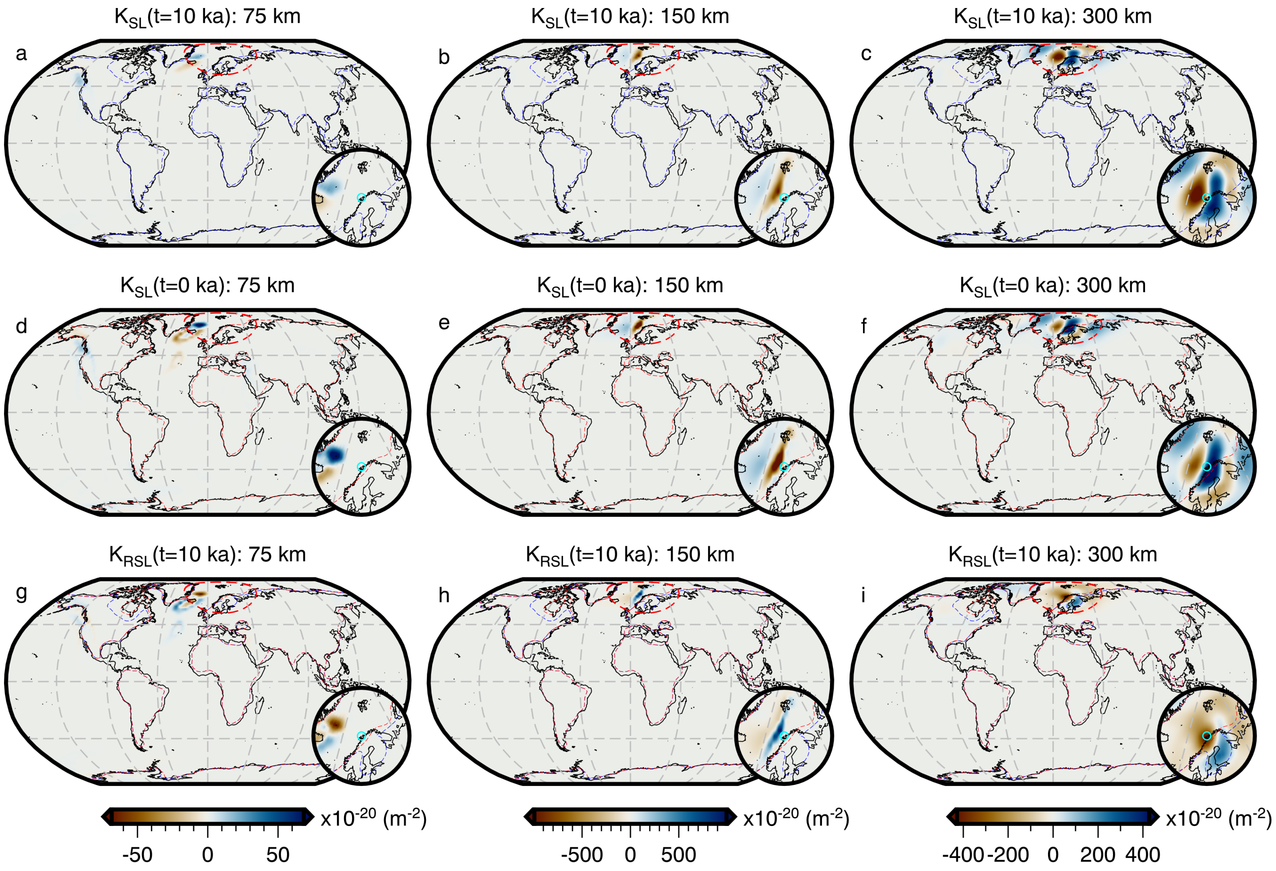

Supp. Figure S16: **Comparison of viscosity sensitivity kernels for sea-level and relative sea-level observations at Andenes, Norway for a 3D viscosity structure.** Panels are the same as Figure S5, but now we have used our filtered and bounded 3D viscosity inference (Figure 2) and the width of inset map is 30°.

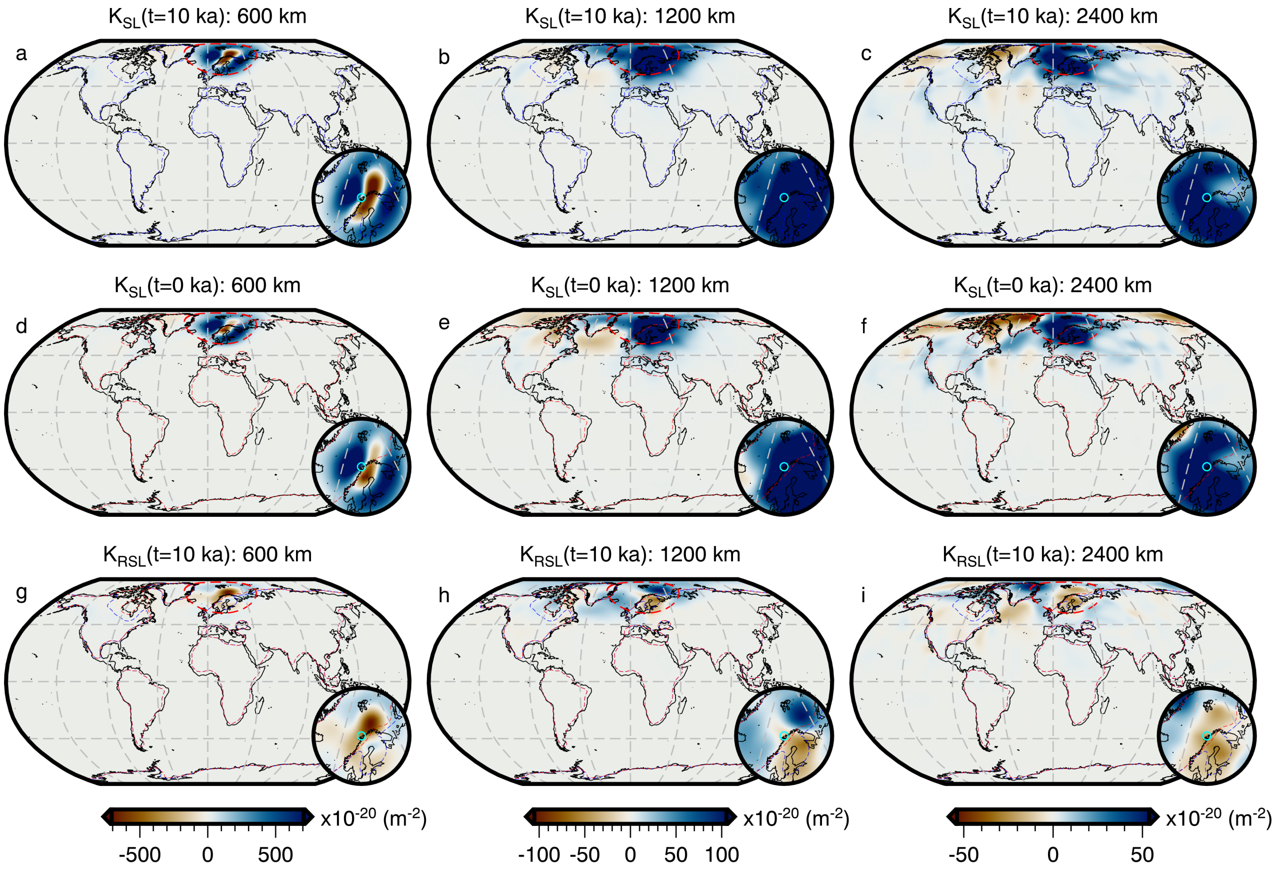

Supp. Figure S17: **Comparison of viscosity sensitivity kernels for sea-level and relative sea-level observations at Andenes, Norway for a 3D viscosity structure.** Panels are the same as Figure S16, but for slices at 600, 1200, and 2400 km depth.

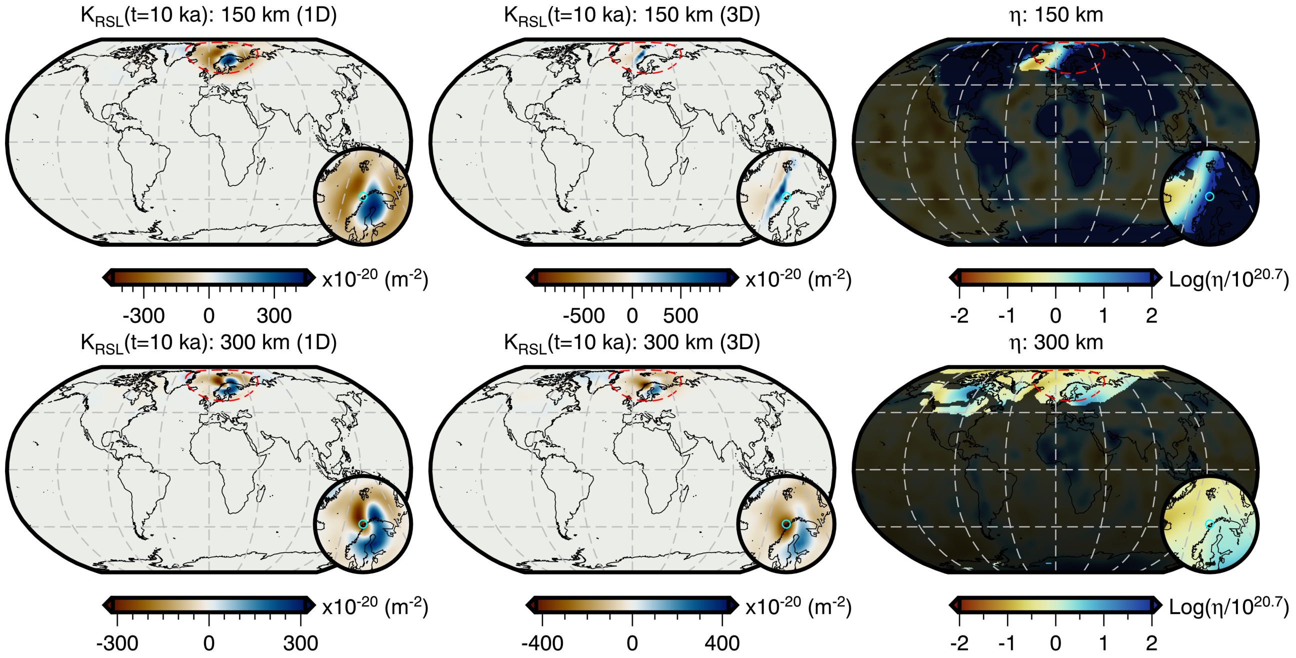

Supp. Figure S18: **Relative sea-level viscosity sensitivity kernels for 1D and 3D viscosity structure.** Slices at 150 km and 300 km depth through the viscosity sensitivity kernels for a relative sea-level observation on at Andenes, Norway (cyan circle) dating to 10 ka. The first column shows the sensitivity kernel obtained when assuming our 1D viscosity model (Section 5.1 and Supp. Figure S4) and the second column shows the sensitivity kernel obtained when assuming our filtered and bounded 3D viscosity inference (Figure 2). It is this 3D viscosity structure that is shown in the third column and regions where the amplitude of the sensitivity kernel are less than 0.1% of the maximum amplitude of the kernel are shaded in grey. The inset map is centered on Andenes and has a width of 30°.

References

Bozdağ, E., Peter, D., Lefebvre, M., Komatitsch, D., Tromp, J., Hill, J., Podhorszki, N., & Pugmire, D., 2016. Global adjoint tomography: first-generation model, *Geophysical Journal International*, **207**(3), 1739–1766.

Lei, W., Ruan, Y., Bozdağ, E., Peter, D., Lefebvre, M., Komatitsch, D., Tromp, J., Hill, J., Podhorszki, N., & Pugmire, D., 2020. Global adjoint tomography—model GLAD-m25, *Geophysical Journal International*, **223**(1), 1–21.
